# Novel (2-amino-4-arylimidazolyl)propanoic acids and pyrrolo[1,2-*c*]imidazoles via the domino reactions of 2-amino-4-arylimidazoles with carbonyl and methylene active compounds

**DOI:** 10.3762/bjoc.15.101

**Published:** 2019-05-06

**Authors:** Victoria V Lipson, Tetiana L Pavlovska, Nataliya V Svetlichnaya, Anna A Poryvai, Nikolay Yu Gorobets, Erik V Van der Eycken, Irina S Konovalova, Svetlana V Shiskina, Alexander V Borisov, Vladimir I Musatov, Alexander V Mazepa

**Affiliations:** 1 SSI “Institute for Single Crystals” of National Academy of Sciences of Ukraine, Nauky Ave. 60, Kharkiv 61074, Ukraine; 2 Antidiabetic Drug Laboratory, SI “V.Ya. Danilevsky Institute for Endocrine Pathology Problems”, Academy of Medical Sciences of Ukraine, 10, Alchevskih St., Kharkiv 61002, Ukraine; 3 Chemistry Department, V.N. Karazin Kharkov National University, Kharkov 61022, Ukraine; 4 Laboratory for Organic & Microwave-Assisted Chemistry (LOMAC), Katholieke Universiteit Leuven, Celestijnenlaan 200F, B-3001 Leuven, Belgium; 5 Peoples' Friendship University of Russia (RUDN University) 6 Miklukho-Maklaya street, Moscow, 117198, Russia; 6 Enamine Ltd., 23 Chervonotkats’ka str., Kyiv 01003, Ukraine; 7 A.V. Bogatsky physico-chemical institute of the National Academy of Sciences of Ukraine, 86, Lustdorfskaya doroga, 65080, Odessa, Ukraine

**Keywords:** 2-amino-4-arylimidazole, (2-amino-4-arylimidazolyl)propanoic acid, isatin, Meldrum’s acid, multicomponent reactions, pyrrolo[1,2-*c*]imidazole, 3,3’-spirooxindoles

## Abstract

The unexpectedly uncatalyzed reaction between 2-amino-4-arylimidazoles, aromatic aldehydes and Meldrum’s acid has selectively led to the corresponding Knoevenagel–Michael adducts containing a free amino group in the imidazole fragment. The adducts derived from Meldrum’s acid have been smoothly converted into 1,7-diaryl-3-amino-6,7-dihydro-5*H*-pyrrolo[1,2-*c*]imidazol-5-ones and 3-(2-amino-4-aryl-1*H*-imidazol-5-yl)-3-arylpropanoic acids. The interaction of 2-amino-4-arylimidazoles with aromatic aldehydes or isatins and acyclic methylene active compounds has led to the formation of pyrrolo[1,2-*c*]imidazole-6-carbonitriles, pyrrolo[1,2-*с*]imidazole-6-carboxylates and spiro[indoline-3,7'-pyrrolo[1,2-*c*]imidazoles], which can be considered as the analogues of both 3,3’-spirooxindole and 2-aminoimidazole marine sponge alkaloids.

## Introduction

**Figure 1:**
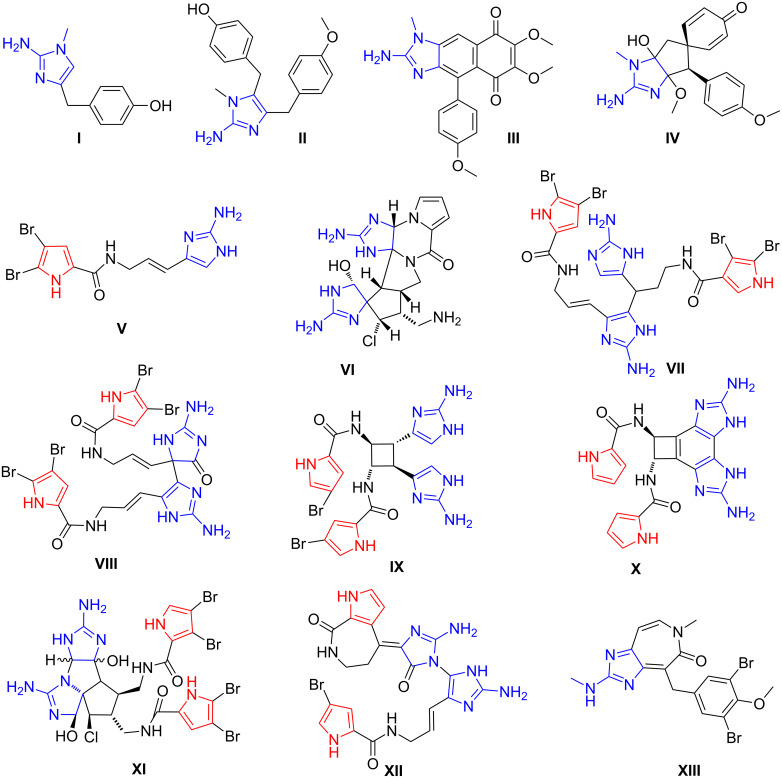
2-Aminoimidazole alkaloids from marine sponges.

Heterocyclic compounds of both natural and synthetic origin, containing in their structure pyrrole and imidazole rings, display a wide set of pharmacologically significant activities. The most important natural sources of such systems are marine sponges. Since the 70's of 20th century up to date more than 150 derivatives containing pyrrole and 2-aminoimidazole fragments in their structure were found among the metabolites of these marine organisms [1]. This group of compounds is characterized by an exceptional molecular diversity. The main structural types of these substances are shown in Figure 1. The metabolites of *Leucetta Sp*. and *Clathrina Sp*. are presented by achiral imidazole alkaloids from the group of benzyl substituted 2-aminoimidazole (dorimidazole A (**I**), naamine A (**II**)), fused cyclic systems (2-amino-2-deoxykealiiquinone (**III**)) and spiro-linked compounds ((−)-spirocalcaridine B (**IV**)) [2]. *Agelas Sp*. are a source of alkaloids with core structures containing simultaneously pyrrole carboxamide and 2-aminoimidazole moieties such as the simple achiral compound oroidine (**V**) and spatially organized molecules in a complex manner with a large number of chiral centres like (−)-palau’amine (**VI**) [3]. Oroidine (**V**) and other related vinyl 2-aminoimidazoles of this class are monomeric precursors of nagelamide A (**VII**), mauritiamine (**VIII**), sceptrin (**IX**), benzosceptrin A (**X**), axinellamines (**XI**) and stylissazole A (**XII**) alkaloids [1,4–5]. Fused 2-aminoimidazole and azepinone derivatives **XIII** were isolated recently from an extract of *Pseudoceratina Sp*. [6].

The variety of types of pharmacological activity revealed in these marine sponges’ metabolites is not inferior to the chemodiversity of their structure. Many of them are reported to have properties such as α-adrenoreceptors [7] and leukotriene B4 receptor antagonists [8], cyclin-dependent kinases GSK-3β, CK1 [9] and nitric oxide synthase activity inhibitors [10–11], as well as antibacterial [2], antifungal [12], antihistamine [13] and antitumor activities [14]. Remarkable immunosuppressive properties are inherent to palau’amine (**VI**) [15]. Ceratamines **XIII** are the disruptors of microtubule dynamics, therefore are of great interest in cancer drug discovery [6]. Thereby, the stereocontrolled total synthesis of marine alkaloids such as axinellamines [16] and the search of new 2-aminoimidazole and pyrrole containing compounds with a core structure that mimics metabolites of marine sponges with interesting biological properties has received considerable attention from both chemists and pharmacologists.

In the middle of 2000s, the authors of the studies [17–19] proposed a facile one-pot two-step procedure for the synthesis of diversely substituted 2-aminoimidazoles from α-bromocarbonyl compounds and substituted 2-aminopyrimidines. This methodology allowed the rapid synthesis of alkaloids of the isonaamine series [20] and other polysubstituted 2-aminoimidazoles with moderate cytostatic activity [21] and biofilm inhibitory activity against *S. Typhimurium* and *P. Aeruginosa* [22–23].

We have used 4-aryl-substituted 2-aminoimidazoles described by the authors of the aforementioned works as polyfunctional building blocks for the formation of different fused and spiro-linked heterocyclic systems. Last ones are able to act as precursors in the synthesis of the substances that mimic the core structure of marine alkaloids due to the presence of several reaction centres, which allow their further chemical modification. In the present work we disclose our results on the multicomponent reactions between 2-amino-4-arylimidazoles, aromatic aldehydes or isatins and cyclic or acyclic CH acids. As the last compounds we have used Meldrum’s acid, malononitrile and ethyl 2-cyanoacetate.

## Results and Discussion

**Figure 2:**
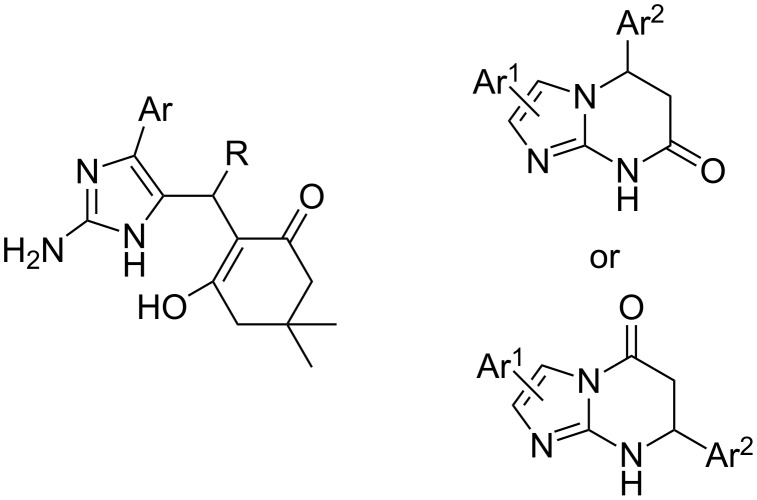
The Knoevenagel–Michael adduct [24] and expected products.

In view of the structure of 2-amino-4-arylimidazoles containing four nonequivalent nucleophilic centres several pathways can be assumed for their reactions with carbonyl 1,3-bielectrophiles or their synthetic precursors in the case of three-component reactions between these amines, carbonyl compounds and CH acids. Previously, an unusual direction of the three-component reaction between 2-aminoimidazoles, aldehydes and 5,5-dimethyl-1,3-cyclohexanedione has led to the formation of the Knoevenagel–Michael adducts (Figure 2) [24]. By analogy with our results obtained with the use of other aminoazoles in the reactions with benzaldehydes and Meldrum’s acid [25] we expected the formation of one or several isomers of tetrahydroimidazopyrimidinone derivatives (Figure 2).

However, a short time (3–5 min) and reflux of the equimolar amounts of amines **1**, *para*-substituted benzaldehydes **2**, and Meldrum’s acid **3** in 2-propanol led to Knoevenagel–Michael adducts **4a–h** (Table 1).

**Table 1:** Three-component condensation of 2-amino-4-arylimidazoles, aldehydes and Meldrum’s acid.

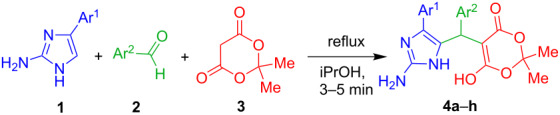				

Entry	Compound	Ar^1^	Ar^2^	Yield^a^, %

1	**4a**	Ph	Ph	77
2	**4b**	Ph	4-Me-C_6_H_4_	30
3	**4c**	4-Me-C_6_H_4_	Ph	48
4	**4d**	4-Me-C_6_H_4_	4-Me-C_6_H_4_	50
5	**4e**	4-Me-C_6_H_4_	4-NO_2_-C_6_H_4_	40
6	**4f**	4-F-C_6_H_4_	Ph	54
7	**4g**	4-F-C_6_H_4_	4-Me-C_6_H_4_	50
8	**4h**	4-Me-C_6_H_4_	3-OH-4-OMe-C_6_H_4_	70

^a^The isolated yields accounted on the quantities of the starting materials **1**–**3**.

Beside the short reaction times and mild conditions, this catalyst-free three-component condensation is characterized by a very facile performance since the solid products are formed as precipitates and are simply isolated in good yields without any additional purification (Table 1). In our synthetic practice this is the first example of the existence of stable β-adducts, which simultaneously contain Meldrum’s acid and aminoazole fragments. In all earlier described experiments with participation of different α-aminoazoles as binucleophiles the reaction cascade readily accomplished by the formation of fused heterocyclic systems [25].

**Scheme 1:**
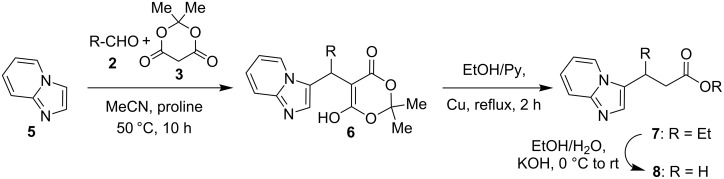
The three component condensation of imidazo[1,2-*a*]pyridine, aldehydes and Meldrum’s acid described by Gerencsér at al. [31].

An analogous three-component reaction involving indole or imidazo[1,2-*a*]pyridine derivatives instead of 2-aminoimidazoles is referred in the literature as Yonemitsu reaction or Yonemitsu-like reaction [26–31]. The similar Michael-type adducts **6** were isolated [31] from the reaction of imidazo[1,2-*a*]pyridine with aldehydes and Meldrum’s acid in acetonitrile in the presence of a catalytic amount of proline (Scheme 1) and then they were successfully converted to the appropriate esters **7** and acids **8**.

In our case, we have isolated products **4a–h** individually and characterized them by IR, ^1^H, ^13^C NMR, and mass-spectral methods. The ^1^H NMR spectra of products **4** have two characteristic broad singlets that represent the exchangeable proton shifts of the crossed signals of NH and OH groups at 12.35–11.61 ppm and the NH_2_ group of the aminoimidazole fragment at 7.47–7.26 ppm, as well as a singlet for the protons of two methyl groups. The existence of the dioxanedione cycle in enol form is proven by the presence of the singlet of a methyne proton near the saturated carbon atom at 5.42–5.56 ppm and the absence of the signal for the methyne proton of the dioxanedione cycle. With regard to the mass spectra, all compounds **4** exhibit similar behaviour in their fragmentation, showing the absence of the molecular ion peak and the presence of intense signals that occur due to cleavage of acetone and СО_2_ molecules from the dioxanedione moieties.

Their further transformation took place in the presence of the catalytic amounts of TFA in toluene under short time reflux (3 min) or addition of the catalytic amounts of TFA to the initial three-component mixture (Table 2).

**Table 2:** Synthesis of compounds **9b**,**c**,**i**, **10a**, **11b–g**.

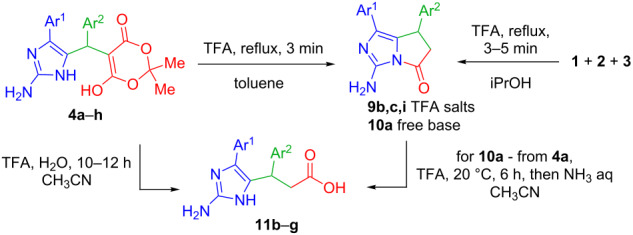				

Entry	Compound	Ar^1^	Ar^2^	Yield, %

1	**9b**	Ph	4-Me-C_6_H_4_	52
2	**9c**	4-Me-C_6_H_4_	Ph	60
3	**9i**	4-Ph-C_6_H_4_	Ph	71
4	**10a**	Ph	Ph	68
5	**11b**	Ph	4-Me-C_6_H_4_	55
6	**11c**	4-Me-C_6_H_4_	Ph	76
7	**11d**	4-Me-C_6_H_4_	4-Me-C_6_H_4_	65
8	**11e**	4-Me-C_6_H_4_	4-NO_2_-C_6_H_4_	60
9	**11f**	4-F-C_6_H_4_	Ph	67
10	**11g**	4-F-C_6_H_4_	4-Me-C_6_H_4_	85

The reaction proceeded via 1,3-dioxanedione cycle cleavage followed by elimination of acetone and CO_2_ to provide novel pyrrolo[1,2-*c*]imadazol-5-ones trifluoroacetates **9b,c,i** precipitated from the reaction mixture (Table 2). The corresponding bicyclic amine **10a** was obtained by the prolonged treatment of the product **4a** with catalytic amounts of TFA in acetonitrile followed by the addition of an aqueous solution of NH_3_.

The structures of cyclized products **9** and **10** were confirmed by spectral methods. The signals of NH, OH and methyl groups of the dioxanedione cycle are absent in the ^1^H NMR spectra of trifluoroacetates **9**. The broad signal of the NH_2_ group shifts to the downfield signal of NH_3_^+^ at 8.5–8.2 ppm. Protons of the СН_2_–СН fragment in the pyrrolidine cycle show the shifts of an ABX system for СН_Х_ at 4.73–4.85, СН_В_ at 3.81–3.84, СН_А_ at 2.86–2.95 ppm. The same situation is observed for compound **10a**, however, the signal of the free NH_2_ group of the aminoimidazole moiety shifts to 6.36 ppm. The common feature of the mass spectra of salts **9** is the absence of the salt molecular ion peak and the presence of the intense signals that occur due to cleavage of the CF_3_COO^−^ anion.

**Figure 3:**
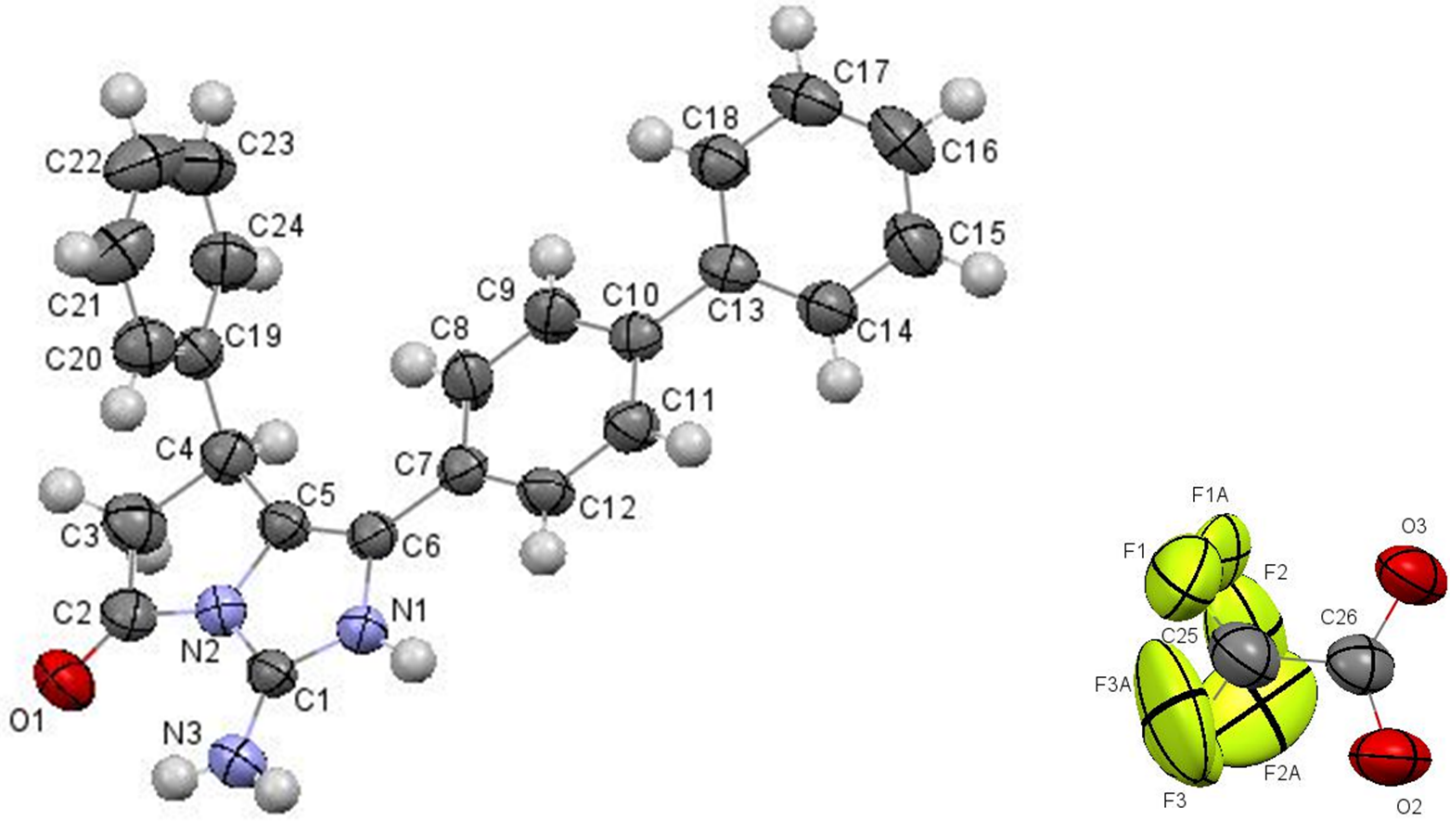
Molecular structure of 1-([1,1'-biphenyl]-4-yl)-5-oxo-7-phenyl-6,7-dihydro-5*H*-pyrrolo[1,2-*c*]imidazol-3-aminium 2,2,2-trifluoroacetate **9i** according to X-ray diffraction data. Thermal ellipsoids of atoms are shown at 50% probability level.

Single crystal X-ray diffraction analysis of biphenyl compound **9i** has finally proved the structures of the obtained products (Figure 3).

**Scheme 2:**
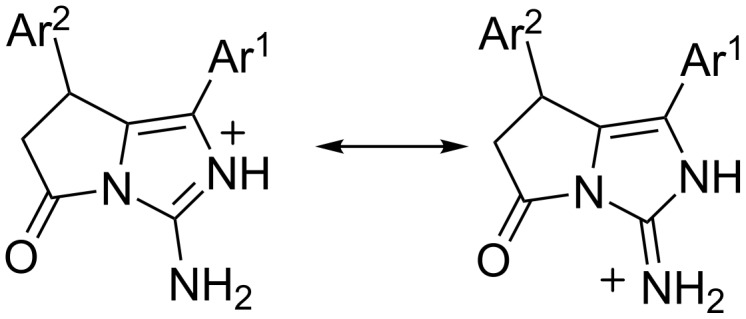
Two forms of cation **9i**.

Compound **9i** exists as organic salt with trifluoroacetic acid in the crystal phase. The existence of the trifluoroacetic molecule as anion is confirmed by close values of the C–O bond lengths (1.229(2) Å and 1.238(2) Å, respectively) and the absence of the hydrogen atom at the carboxylic group. The analysis of the bond lengths in the imidazole ring has revealed that the C1–N1 and C1–N3 bonds are equal (1.320(3) Å and 1.320(2) Å, respectively) and the N1–C6 bond (1.414(6) Å) is slightly elongated as compare to its mean value 1.376 Å [32]. The hydrogen atoms at the N1 and N3 were located from the electron density difference maps. As a result we may describe the structure of the organic cation as superposition of two forms (Scheme 2).

The prolonged reflux (10 h) of compounds **4b–g** in acetonitrile in the presence of a catalytic amount of TFA leads to the opening of the pyrrolidone ring followed by the formation of acids **11b–g** (Table 2). The process remarkably accelerates while adding water to the reaction mixture. The acids **11** can also be obtained from pyrrolo[1,2-*c*]imidazol-3-aminium trifluoroacetates **9** after prolonged reflux (12 h) in aqueous acetonitrile.

The ^1^H NMR spectra of acids **11** contain the signals of the protons of the aromatic system, the broad singlet for NH_2_ group at 5.87–5.82 ppm and the signals of the ABX protons of the propionyl fragment – СН_Х_ at 4.60–4.30 ppm and СН2_АВ_ at 3.00–2.60 ppm.

**Figure 4:**
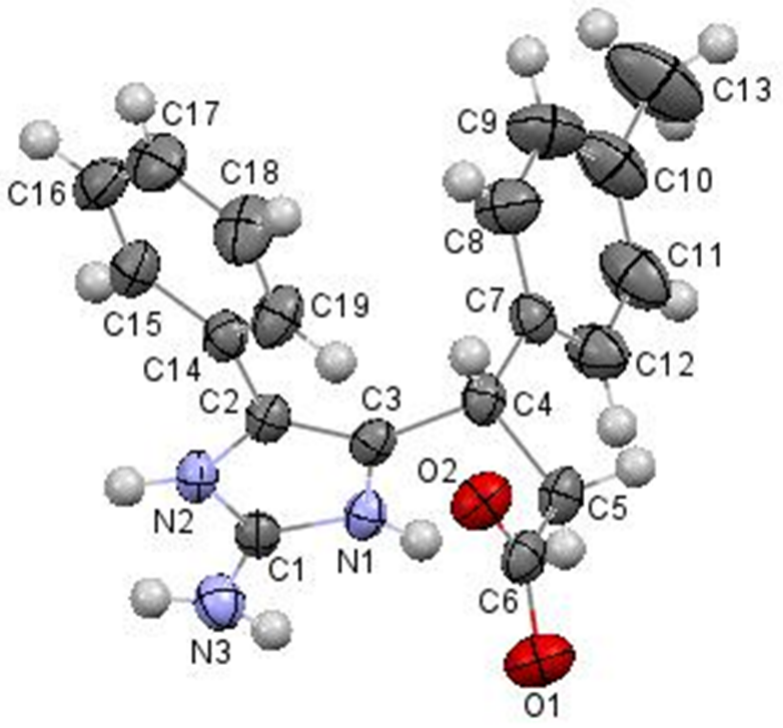
Molecular structure of 3-(2-amino-4-phenyl-1*H*-imidazol-5-yl)-3-(*p*-tolyl)propanoic acid **11b** according to X-ray diffraction data. Thermal ellipsoids of atoms are shown at 50% probability level.

Finally, the structure of acids **11** was confirmed by X-ray diffraction data of the sample compound **11b** (Figure 4).

**Scheme 3:**
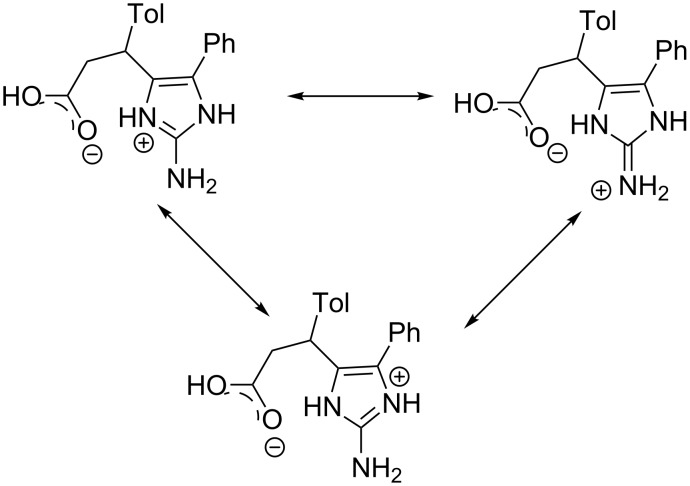
Three forms of the compound **11b** in the crystal phase.

Compound **11b** was found to be a zwitterion and exists as monohydrate in the crystal phase. The absence of the hydrogen atom and equalization of the C6–O1 and C6–O2 bond lengths (1.254(2) Å and 1.259(2) Å, respectively) allow presuming the location of the negative charge at the deprotonated carboxylic group. The very close lengths of the bonds centred at the C1 atom (the N2–C1 bond length is 1.332(2) Å, the C1–N3 bond length is 1.337(3) Å and the N1–C1 bond length is 1.340(2) Å) allows to describe the zwitterion as superposition of three forms with different location of the positive charge (Scheme 3).

Literatur data concerning pyrrolo[1,2-*c*]imidazol-5-ones is quite limited and the known 6,7-dihydro analogs are represented only by several substances [33–34]. Partially hydrogenated pyrrolo[1,2-*c*]imidazole is a part of (±)-axinellamines **11**. 4-[(5*R*)-6,7-Dihydro-5*H*-pyrrolo[1,2-*c*]imidazol-5-yl]-3-fluorobenzonitrile (LCI-699, osilodrostat) is considered as an inhibitor of aldosterone synthase (CYP11B2) and 11β-hydroxylase (CYP11B1), which is responsible for cortisol production [35]. This compound is under development for the treatment of Cushing's syndrome and pituitary ACTH hypersecretion [36]. From this point of view the approach to pyrrolo[1,2-*c*]imidazole moiety by using acyclic methylene active compounds, that can lead to cyclic products, has a high potential for diversity-oriented synthesis.

**Scheme 4:**
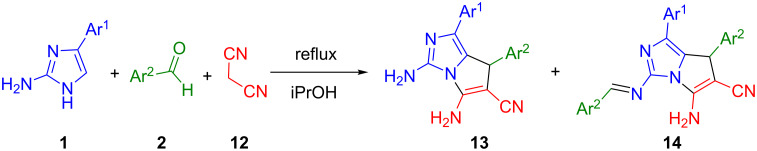
Synthesis of the mixture of compounds **13** and **14**.

In the three-component condensations of equimolar amounts of 2-amino-4-arylimidazoles **1**, *para*-substituted benzaldehydes **2** and malononitrile (**12**) in 2-propanol the Knoevenagel–Michael adduct was not obtained. The reaction was complete to form a mixture of pyrrolo[1,2-*c*]imidazol-6-carbonitriles **13** and their azomethine derivatives **14** (Scheme 4).

The use of a double excess of aromatic aldehydes **2** in this condensation prevented the formation of a mixture of substances and led to the formation of individual 5-amino-3-(arylidenamino)-1-aryl-7-aryl-7*H*-pyrrolo[1,2-*c*]imidazole-6-carbonitriles **14**, as well as azomethines **16** in case of using ethyl 2-cyanoacetate **15** as the acyclic methylene active compound (Table 3).

**Table 3:** Synthesis of compounds **14** and **16**.

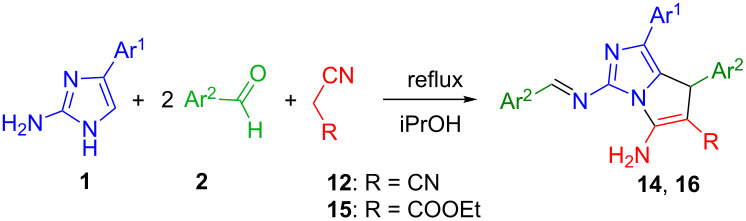				

Entry	Compound	Ar^1^	Ar^2^	Yield, %

1	**14a**	Ph	4-Me-C_6_H_4_	65
2	**14b**	4-Cl-C_6_H_4_	Ph	83
3	**14c**	4-OMe-C_6_H_4_	Ph	45
4	**14d**	Ph	4-Br-C_6_H_4_	40
5	**14e**	Ph	4-F-C_6_H_4_	58
7	**14f**	4-Me-C_6_H_4_	4-Br-C_6_H_4_	59
8	**16a**	Ph	Ph	30
9	**16b**	Ph	4-Br-C_6_H_4_	40

The isolated products **14a–f** and **16a**,**b** were characterized by IR, ^1^H, ^13^C NMR and mass-spectral methods. The mass spectra of compounds **14** and **16** show the similar type of fragmentation. They contain peaks of molecular ions, as well as signals corresponding to the loss of fragments [M^+•^ – NH_2_, – CN], [M^+•^ – ArCHN], [M^+•^ – NH_2_, – CN, – CAr]. From the comparison of these data with the results of elemental analysis, it follows that in the formation of condensed systems **14** with the participation of two molecules of aromatic aldehydes two molecules of water were cleaved. In the IR spectra, the most characteristic bands represent the absorption of NH_2_ groups at 3420 and 3332 cm^−1^ and the nitryl group CN at 2250 cm^−1^. In addition, there are characteristic bands at 1664–1668 cm^−1^, which may include both C=C bond and the exocyclic C=N bond. Fluctuations of endocyclic C=N fragments are observed at 1584 cm^−1^. Thus, at least one nitrile and one NH_2_ group are present in the obtained compounds. The ^1^H NMR spectra of compounds **14** along with protons of aryl substituents contain the characteristic singlet of the azomethine fragment at 9.24–9.34 ppm and a singlet of the methyne protone C^7^H at 5.26–5.36 ppm. Formation of the azomethine fragment during the interaction of the second molecule of the aromatic aldehyde with the C^2^–NH_2_ group of the imidazole moiety is confirmed by the disappearance of the singlet at 5.17–5.26 ppm, which is inherent to the NH_2_ group at the C^2^ position of the imidazole ring. Instead, in the spectra, a broad singlet of the C^5^–NH_2_ group of the imidazo[1,2-*c*]pyrrole cycle appears at 7.55–7.63 ppm. The absence in ^1^H NMR spectra of the signal of the CH of proton of the imidazole cycle at 6.97–7.10 ppm shows that the reaction takes place in the C^5^ nucleophilic centre of the aminoazole. The ^1^H NMR spectra of compounds **16** show the resonance of the ethyl group of the ethyl 2-cyanoacetate substituent as a triplet of a CH_3_ group at 1.02 ppm, *J* = 7.02 Hz and multiplets of the CH_2_ group at 3.85–4.03 ppm. The ^1^H NMR spectra of derivatives **16** are similar with the spectra of compounds **14** by the absence of the resonance of the NH_2_ group and methyne proton of the aminoimidazole ring, which allows to classify them as compounds of the same type containing a fused aminoimidazo[1,2-*c*]pyrrole moiety.

**Figure 5:**
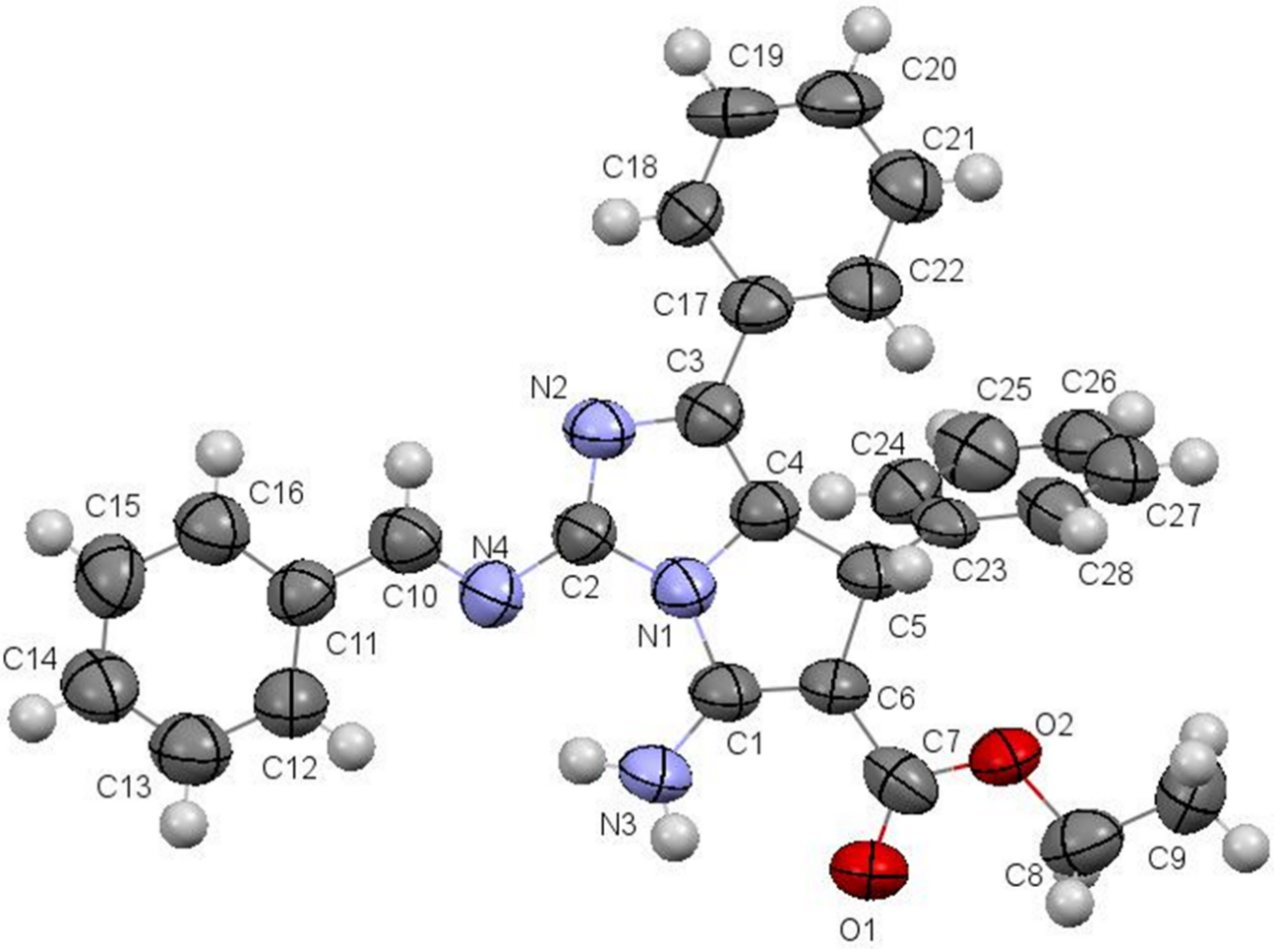
Molecular structure of aminoimidazo[1,2-*c*]pyrrole **16a** according to X-ray diffraction data. Thermal ellipsoids of atoms are shown at 50% probability level.

Finally, the structure of azomethines **16** was confirmed by X-ray diffraction data of the sample compound **16a** (Figure 5).

**Scheme 5:**
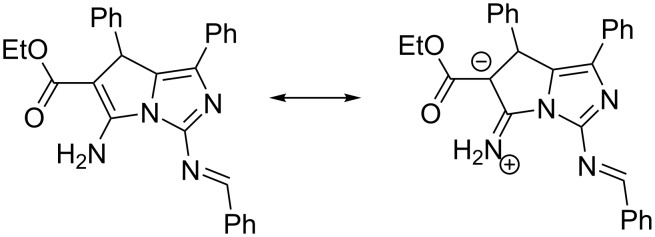
Resonance structures of **16a**.

All atoms of the bicyclic fragment lie in the plane within 0.01 Å. The analysis of the bond lengths has shown that the formally single exocyclic C1–N3 bond is shorter than the double endocyclic C6–C1 bond (1.336(6) Å and 1.354(9) Å, respectively).The C1 and C6 atoms are planar indicating their sp^2^ hybridization. Such a distribution of electron density allows to discuss the zwitter-ionic form and to consider the structure of **16a** as superposition of two resonance structures (Scheme 5).

**Figure 6:**
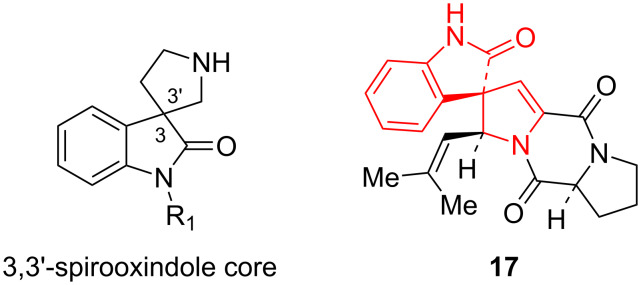
3,3’-Spirooxindole alkaloids.

In the next step of our research we have involved isatin **18** as the compound bearing a carbonyl group, as well in this case the pyrolo[1,2-*c*]imidazole moiety will be spiro-fused with the oxindole moiety, and the resulting structures can be considered as analogues of 3,3'-spiroxindole alkaloids, such as spirotryprostatin B (**17**, Figure 6) [37].

Indeed, the equimolar three-component reactions with the participation of 2-amino-4-arylimidazoles **1**, isatins **18** and acyclic methylene active compounds **12** and **15** have completed with the formation of spirooxindoles **19a–h** and **20a–c**, respectively, with moderate to high yields (Table 4). The reduced reactivity of the carbonyl group of isatins compared with benzaldehydes, and the greater stability of their Knoevenagel adducts leads to the formation of individual spiro compounds, not to a mixture of substances. However, condensations with the use of N-unsubstituted isatins are accompanied by the resinification of the reaction mixture, which may be caused by competing reactions of heterocyclization of the mentioned Knoevenagel adducts. In similar reactions described in the literature [38–40], the authors recognized the importance of protecting the amide fragment of isatin, since it affects the reactivity and, in some cases, the enantioselectivity of processes. In order to prevent undesirable side reactions in the future, three-component condensations were carried out using N-substituted isatins.

**Table 4:** Synthesis of spirooxindoles **19** and **20**.

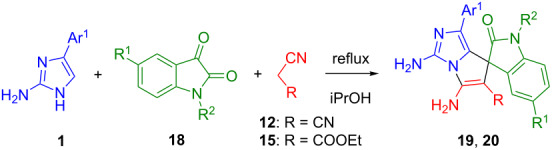					

Entry	Compound	Ar^1^	R^1^	R^2^	Yield, %

1	**19a**	Ph	H	Me	60
2	**19b**	Ph	Br	Me	68
3	**19c**	Ph	Me	Me	60
4	**19d**	Ph	F	4-ClC_6_H_4_CH_2_	40
5	**19e**	4-Me-C_6_H_4_	H	Me	68
7	**19f**	4-Me-C_6_H_4_	Br	Me	65
8	**19g**	4-F-C_6_H_4_	Br	Me	40
9	**19h**	4-Me-C_6_H_4_	Cl	Me	48
10	**20a**	Ph	H	Me	72
11	**20b**	4-OMe-C_6_H_4_	Br	Me	43
12	**20c**	4-Me-C_6_H_4_	Br	Me	60

The isolated products **19a–h** and **20a–c** were characterized by IR, ^1^H, ^13^C NMR and mass-spectral methods. The ^1^H NMR spectra of spirooxindoles **19a–h** and **20a–c**, along with protons of aryl substituents of imidazole and isatin contain a broad singlet with 2H intensity of the C^5^–NH_2_ group of the imidazo[1,2-*c*]pyrrole cycle at 7.74–7.84 ppm. A characteristic feature is the appearance of another broad singlet at the 6.37–6.46 ppm, inherent to the amino group of the C^2^ atom of the imidazole ring, whose chemical shift is affected by the character of the substituents in 2-amino-4-arylimidazoles. The multiplets of the С^6’^-ethoxy group of the compounds **20a–c** are seen at 0.62–0.88 (ОСН_2_*СН*_3_) and 3.57–3.85 ppm (О*СН*_2_СН_3_). The ^13^C NMR spectra of spirooxindoles **19a–h** and **20a–c** are represented by the groups of singlets at the 66.86–69.83 and 152.29–154.72 ppm. The signal of the spiro atom is seen as a singlet at 53.53–56.03 ppm. Signals of the carbon atoms of the imidazole ring are located in the resonance region of the carbon atoms of the aryl substituents. Taken together, these data indicate the formation of the pyrrolo[1,2-*c*]imidazole cyclic system.

**Figure 7:**
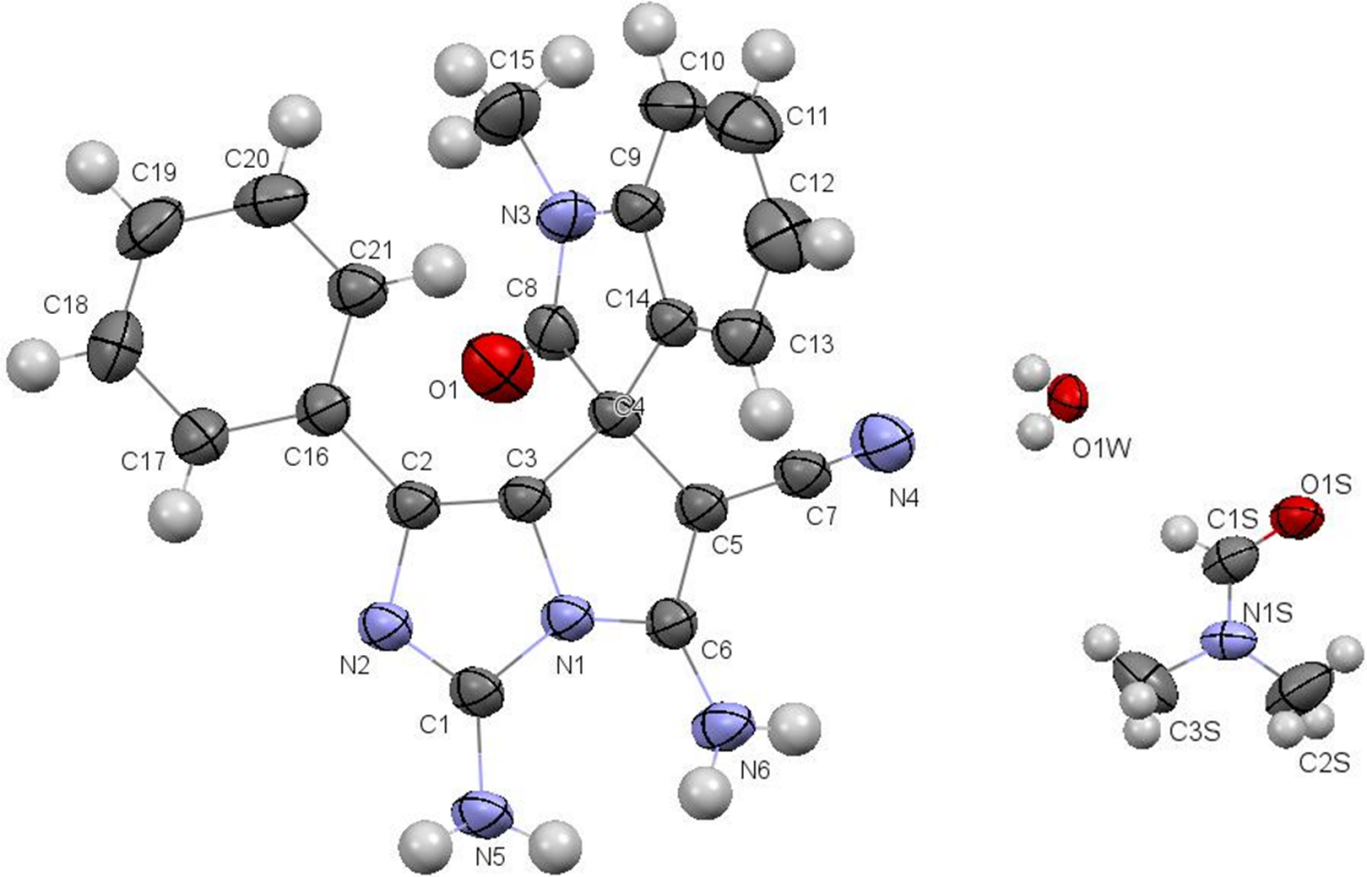
Molecular structure of aminoimidazo[1,2-*c*]pyrrole **19a** according to X-ray diffraction data. Thermal ellipsoids of atoms are shown at 50% probability level.

Finally, the structures of spiroxindoles **19** and **20** were confirmed by X-ray diffraction data of the sample compound **19a** (Figure 7).

Compound **19a** exists in the crystal phase as solvate with dimethylformamide and water in a 1:1:1 ratio.

**Scheme 6:**
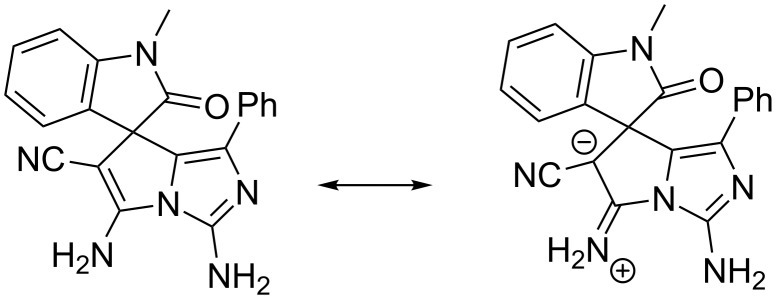
Resonance structures of **19a**.

The spiro-joined bicyclic fragments are turned relatively to each other in such a way that the dihedral angle between mean planes of the bicycles is 84.5°. The analysis of the bond lengths has shown that the formally single exocyclic C6–N6 bond is significantly shorter than the double endocyclic C6–C5 bond (1.319(2) Å and 1.373(3) Å, respectively). The C1 and C6 atoms are planar indicating their sp^2^ hybridization. Such a distribution of electron density allows discussing the zwitter-ionic form and considering the structure of **19a** as superposition of two resonance structures similar to **16a** (Scheme 6).

## Conclusion

In the described three-component reactions with aldehydes or isatins and cyclic or acyclic CH acids the C^5^ reaction centre in the 2-amino-4-arylimidazoles possesses higher nucleophilicity than both the exo- and endocyclic amino groups. Regarding the short reaction times of novel Yonemitsu-type reactions that has been achieved without application of any catalyst we assume that 2-amino-4-arylimidazoles are more reactive substrates for these syntheses leading to the stable Michael-type adducts with aldehydes and Meldrum’s acid than the previously investigated indole and imidazo[1,2-*a*]pyridine. Moreover, as it has been shown that their further transformations may result in the formation of both unexplored heterocyclic systems containing a free amino group open for chemical modifications and the corresponding hetarylpropanoic acids providing useful templates for the synthesis of some marine alkaloids or their analogues.

In domino reactions of the 2-amino-4-arylimidazoles with isatins and aliphatic CH acids stable Michael adducts have not been fixed. Cyclocondensation has readily led to the formation of 6'-substituted 3',5'-diamino-1-alkyl-2-oxo-1'-arylspiro[indolin-3,7'-pyrrolo[1,2-*c*]imidazoles], which can be considered as the analogues of alkaloids with both pyrrolo[1,2-*c*]imidazol and 3,3’-spiroxindole fragments in the core structure.

## Experimental

**Reagents and analytics:** Starting materials were purchased from commercial suppliers. Melting points were determined on a Kofler apparatus and temperatures were not corrected. The IR spectra were recorded in KBr on a Specord M-82 spectrometer. The ^1^H NMR spectra were measured on a Varian Mercury VX-200 (200 MHz) and Bruker AM-400 spectrometer (400 MHz), ^13^C NMR spectra were measured on a Bruker AM-400 (100 MHz) and Bruker Avance DRX 500 (125 МHz) spectrometers in DMSO-*d*_6_, CDCl_3_ and trifluoroacetic acid (TFA) using TMS as internal standard. The mass spectra were recorded on a Varian 1200L GC–MS instrument, ionization by EI at 70 eV. Fast atom bombardment (FAB) mass spectrometry was performed on a VG 70-70EQ mass spectrometer, equipped with an argon primary atom beam, and a *m*-nitrobenzyl alcohol matrix was used. LC–MS experiments were performed on an Applied Biosystems (Shimadzu 10-AV LC, Gilson-215 automatic giving, mass spectrometer API 150EX, detectors UV (215 and 254 nm), and ELS, column Luna-C18, Phenomenex, 5 µ, 100 Angstrom, 150 × 2 mm RP). Elemental analyses were made on an elemental analyzer Euro AE-3000. The progress of reactions and the purity of the obtained compounds were monitored by TLC on Silufol UV-254 plates in EtOAc/CH_2_Cl_2_ (1:4) and visualized under UV light or iodine fume.

**General procedure for the synthesis of 5-((2-amino-4-aryl-1*H*-imidazol-5-yl)(aryl)methyl)-6-hydroxy-2,2-dimethyl-4*H*-1,3-dioxin-4-ones:** An equimolar mixture (1.0 mmol) of the corresponding 2-amino-4-arylimidazole **1**, aromatic aldehyde **2** and Meldrum’s acid **3** was refluxed in iPrOH (3 mL) for 3–5 min. After cooling, the solid products **4** were filtered off, washed with iPrOH and dried on air. **4a**: colourless solid, 77%; mp 243–245 °C; IR (KBr, cm^−1^) ν: 3404–2800 (NH_2_, NH, OH), 1684 (C=O); ^1^H NMR (200 MHz, DMSO-*d*_6_) δ 12.27 (br s, 2H, NH, OH), 7.61–7.49 (m, 2H, H_arom_), 7.48–7.31 (m, 5H, H_arom_), 7.27–7.01 (m, 5H, NH_2_,_,_ H_arom_), 5.48 (s, 1H, CH), 1.51 (s, 6H, CH_3_); ^13^C NMR (125 MHz, CDCl_3_) δ 166.8 (C=O), 146.6 (C-OH), 144.0, 129.4, 128.9, 128.4, 128.3, 127.5, 127.4, 127.2, 125.9, 121.1, 100.6, 76.0 (*C*=COH), 35.1 (CH), 26.4 (CH_3_), 25.9 (CH_3_); MS (*m*/*z*) (%): 289 (76), (391 [M^+•^] – 44 −58), 247 (53), 159 (36), 104 (19), 77 (8), 44 (39), 43 (100); anal. calcd for C_22_H_21_N_3_O_4_ (391.5) C, 67.52; H, 5.37; N, 10.74; found: C, 68.77; H, 5.93; N, 10.79.

**General procedure for the synthesis of 5-oxo-1,7-diaryl-6,7-dihydro-5*H*-pyrrolo[1,2-*c*]imidazol-3-aminium 2,2,2-trifluoroacetates:** A mixture of the corresponding adduct **4** (0.1 mmol) and 0.08 mL (0.11 mmol) TFA was refluxed in 1 mL of toluene for 3 min. After cooling, 3 mL of iPrOH was added to the reaction mixture and the solid product **9** was filtered off, washed with iPrOH and dried on air. **9b**: colourless solid, 52%; mp 222–224 °C; IR (KBr, cm^−1^) ν: 3432–3160 (NH_3_^+^, COO^−^), 1782 (C=O); ^1^H NMR (400 MHz, DMSO-*d*_6_) δ 8.54 (br s, 2H, NH_3_^+^), 7.29–7.16 (m, 6H, H_arom_), 7.15–7.03 (m, 3H, H_arom_), 4.81 (d, *J* = 3.6 Hz, 1H, CH_X_), 3.81 (dd, *J*_BX_ = 9.3 Hz, *J*_AB_ = 18.6 Hz, 1H, CH_B_), 2.91 (d, *J*_AX_ = 3.8 Hz, *J*_AB_ = 18.7 Hz, 1H, CH_A_), 2.20 (s, 3H, CH_3_); ^13^C NMR (100 MHz, DMSO-*d*_6_) δ 170.2 (C=O), 143.4, 137.7, 137.1, 129.9, 129.4, 129.2, 128.7, 128.4, 127.9, 125.8, 125.5, 46.2, 36.9, 21.1 (CH_3_); LC–MS: 304 (M − CF_3_COO^−^), 305 (M − CF_3_COO^−^ + H).

**General procedure for the synthesis of 3-amino-1,7-diphenyl-6,7-dihydro-5*H*-pyrrolo[1,2-*c*]imidazol-5-one (10a):** A mixture of the adduct **4a** (0.1 mmol) and 0.08 mL (0.11 mmol) of TFA was stirred in 2 mL of acetonitrile for 6 h, then conc. aqueous solution of NH_3_ was added to pH ≈ 8 and the solid product was filtered off, dried on air and crystallized from iPrOH. The title compound was obtained as a colourless solid (0.20 g, 68%); mp 198–200 °C; IR (KBr, cm^−1^) ν: 3486–3100 (NH_2_), 1784 (C=O); ^1^H NMR (200 MHz, DMSO-*d*_6_) δ 7.43–6.99 (m, 10H, H_arom_), 6.36 (br s, 2H, NH_2_), 4.75 (d, *J* = 2.9 Hz, 1H, CH_X_ ), 3.83, (dd, *J*_BX_ = 9.2 Hz, *J*_AB_ = 17.9 Hz, 1H, CH_B_), 2.87 (d, *J*_AX_ = 3.3 Hz, *J*_AB_ = 18.6 Hz, 1H, CH_A_); ^13^C NMR (125 MHz, DMSO-*d*_6_) δ 169.6 (C=O), 143.9, 142.5, 133.8, 129.7, 129.4, 128.5, 127.8, 127.6, 127.5, 126.5, 125.4, 47.6, 36.9; MS (*m*/*z*) (%): 289 (76) [M^+•^], 247 (53), 159 (38), 104 (29), 77 (100), 44 (41), 43 (19); anal. calcd for C_18_H_15_N_3_O (289.12) C, 74.74; H, 5.19; N, 14.53; found: C, 72.28; H, 6.79; N, 13.11.

**General procedure for the synthesis of 3-(2-amino-4-aryl-1*H*-imidazol-5-yl)-3-arylpropanoic acids:** A mixture of the corresponding adduct **4** (0.1 mmol) and 0.08 mL (0.11 mmol) of TFA was stirred in 2 mL of aqueous acetonitrile for 10–12 h. After cooling, the solid products **11** were filtered off, washed with iPrOH and dried on air. **11b**: pale yellow solid, 55%; mp 282–285 °C; ^1^H NMR (200 MHz, DMSO-*d*_6_) δ 7.38–7.23 (m, 4H, H_arom_), 7.22–6.98 (m, 5H, H_arom_), 5.72 (br s, 2H, NH_2_), 4.50–4.37 (m, 1H, CH_X_), 3.04–2.71 (m, 2H, H_A_H_B_), 2.21 (s, 3H, CH_3_); ^13^C NMR (125 MHz, DMSO-*d*_6_) δ 172.8 (COOH), 147.9, 136.6, 135.5, 134.5, 129.8, 129.4, 128.8, 128.1, 127.9, 127.8, 127.4, 36.7, 33.4, 21.0 (CH_3_); MS (*m*/*z*) (%): 321 (25) [M^+•^], 303 (25), 262 (100), 247 (15), 204 (10), 172 (11), 142 (20), 115 (22), 84 (22); anal. calcd for C_19_H_19_N_3_O_2_ (321.15) C, 71.01; H, 5.96; N, 13.08; found: C, 70.98; H, 6.06; N, 13.85.

**General procedure for the synthesis of 5-amino-3-(arylideneamino)-1,7-diaryl-7*H*-pyrrolo[1,2-*c*]imidazole-6-carbonitriles:** A mixture of the corresponding 2-amino-4-arylimidazole **1** (1.0 mmol), aromatic aldehyde **2** (2.0 mmol) and malononitrile **12** (1.0 mmol) in 2 mL of 2-propanol was refluxed during 20–30 min. After cooling, the yellow solid products **14** were filtered off and crystallized from iPrOH. **14a:** yellow powder, 65%; mp 221–222 °C; ^1^H NMR (200 MHz, DMSO-*d*_6_) δ 9.34 (s, 1H, CH*_azomethine_*), 8.18 (d, *J =* 7.3 Hz, 2H, Ar), 7.68–7.48 (m, 7H, Ar, C*^5^*NH_2_ ), 7.31–7.10 (m, 8H, Ar), 5.34 (s, 1H, C*^7^*H); ^13^C NMR (125 MHz, DMSO-*d*_6_) δ 162.4 (С*^3^*), 149.5 (C*_azomethine_*), 143.8 (C*^5^*), 138.3, 135.3, 133.3, 133.0, 132.5, 132.1, 130.4, 129.5, 129.4, 128.8, 128.2, 128.1, 127.4, 125.8, 117.9 (СN), 71.7 (C*^6^*), 45.01 (C*^7^*); MS (*m*/*z*) (%): 429 ([M^+•^], 25), 285 (100), 194 (19), 104 (26), 77 (19), 43 (25); anal. calcd for C_28_H_23_N_5_ (429.53) C, 78.30; H, 5.40; N, 16.31; found: C, 80.25; H, 5.70; N, 13.41.

**General procedure for the synthesis of 5-amino-1,7-diaryl-3-(arylideneamino)-7*Н*-pyrrolo[1,2-*с*]imidazole-6-carboxylates.** A mixture of the corresponding 2-amino-4-arylimidazole **1** (1.0 mmol), aromatic aldehyde **2** (2.0 mmol) and ethyl 2-cyanoacetate **15** (1.0 mmol) in 2 mL of 2-propanol was refluxed during 20–30 min. After cooling, the yellow solid products **16** were filtered off and crystallized from iPrOH. **16a**: yellow powder, 30%, mp 239–240 °C; ^1^H NMR (200 MHz, DMSO-*d*_6_) δ 9.32 (s, 1H, CH*_azomethine_*), 8.12 (d, *J =* 6.7 Hz, 2Н, Ar), 7.67–7.45 (m, 5Н, Ar), 7.27–7.04 (m, 10Н, C*^5^*NH_2_, Ar), 5.15 (s, *J =* 6.7 Hz, 1H, C*^7^*H), 4.05–3.84 (m, 2Н, О*СН*_2_СН_3_), 1.01 (s, *J* = 7.0, 3H, ОСН_2_*CH*_3_); ^13^C NMR (125 MHz, DMSO-*d*_6_) δ 178.7 (CO), 134.5, 133.3, 130.2, 129.6, 128.7, 128.6, 128.3, 127.8, 127.2, 127.0, 126.4, 125.8, 125.5, 116.7, 93.4, 58.9, 43.4, 14.7; MS (*m*/*z*) (%): 448 ([M^+•^], 100; anal. calcd. for C_28_H_24_N_4_O_2_ (448.19) C, 74.98; H, 5.39; N, 12.49; found: С, 75.12; H, 4.89; N, 11.37.

**General procedure for the synthesis of 3',5'-diamino-1-alkyl-2-oxo-1'-arylspiro[indolin-3,7'-pyrrolo[1,2-*c*]imidazole]-6'-carbonitriles:** The mixture of corresponding 2-amino-4-arylimidazoles **1** (1.0 mmol), isatin **18** (1.0 mmol) and malononitrile **12** (1.0 mmol) in 2 mL of 2-propanol was refluxed during 50–60 min. After cooling, the solid products **19** were filtered off and crystallized from iPrOH. **19a**: colourless solid, 60%, mp 250–252 °C, ^1^H NMR (200 MHz, DMSO-*d*_6_) δ 7.77 (br s, 2H, С*^5’^*NH_2_), 7.37 (t, *J* = 7.9 Hz, 1H, Ar*_isatin_*), 7.24–7.10 (m, 2H, Ar), 7.10–6.95 (m, 4H, Ar), 6.94–6.82 (m, 2H, Ar), 6.47 (br s, 2H, С*^3’^*NH_2imidazole_), 3.21 (s, 3H, N*^1^*CH_3_); ^13^C NMR (125 MHz, DMSO-*d*_6_) δ 176.3 (C*^2^*), 154.2 (C*^5’^*), 146.4 (С*^3’^*), 145.7, 135.4, 133.0, 132.2, 130.7, 130.3, 129.0, 127.2, 126.6, 126.1, 126.0, 111.7, 69.8 (C*^6’^*), 55.8 (С*_spiro_*), 29.2 (N*^1^*СН_3_); MS (*m*/*z*) (%): 369 [M + H]^+^ (100); anal. calcd for C_21_H_16_N_6_O (368.14) C, 68.47; H, 4.38; N, 22.81; found: С, 69.43; H, 5.07; N, 22.64.

**General procedure for the synthesis of 3',5'-diamino-1-alkyl-2-oxo-1'-arylspiro[indoline-3,7'-pyrrolo[1,2-*c*]imidazole]-6'-carboxylates:** The mixture of corresponding 2-amino-4-arylimidazoles **1** (1.0 mmol), isatin **18** (1.0 mmol) and ethyl 2-cyanoacetate **15** (1.0 mmol) in 2 mL of 2-propanol was refluxed during 50–60 min. After cooling, the solid products **20** were filtered off and crystallized from iPrOH. **20a**: colourless solid, 72%, mp 280–282 °C; ^1^H NMR (200 MHz, DMSO-*d*_6_) δ 7.63 (br s, 2H, С*^5’^*NH_2_), 7.29 (t, *J* = 7.5 Hz, 1H, Ar), 7.11–6.88 (m, 8Н, Ar), 6.46 (br s, 2H, С*^3’^*NH_2_), 3.83–3.63 (m, 2Н, СО*СН_2_*СН_3_), 3.21 (s, 3Н, N*^1^*СН3), 0.88–0.69 (m, 3Н, СОСН_2_*СН_3_*); ^13^C NMR (125 MHz, DMSO-*d*_6_) δ 175.0 (C*^2^*), 145.0 (C*^5’^*), 143.7 (С*^3’^*), 133.6, 130.4, 130.2, 129.0, 128.5, 126.7, 125.3, 125.0, 123.5, 123.1, 108.6, 58.5 (C*^6’^*), 52.7 (С*_spiro_*), 33.4 (СО*СН_2_*СН_3_), 26.9 (N*^1^*СН_3_), 14.3 (СОСН_2_*СН_3_*); MS (*m*/*z*) (%): 416 [M + H]^+^ (100); anal. calcd for C_23_H_21_N_5_O_3_ (415.16) C, 66.49; H, 5.09; N, 16.86; found: С, 67.89; H, 5.64; N, 11.70.

### Experimental part of X-ray diffraction study

The crystals of **9i** (C_24_H_20_N_3_O^+^, C_2_F_3_O_2_^−^) are triclinic. At 293 K *a* = 8.4770(6), *b* = 11.317(1), *c* = 13.027(1) Å, α = 69.101(9)°, b = 77.989(8)°, γ = 87.527(7)°, *V* = 1141.3(2) Å^3^, *M*_r_ = 479.45, *Z* = 2, space group 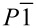, *d*_calc_ = 1.395 g/cm^3^, μ(Mo Ka) = 0.109 mm^−1^, F(000) = 496. Intensities of 8769 reflections (3910 independent, *R*_int_ = 0.027) were measured on the «Xcalibur-3» diffractometer (graphite monochromated Mo Kα radiation, CCD detector, ω-scaning, 2Θ_max_ = 50°).

The crystals of **11b** (C_19_H_19_N_3_O_2_·H_2_O) are monoclinic. At 293 K *a* = 16.4288(9), *b* = 9.3556(4), *c* = 12.1174(8) Å, β = 110.151(7)°, *V* = 1748.5(2) Å^3^, *M*_r_ = 339.39, *Z* = 4, space group *P*21/*c*, *d*_calc_ = 1.289 g/cm^3^, μ(Mo Ka) = 0.089 mm^−1^, F(000) = 720. Intensities of 16955 reflections (5089 independent, *R*_int_ = 0.060) were measured on the«Xcalibur-3» diffractometer (graphite monochromated Mo Kα radiation, CCD detector, ω-scaning, 2Θ_max_ = 60°).

The crystals of **16a** (C_28_H_24_N_4_O_2_) are triclinic. At 293 K *a* = 8.322(3) Å, *b* = 9.563(6) Å, *c* = 16.053(5) Å, α = 94.08(4)°, β = 101.46(3)°, γ = 109.97(4)°, *V* = 1163.3(10) Å^3^, *M*_r_ = 448.53, *Z* = 2, space group 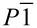, *d*_calc_ = 1.2804 g/cm^3^, μ(Mo Ka) = 0.083 mm^−1^, F(000) = 472. Intensities of 12048 reflections (3968 independent, *R*_int_ = 0.167) were measured on the «Xcalibur-3» diffractometer (graphite monochromated Mo Kα radiation, CCD detector, ω-scaning, 2Θ_max_ = 50°).

The crystals of **19a** (C_24_H_25_N_7_O_3_) are triclinic. At 293 K *a* = 7.9380(5) Å, *b* = 8.4953(5) Å, *c* = 17.6908(9) Å, α = 98.891(4)°, β = 101.017(5)°, γ = 91.630(5)°, *V* = 1154.86(12) Å^3^, *M*_r_ = 459.51, *Z* = 2, space group 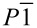, *d*_calc_ = 1.321 g/cm^3^, μ(Mo Ka) = 0.091 mm^−1^, F(000) = 484. Intensities of 11884 reflections (6633 independent, *R*_int_ = 0.0265) were measured on the «Xcalibur-3» diffractometer (graphite monochromated Mo Kα radiation, CCD detector, ω-scaning, 2Θ_max_ = 50°).

The structures were solved by direct methods using the SHELXTL package [41]. The position of the hydrogen atoms were located from electron density difference maps and refined by the “riding” model with U_iso_ = *n*U_eq_ of the carrier atom (*n* = 1.5 for methyl and hydroxy groups and for water molecules and *n* = 1.2 for other hydrogen atoms) in the structures **11b** and **16a**. The hydrogen atoms of the compounds **11b** and **19a** which take part in the formation of the hydrogen bonds were refined using the isotropic approximation as well as all hydrogen atoms in the structure **9i**. Full-matrix least-squares refinement of the structures against F2 in anisotropic approximation for non-hydrogen atoms using 3879 (**9i**), 5051 (**11b**), 3968 (**16a**) and 6633 (**19a**) reflections was converged to: w*R*_2_ = 0.052 (*R*_1_ = 0.031 for 1903 reflections with F>4σ(F), S = 0.964) for structure **9i**, w*R*_2_ = 0.117 (*R*_1_ = 0.054 for 2480 reflections with F>4σ(F), S = 0.992) for structure **11b,** w*R*_2_ = 0.107 (*R*_1_ = 0.079 for 942 reflections with F>4σ(F), S = 0.881) for structure **16a** and w*R*_2_ = 0.147 (*R*_1_ = 0.065 for 3693 reflections with F>4σ(F), S = 1.045) for structure **19a**. The final atomic coordinates, and crystallographic data for molecules **9i** and **11b** have been deposited to with the Cambridge Crystallographic Data Centre, 12 Union Road, CB2 1EZ, UK (fax: +44-1223-336033; e-mail: deposit@ccdc.cam.ac.uk) and are available on request quoting the deposition numbers CCDC 1855490 for **9i**, CCDC 1855491 for **11b,** CCDC 1895778 for **16a** and CCDC 1895793 for **19a**).

## Supporting Information

File 1 Experimental and analytical data, X-ray diffraction studies and NMR spectra.
