# Supplementary material for: Novel (2-amino-4-arylimidazolyl)propanoic acids and pyrrolo[1,2-c]imidazoles via the domino reactions of 2-amino-4-arylimidazoles with carbonyl and methylene active compounds
Source: Beilstein J Org Chem. 2019 May 6;15:1032–45. doi: 10.3762/bjoc.15.101 (PMC6541323; doi:10.3762/bjoc.15.101)
Supplement: File 1 — Experimental and analytical data, X-ray diffraction studies and NMR spectra. [file Beilstein_J_Org_Chem-15-1032-s001.pdf]

BEILSTEIN JOURNAL OF ORGANIC CHEMISTRY

Supporting Information

for

Novel (2-amino-4-arylimidazolyl)propanoic acids and pyrrolo[1,2-*c*]imidazoles via the domino reactions of 2-amino-4-arylimidazoles with carbonyl and methylene active compounds

Victoria V. Lipson, Tetiana L. Pavlovska, Nataliya V. Svetlichnaya, Anna A. Poryvai, Nikolay Yu. Gorobets, Erik V. Van der Eycken, Irina S. Konovalova, Svetlana V. Shiskina, Alexander V. Borisov, Vladimir I. Musatov and Alexander V. Mazepa

Beilstein J. Org. Chem. **2019,** *15,* 1032–1045. doi:10.3762/bjoc.15.101

Experimental and analytical data, X-ray diffraction studies and NMR spectra

License and Terms: This is a supporting information file under the terms of the Creative Commons Attribution License (http://creativecommons.org/licenses/by/4.0). Please note that the reuse, redistribution and reproduction in particular requires that the authors and source are credited.
The license is subject to the *Beilstein Journal of Organic Chemistry* terms and conditions: (https://www.beilstein-journals.org/bjoc)

Table of contents

General methods **S2**

X-ray diffraction studies **S2**

Synthesis of 5-((2-amino-4-aryl-1*H*-imidazol-5-yl)(aryl)methyl)-6-hydroxy-2,2-dimethyl-4*H*-1,3-dioxin-4-ones 4a-i **S6**

Synthesis of 5-oxo-1,7-diaryl-6,7-dihydro-5*H*-pyrrolo[1,2-*c*]imidazol-3-aminium 2,2,2-trifluoroacetates 9 b,c,g **S14**

Synthesis of 3-amino-1,7-diphenyl-6,7-dihydro-5*H*-pyrrolo[1,2-*c*]imidazol-5-one (10a) **S17**

Synthesis of 3-(2-amino-4-aryl-1*H*-imidazol-5-yl)-3-arylpropanoic acids 11b–f,h **S18**

Synthesis of 5-amino-3-(arylideneamino)-1,7-diaryl-7*H*-pyrrolo[1,2-c]imidazole-6-carbonitriles 14a–f **S24**

Synthesis of 5-amino-1,7-diaryl-3-(arylideneamino)-7*H*-pyrrolo[1,2-*c*]imidazole-6-carboxylates 16a,b **S30**

Synthesis of 3',5'-diamino-1-alkyl-2-oxo-1'-arylspiro[indolin-3,7'-pyrrolo[1,2-*c*]imidazole]-6'-carbonitriles 19a–h **S32**

Synthesis of 3',5'-diamino-1-alkyl-2-oxo-1'-arylspiro[indoline-3,7'-pyrrolo[1,2-*c*]imidazole]-6'-carboxylates 20a–c **S40**

General methods

Reagents and analytics: Starting materials were purchased from commercial suppliers. Melting points were determined on a *Kofler apparatus* and temperatures were not corrected. The IR spectra were recorded in KBr on a Specord M-82 spectrometer. The ^{1}H NMR spectra were registered on a *Varian Mercury VX-200* (200 MHz) and *Bruker AM-400 spectrometer* (400 MHz), ^{13}C NMR spectra were registered on a Bruker AM-*400* (100 MHz) and *Bruker Avance DRX 500* (125 MHz) spectrometers in DMSO-d_6, $CDCl_3$ and trifluoroacetic acid (TFA) using TMS as internal standard. The mass spectra were recorded on a Varian 1200L GC–MS instrument, ionization by EI at 70 eV. Fast atom bombardment (FAB) mass spectrometry was performed on a *VG 70-70EQ* mass spectrometer, equipped with an argon primary atom beam, and an *m*-nitrobenzyl alcohol matrix was utilized. LC–MS experiments were performed on an *Applied Biosystems* (*Shimadzu 10-AV LC*, *Gilson-215* automatic giving, massspectrometer *API 150EX*, detectors UV (215 and 254 nm), and *ELS*, column *Luna-C18, Phenomenex*, 5 µ, 100 Angstrom, 150 × 2 mm RP). Elemental analyses were made on an elemental analyzer *Euro AE-3000*. The progress of reactions and also the purity of the obtained compounds were monitored by TLC on Silufol UV-254 plates in EtOAc/CH2Cl2 (1:4) and visualized under UV light or iodine fume.

X-ray diffraction studies

Molecular and crystal structure of the compound 9i

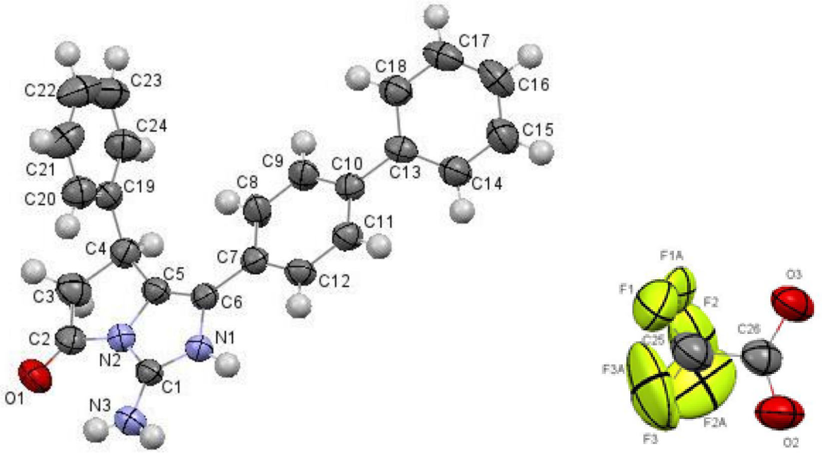

Figure S1. The molecular structure of the compound **9i** according to X-ray diffraction data. Thermal ellipsoids of atoms are shown at 50% probability levels.

The compound **9i** exists as organic salt with trifluoroacetic acid in the crystal phase. The existence of the trifluoroacetic molecule as anion is confirmed by similar values of the C–O bond lengths (1.229(2) Å and 1.238(2) Å, respectively) and the absence of the hydrogen atom at the carboxylic group. The analysis of the bond lengths in the imidazole ring has revealed that the C1–N1 and C1–N3 bonds are equal (1.320(3) Å and 1.320(2) Å, respectively) and the N1–C6 bond (1.414(6) Å) is slightly elongated as compare to its mean value 1.376 Å [1]. The hydrogen atoms at the N1 and N3 were located from the electron density difference maps. As a result we may describe the structure of the organic cation as superposition of two forms:

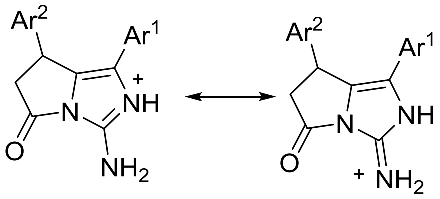

Scheme S1. Two forms of cation **9i**.

Dihydropyrrole ring of the bicyclic fragment adopts an envelope conformation with deviation of the C4 atom by 0.19 Å from the mean plane of the remaining atoms of this ring. The phenyl substituent has axial orientation and is turned relatively the C5–C4 bond (the N2–C5–C4–C19 and C5–C4–C19–C20 torsion angles are 112.4(1)° and −51.7(2)°, respectively). Two aromatic rings of the biphenyl substituent is turned relatively each other (the C9–C10–C13–C18 torsion angle is 33.8(2)°) due to repulsion between them (the shortened intramolecular contacts H18···C9 2.66 Å (the van der Waals radii sum [2] is 2.87 Å), H18···H9 2.27 Å (2.34 Å), H9···C18 2.76 Å (2.87 Å), H11···C14 2.77 Å (2.87 Å), H14···C11 2.80 Å (2.87 Å)). Meanwhile the C7···C12 aromatic ring of this substituent is coplanar to the imidazole ring (the C8–C7–C6–C5 torsion angle is −1.8(3)°) in spite the presence of the shortened intramolecular contacts H8···C4 2.72 Å (2.87 Å), H8···C5 2.72 Å (2.87 Å), H1N···H12 2.19 Å (2.34 Å), H1N···C12 2.79 Å (2.87 Å), H12···N1 2.64 Å (2.67 Å).

In the crystal phase the cation and anion are bonded by intermolecular hydrogen bonds N3–H3Na···O2’ (2-x, 1-y, 1-z) H···O 2.03 Å N–H···O 171° and N1–H1Na···O3’ (2-x, 1-y, 1-z) H···O 1.74 Å N–H···O 174°.

Molecular and crystal structure of the compound 11b

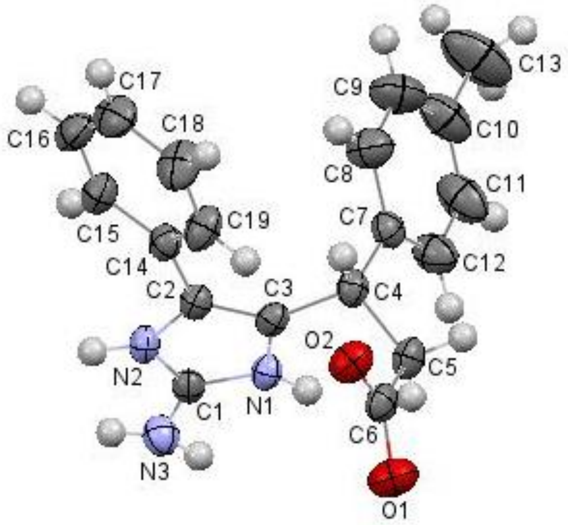

Figure S2. The molecular structure of the compound **11b** according to X-ray diffraction data. Thermal ellipsoids of atoms are shown at 50% probability levels.

The compound **11b** was found to be a zwitterion and exist as monohydrate in the crystal phase. The absence of the hydrogen atom and equalization of the C6–O1 and C6–O2 bond lengths (1.254(2) Å and 1.259(2) Å respectively) allow to presume the location of the negative charge at the carboxylic group. The very close lengths of the bonds centered at the C1 atom (the N2–C1 bond length is 1.332(2) Å, the C1–N3 bond length is 1.337(3) Å and the N1–C1 bond length is 1.340(2) Å) allows to describe the zwitterion as superposition of three forms with different location of the positive charge:

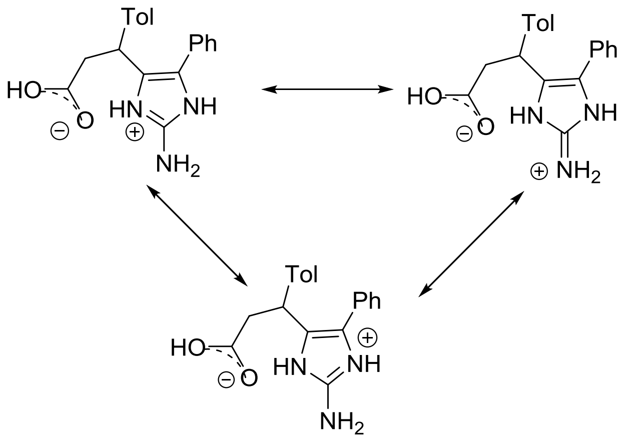

Scheme S2. Three forms of the compound **11b** in the crystal phase.

The phenyl substituent at the heterocycle is turned relatively the C2–C3 endocyclic double bond (the C19–C14–C2–C3 torsion angle is −41.1(3)°) due to steric repulsion between vicinal substituents (the shortened intramolecular contacts are: H4···C19 2.67 Å (2.87 Å), H4···H19 2.22 Å (2.34 Å), C4···C19 3.39 Å (3.42 Å). The substituent at the C3 atom is located in such a way that the C2–C3–C4–H4 torsion angle is 10.4(2)°. The *p*-tolyl group of this substituent is turned orthogonally to the C3–C4 bond (the C3–C4–C7–C8 torsion angle is 88.9(2)°). The deprotonated carboxyl group is located in *–sc* conformation relatively the C3–C4 bond and is turned to the C4–C5 bond (the C3–C4–C5–C6 and C4–C5–C6–O2 torsion angles are −68.9(2)° and −36.9(2)° respectively).

In the crystal phase molecules **11b** form zigzag chains along [001] crystallographic direction due to the N1–H1N···O2' and N3–H3Na···O1' (x, 1.5-y, -0.5+z) intermolecular hydrogen bonds (H1···O2 1.89 Å N1–H···O2 174°, H3···O1 2.09 Å N3–H···O1 173°). The neighboring chains are bound by the N3–H3Nb···O1' (1-x, 1-y, 1-z) hydrogen bond (H···O 2.32 Å N–H···O 159°) and through the bridged water molecules due to formation of the following intermolecular hydrogen bonds:

N2–H···O1w' H···O 1.84 Å N–H···O 170°;

O1w–H1wa···O2' (1-x, -0.5+y, 1.5-z) H···O 1.86 Å O–H···O 161°;

O1w–H1wb···O1' (x, y-1, z) H···O 1.91 Å O–H···O 165°.

Molecular and crystal structure of the compound 16a

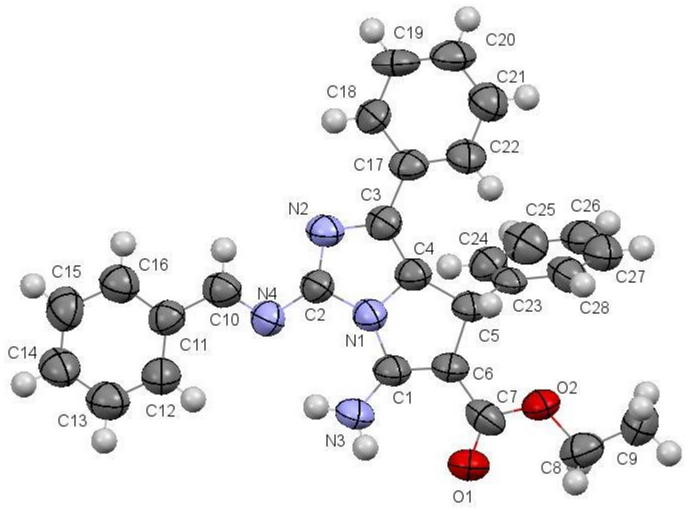

Figure S3. The molecular structure of the compound **16a** according to X-ray diffraction data. Thermal ellipsoids of atoms are shown at 50% probability levels.

All atoms of the bicyclic fragment lie in the plane within 0.01 Å. The analysis of the bond lengths has shown that the formally single exocyclic C1–N3 bond is shorter than the double endocyclic C6–C1 bond (1.336(6) Å and 1.354(9) Å respectively). At that C1 and C6 atoms are planar indicating their sp^2-hybridization. Such a distribution of electron density allows to discuss the zwitter-ionic form contribution and consider the structure of **16a** as superposition of two resonance structures:

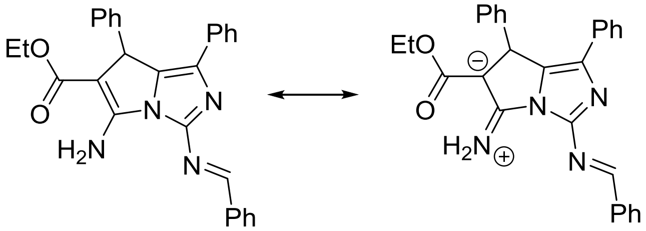

Scheme 3. Resonance structures of **16a**.

The phenyl substituent at the C5 atom of the bicyclic fragment has equatorial orientation and is turned relatively to the C4–C5 bond (the N1–C4–C5–C23 and C4–C5–C23–C24 torsion angles are 123.4(1)° and −43.0(2)°, respectively). The phenyl substituent at the C3 atom is slightly turned relatively the N2–C3 endocyclic bond (the N2–C3–C17–C18 torsion angle is −8.21(4)°) despite the steric repulsion between atoms of bicycle and hydrogen atoms of phenyl substituent (the shortened intramolecular contacts are: N2···H18 2.56 Å (2.66 Å), C4···H22 2.80 Å (2.87 Å)). The phenyl group of the substituent at the C2 atom is located in *ap*-conformation relatively to the C2–N4 bond and is almost coplanar to the N4–C10 bond (the C2–N4–C10–C11 and N4–C10–C11–C12 torsion angles are 179.6(2)° and 2.2(2)° respectively). Such orientation is stabilized by the formation of the N3–H3b···N4 intramolecular hydrogen bond (H···N 2.42 Å, N–H···N 117°). The substituent at the C6 atom is orientated in the same way as the substituent at the C2 atom (the C6–C7–O2–C8 and C7–O2–C8–C9 torsion angles are −175.1(1)° and 178.3(2)° respectively) probably due to the formation of the weak N3–H3a···O1 intramolecular hydrogen bond (H···O 2.45 Å, N–H···O 107°).

In the crystal phase molecules **16a** form centrosymmetric dimers bound by the N3–H3a···O1’ (2-x, 2-y,1-z) intermolecular hydrogen bond (H···O 2.18 Å, N–H···O 129º).

Molecular and crystal structure of the compound 19a

The compound **19a** exist as solvate with dimethylformamide and water molecule in ratio 1:1:1 in the crystal phase.

The spiro-joined bicyclic fragments are turned relatively to each other in such a way that the dihedral angle between mean planes of the bicycles is 84.5°. The analysis of the bond lengths has shown that the formally single exocyclic C6–N6 bond is significantly shorter than the double endocyclic C6–C5 bond (1.319(2) Å and 1.373(3) Å respectively). At that C1 and C6 atoms are planar indicating their sp^2-hybridization. Such a distribution of electron density allows to discuss the zwitter-ionic form contribution and consider the structure of **19a** as superposition of two resonance structures similar to **16a**:

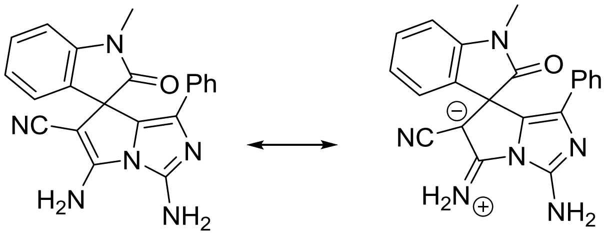

Scheme S4. Resonance structures of **19a**.

The phenyl substituent is turned relatively the C2–C3 endocyclic bond (the C3–C2–C16–C21 torsion angle is −30.4(3)°) due to steric repulsion between atoms of bicycle and hydrogen

atoms of phenyl substituent (the shortened intramolecular contact is: N2···H17 2.64 Å (2.67 Å)) on the one hand and by the formation of the C21–H21···C9 (π) intramolecular hydrogen bond (H···C 2.61 Å, C–H···C 148°), on the other hand. The N(5) atom of the aminogroup has pyramidal configuration while the N(6) atom is almost planar (the sums of bond angles centered at the N(5) and N(6) atoms are 348.2º and 356.7º respectively). Such configuration of aminogroups observed in the crystal despite of the steric repulsion between hydrogen atoms of aminogroups (the shortened intramolecular contact is: H5b···H6a 2.13 Å (2.34 Å)).

In the crystal the molecules form the centrosymmetric dimers by N5–H5NB...N2′ (-x+1,-y+1,-z) (H···N 2.13 Å, N–H···N 169°) intermolecular hydrogen bonds. Each monomer of such dimer is bonded with DMF and water solvate molecule by the N6–H6NA...O1W′ (x+1,y,z) (H···O 1.97 Å, N–H···O 172°) and N6–H6NB...O1S′ (x+1,y,z) (H···O 2.03 Å, N–H···O 164°) hydrogen bonds.

Synthesis of 5-((2-amino-4-aryl-1*H*-imidazol-5-yl)(aryl)methyl)-6-hydroxy-2,2-dimethyl-4*H*-1,3-dioxin-4-ones 4a–i

General procedure. An equimolar mixture (1.0 mmol) of the corresponding 2-amino-4-arylimidazole **1**, aromatic aldehyde **2** and Meldrum's acid **3** was refluxed in iPrOH (3 mL) for 3-5 min. After cooling, the solid product was filtered off, washed with iPrOH and dried on air.

5-((2-Amino-4-phenyl-1H-imidazol-5-yl)(phenyl)methyl)-6-hydroxy-2,2-dimethyl-4H-1,3-dioxin-4-one (4a)

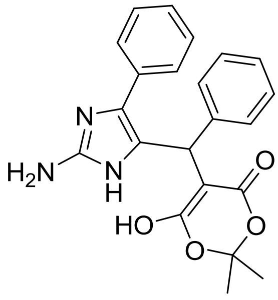

Colorless solid, 77%, mp 243-245 C; IR (KBr, cm^{-1}) ν 3404-2800 (NH_2, NH, OH), 1684 (C=O); ^1H NMR (200 MHz, DMSO-d_6): δ=12.27 (br s, 2H, NH, OH), 7.61-7.49 (m, 2H, H_{arom}), 7.48-7.31 (m, 5H, H_{arom}), 7.27-7.01 (m, 5H, NH_2,, H_{arom}), 5.48 (s, 1H, CH), 1.51 (s, 6H, CH_3); ^{13}C NMR (125 MHz, $CDCl_3$): δ=166.8 (C=O), 146.6 (C-OH), 144.0, 129.4, 128.9, 128.4, 128.3, 127.5, 127.4, 127.2, 125.9, 121.1, 100.6, 76.0 (C=COH), 35.1 (CH), 26.4 (CH_3), 25.9 (CH_3); *m/z* (EI, 70 eV): 289 (76), (391 [$M^{+\bullet}$]-44-58), 247 (53), 159 (36), 104 (19), 77 (8), 44 (39), 43 (100). Anal. calcd. for $C_{22}H_{21}N_3O_4$ (391.5) C, 67.52; H, 5.37; N, 10.74%; Found: C, 68.77; H, 5.93; N, 10.79.

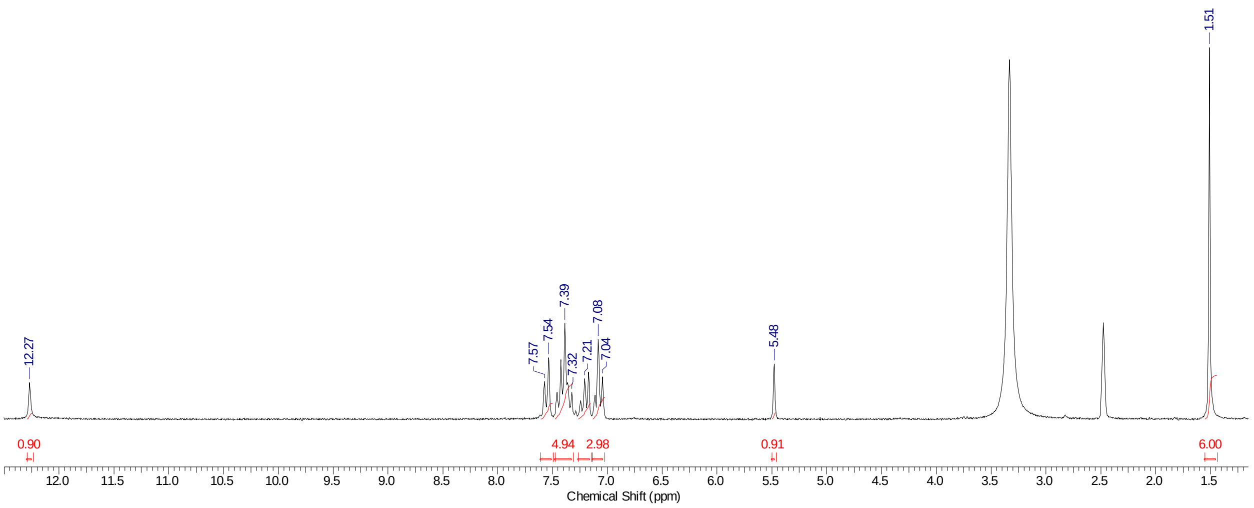

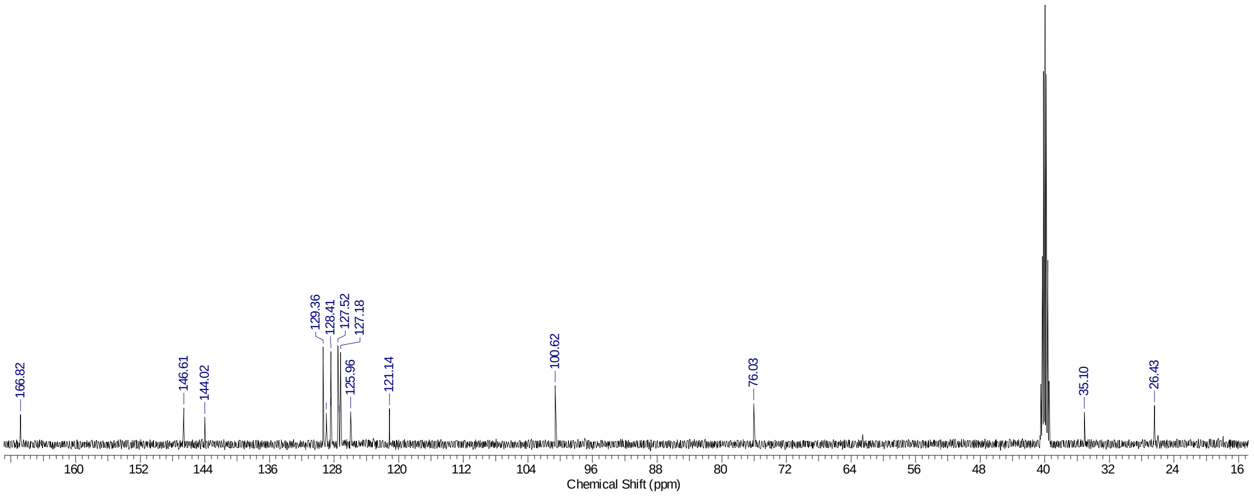

5-((2-Amino-4-phenyl-1H-imidazol-5-yl)(p-tolyl)methyl)-6-hydroxy-2,2-dimethyl-4H-1,3-dioxin-4-one (4b)

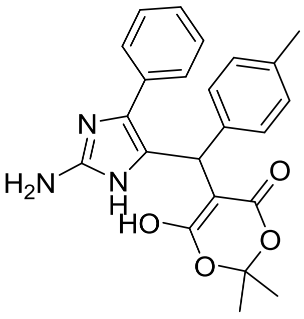

Colorless solid, 30%, mp 208-210 C; IR (KBr, cm^{-1}) ν 3408-2764 (NH_2, NH, OH), 1684 (C=O); ^{1}H NMR (200 MHz, DMSO-d_6): δ=11.37 (br s, 2H, NH, OH), 7.51-7.12 (m, 5H, H_{arom}), 7.03-6.81 (m, 4H, H_{arom}), 6.43 (br s, 2H, NH_2), 5.92 (s, 1H, CH), 2.17 (s, 3H, CH_{3arom}), 0.94 (s, 6H, CH_3); ^{13}C NMR (125 MHz, $CDCl_3$): δ=166.7 (C=O), 147.4 (C-OH), 145.2, 129.6, 128.2, 127.4, 127.0, 126.4, 125.6, 100.3, 76.6 ($\underline{C}$=COH), 35.5 (CH), 26.5 (CH_3), 21.2 (CH_3); *m/z* (EI, 70 eV) 303 (79) (405 $[M^{+\bullet}]$-44-58), 246 (100), 173 (34)/ 129 (17), 104 (44). Anal. calcd. for $C_{23}H_{23}N_3O_4$ (405.45) C, 68.15; H, 5.68; N, 10.37; Found,%: C, 68.70; H, 5.94; N, 10.79.

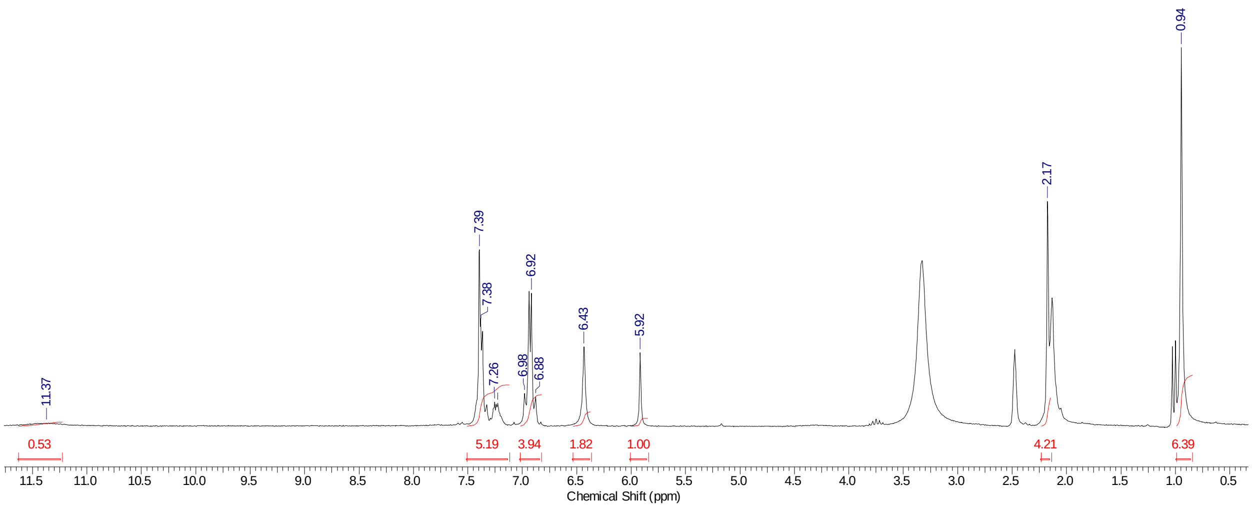

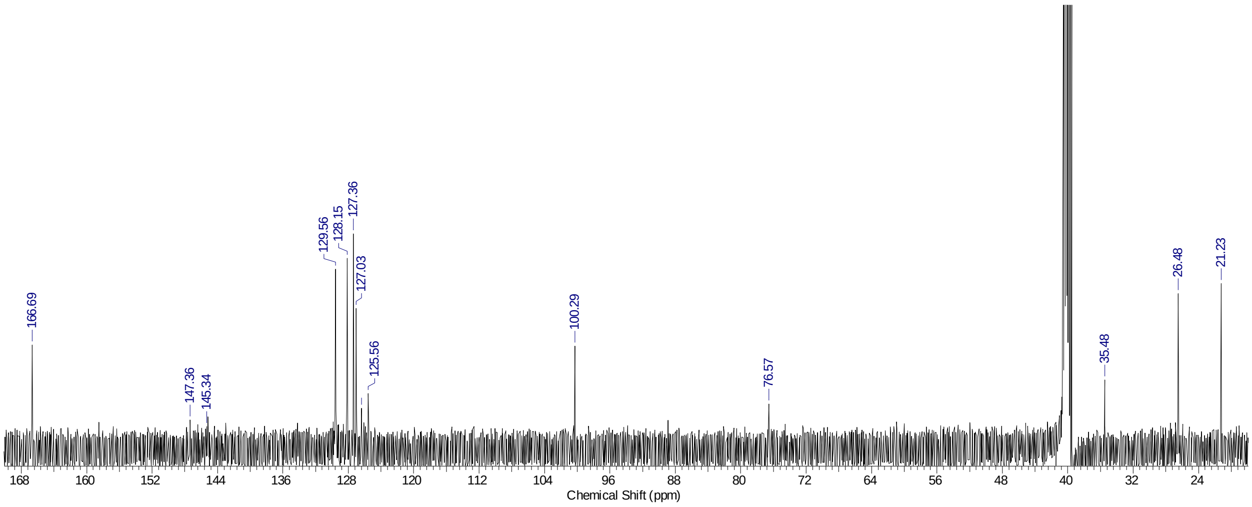

5-((2-Amino-4-(p-tolyl)-1H-imidazol-5-yl)(phenyl)methyl)-6-hydroxy-2,2-dimethyl-4H-1,3-dioxin-4-one (4c)

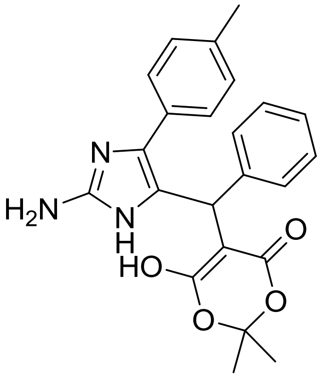

Colorless solid, 49%, mp 240-242 C; IR (KBr, cm^{-1}) ν 3428-2750 (NH_2, NH, OH), 1684 (C=O); ^1H NMR (200 MHz, DMSO-d_6) δ=11.06 (br s, 1H, NH, OH), 7.46 (m, 2H, H_{arom}), 7.33-7.05 (m, 7H, H_{arom}), 6.31 (br s, 2H, NH_2), 5.53 (s, 1H, CH), 2.24 (s, 3H, CH_{3arom}), 1.48 (s, 6H, CH_3); ^{13}C NMR (125 MHz, $CDCl_3$): δ=166.7 (C=O), 147.4 (C-OH), 145.2, 129.6, 128.2, 127.4, 127.0, 126.4, 125.6, 100.3, 76.6 ($\underline{C}$=COH), 35.5 (CH), 26.5 (CH_3), 21.2 (CH_3); *m/z* (EI, 70 eV): 303 (100) (405 [$M^{+\bullet}$]-44-58), 246 (12), 204 (10), 144 (15), 102 (13), 91 (11), 77 (10). Anal. calcd. for $C_{23}H_{23}N_3O_4$ (405.45) C, 68.15; H, 5.68; N, 10.37; Found,%: C, 68.55; H, 5.71; N, 10.48.

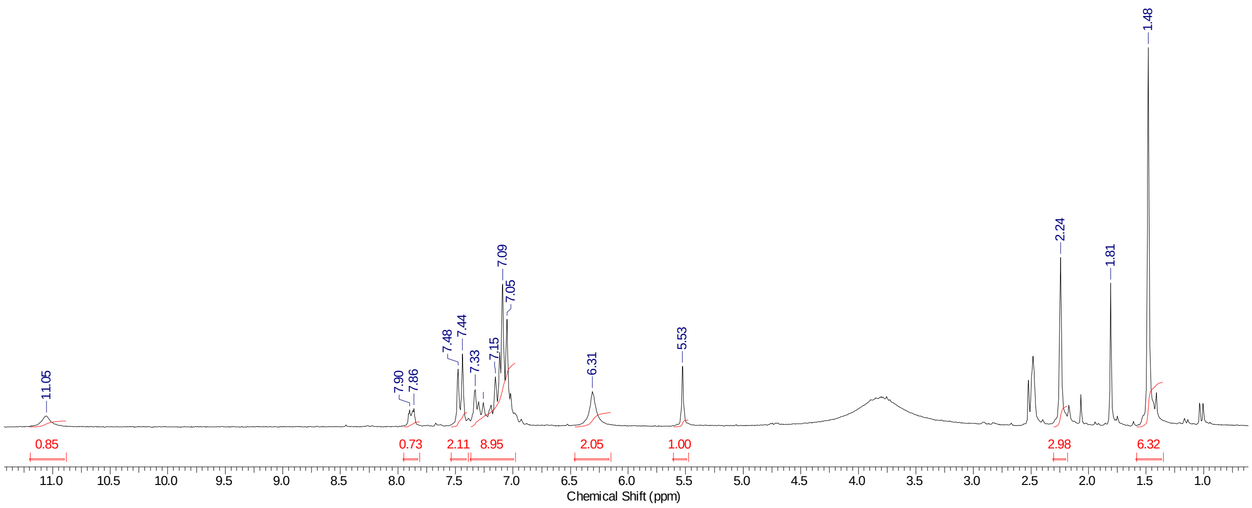

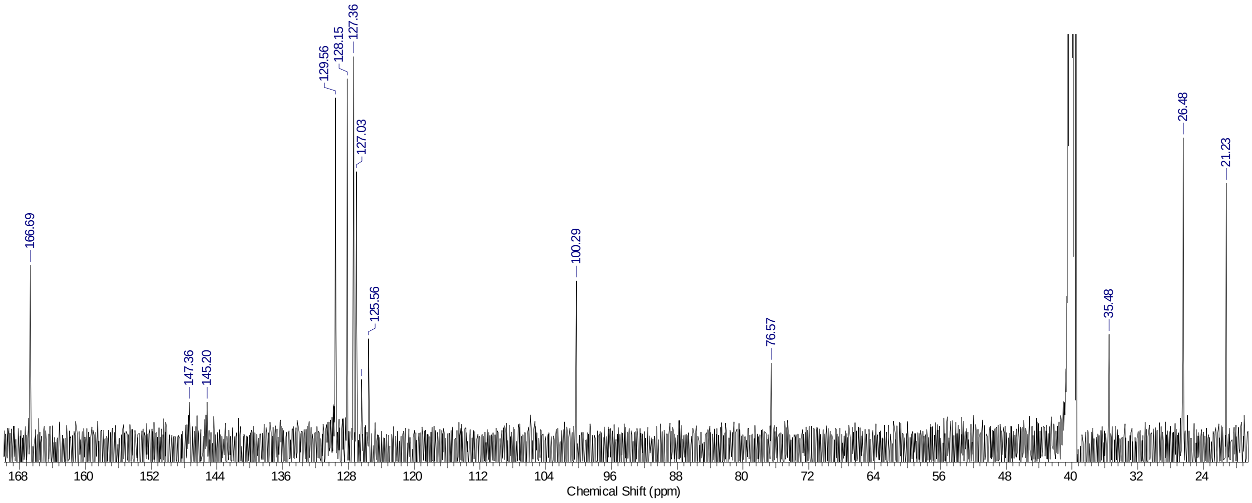

5-((2-Amino-4-(p-tolyl)-1H-imidazol-5-yl)(p-tolyl)methyl)-6-hydroxy-2,2-dimethyl-4H-1,3-dioxin-4-one (4d)

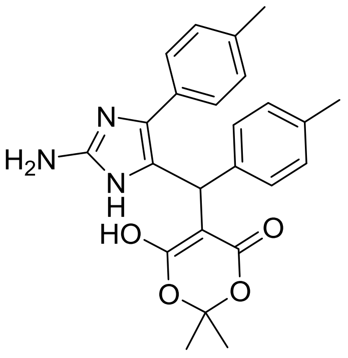

Pale yellow solid, 50%, mp 239-241 C; ^{1}H NMR (200 MHz, DMSO-d_6) δ=12.28 (br s, 1H, NH), 12.01 (br s, 1H, OH),), 7.40 (d, 2H, *J* 8 Hz, 2,6-H_{arom}), 7.34 (br s, 2H, NH_2), 7.21 (d, 2H, *J* 8 Hz, 3,5-H_{arom}), 6.99 (d, 2H, *J* 8 Hz, 2,6-H_{arom}), 6.94 (d, 2H, *J* 8 Hz, 3,5-H_{arom}), 5.41 (s, 1H, CH), 2.28 (s, 3H, CH_{3arom}), 2.19 (s, 3H, CH_{3arom}), 1.49 (s, 6H, CH_3); ^{13}C NMR (125 MHz, DMSO-d_6) δ=166.8 (C=O), 146.4 (C-OH), 141.2, 137.8, 134.8, 129.9, 128.97, 127.4, 127.1, 126.2, 120.98, 100.5, 76.3 (<u>C</u>=COH), 62.5 (CH_3), 34.8 (CH), 26.4 (CH_3), 26.0 (CH_3), 21.3 (CH_3); LC-MS: 419 (M), 418 (M-H), 420 (M+H). Anal. calcd. for $C_{24}H_{25}N_3O_4$ (419.18) C, 68.72; H, 6.01; N, 10.02; Found,%: C, 70.02; H, 6.14; N, 10.52.

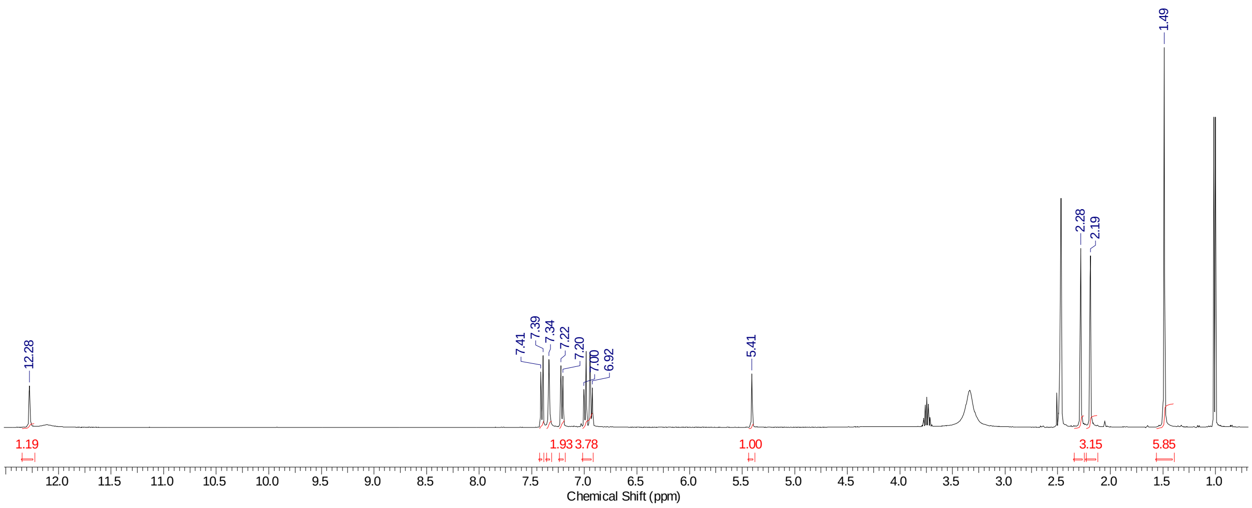

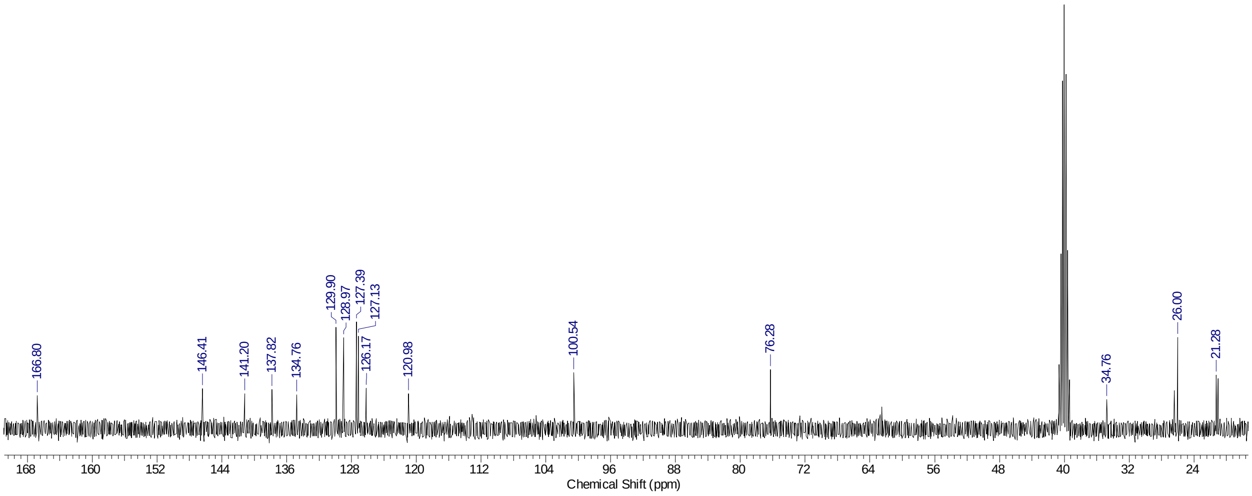

5-((2-Amino-4-(p-tolyl)-1H-imidazol-5-yl)(4-nitrophenyl)methyl)-6-hydroxy-2,2-dimethyl-4H-1,3-dioxin-4-one (4e)

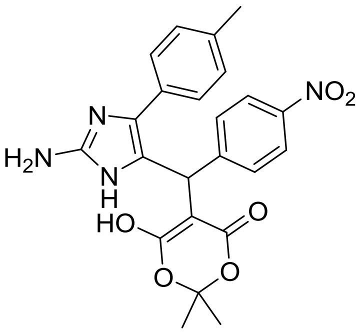

Pale yellow solid, 40%, mp 259-260 C; ^{1}H NMR (400 MHz, DMSO-d_6) δ=12.26 (br s, 1H, OH), 11.92 (s, 1H, NH), 8.09 (d, 4H, *J* 7.1 Hz, H_{arom}), 7.41 (br s, 2H, NH_2), 7.37 (d, 2H, *J* 6.8 Hz, H_{arom}), 7.32 (d, 2H, *J* 7.6 Hz, H_{arom}), 7.22 (d, 2H, *J* 7.1 Hz, H_{arom}), 5.50 (s, 1H, CH), 2.27 (s, 3H, CH_{3arom}), 1.49 (s, 6H, CH_3); ^{13}C NMR (125 MHz, DMSO-d_6) δ=166.6 (C=O), 152.7 (C-OH), 146.8, 146.0, 138.2, 129.9, 128.4, 127.5, 125.8, 125.3, 123.7, 121.8, 100.8, 76.1 (CH_3), 35.6 (CH), 26.4 (CH_3), 21.3(CH_3); LC-MS: 450 (M), 449 (M-H), 451 (M+H). Anal. calcd. for $C_{23}H_{22}N_4O_6$ (450.45) C, 61.33; H, 4.92; N, 12.44; Found,%: C, 67.73; H, 5.42; N, 11.81.
;

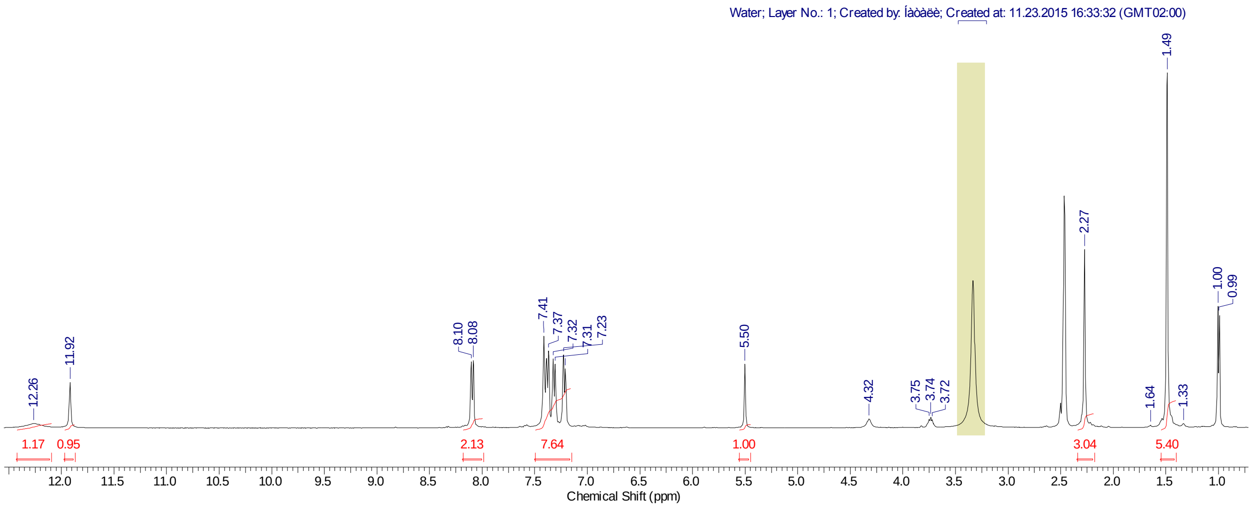

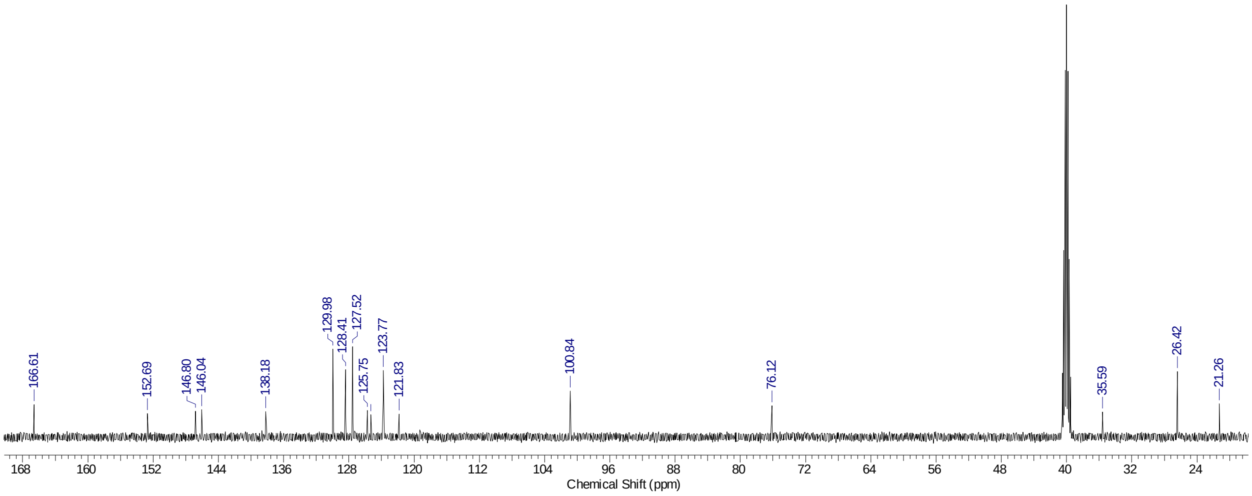

5-((2-Amino-4-(4-fluorophenyl)-1H-imidazol-5-yl)(phenyl)methyl)-6-hydroxy-2,2-dimethyl-4H-1,3-dioxin-4-one (4f)

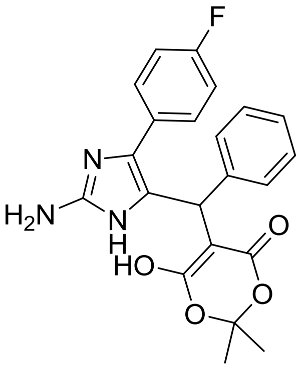

Colorless solid, 54%, mp 238-240 C; IR (KBr, cm^{-1}) ν 3480-2800 (NH_2, NH, OH), 1680 (C=O); ^{1}H NMR (400 MHz, DMSO-d_6) δ=12.19 (br s, 2H, NH, OH), 7.63-7.57 (m, 2H, H_{arom}), 7.41 (br s, 2H, NH_2), 7.32-7.24 (m, 2H, H_{arom}), 7.23-7.17 (m, 2H, H_{arom}), 7.11-7.04 (m, 3H, H_{arom}), 5.41 (s, 1H, CH), 1.51 (s, 6H, CH_3); ^{13}C NMR (125 MHz, DMSO-d_6) δ=166.8 (C=O), 146.6 (C-OH), 143.9, 129.8, 128.4, 127.2, 125.9, 125.5, 120.3, 116.5, 116.3, 100.6, 75.9 (<u>C</u>=COH), 35.0 (CH), 26.4 (CH_3); *m/z* (EI, 70 eV): 307 (100) (409 [$M^{+\bullet}$]-44-58), 265 (17), 223 (16), 122 (26), 43 (33).

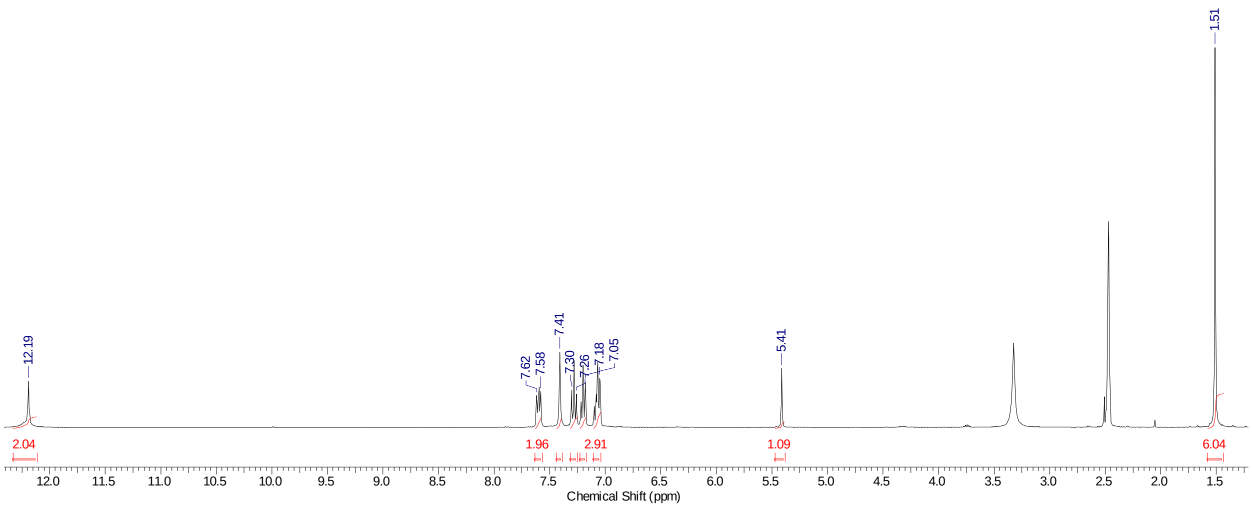

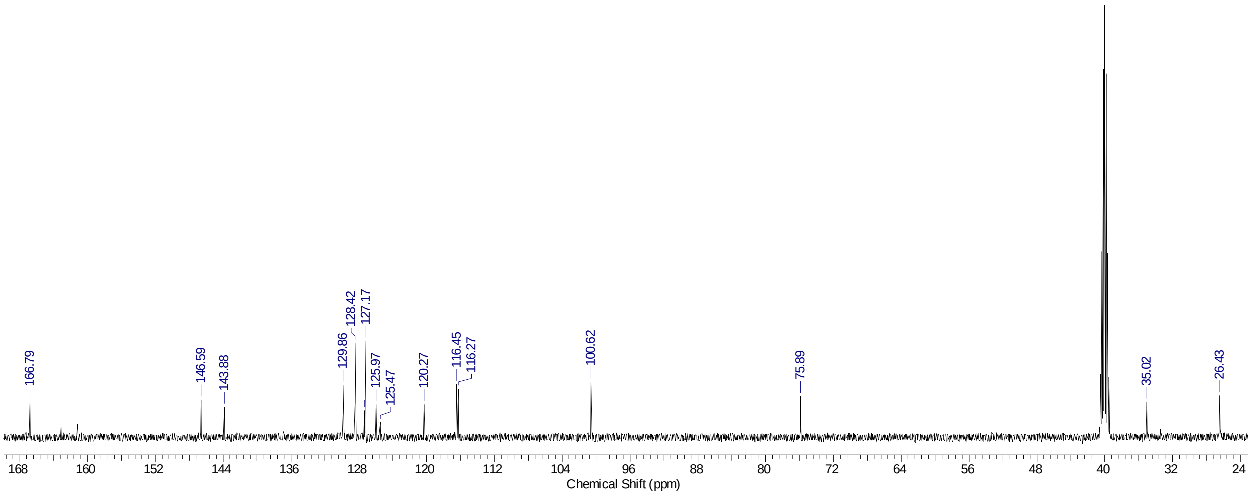

5-((2-Amino-4-(4-fluorophenyl)-1H-imidazol-5-yl)(p-tolyl)methyl)-6-hydroxy-2,2-dimethyl-4H-1,3-dioxin-4-one (4g)

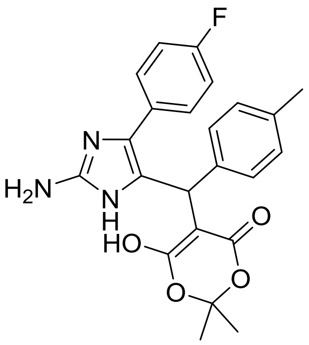

Pale yellow solid, 50%, mp 230-232 C;^{1}H NMR (400 MHz, DMSO-d_6) δ=12.24 (br s, 2H, NH, OH), 7.59-7.53 (m, 2H, H_{arom}), 7.39 (br s, 2H, NH_2), 7.31-7.23 (m, 2H, H_{arom}), 6.99 (d, 2H, *J* 7.8 Hz, H_{arom}), 6.93 (d, 2H, *J* 7.8 Hz, H_{arom}), 5.36 (s, 1H, CH), 2.18 (s, 3H, CH_3) 1.49 (s, 6H, CH_3); ^{13}C NMR (125 MHz, DMSO-d_6) δ 166.8 (C=O), 146.5 (C-OH), 140.9, 134.8, 129.8, 128.99, 127.6, 127.1, 125.6, 120.1, 116.4, 116.3, 100.6, 76.1 (<u>C</u>=COH), 34.7 (CH), 26.4 (CH_3), 20.9 (CH_3); LC-MS: 422 (M-H), 424 (M+H).

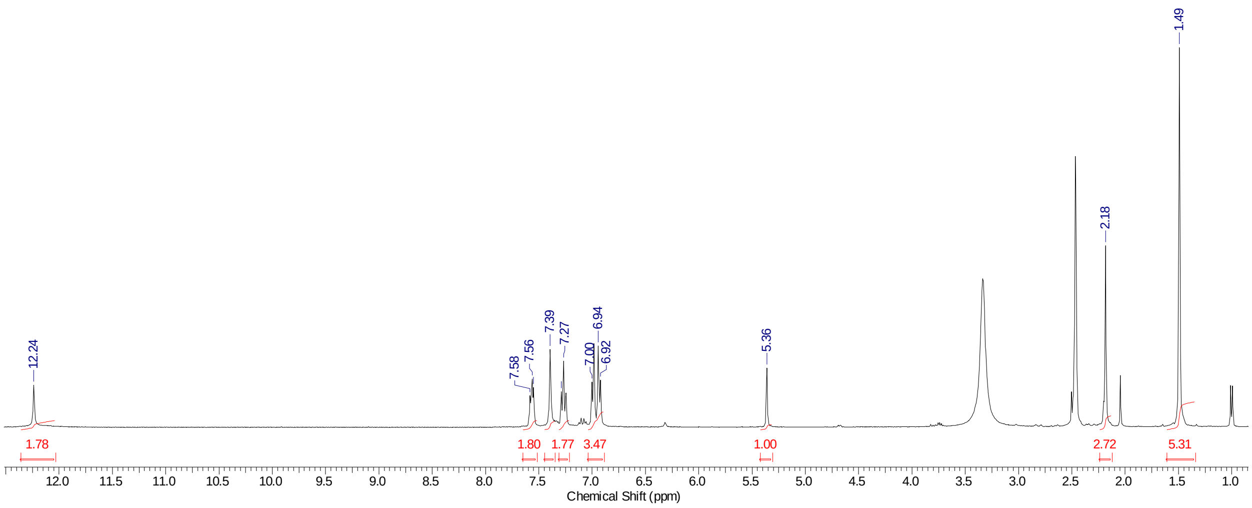

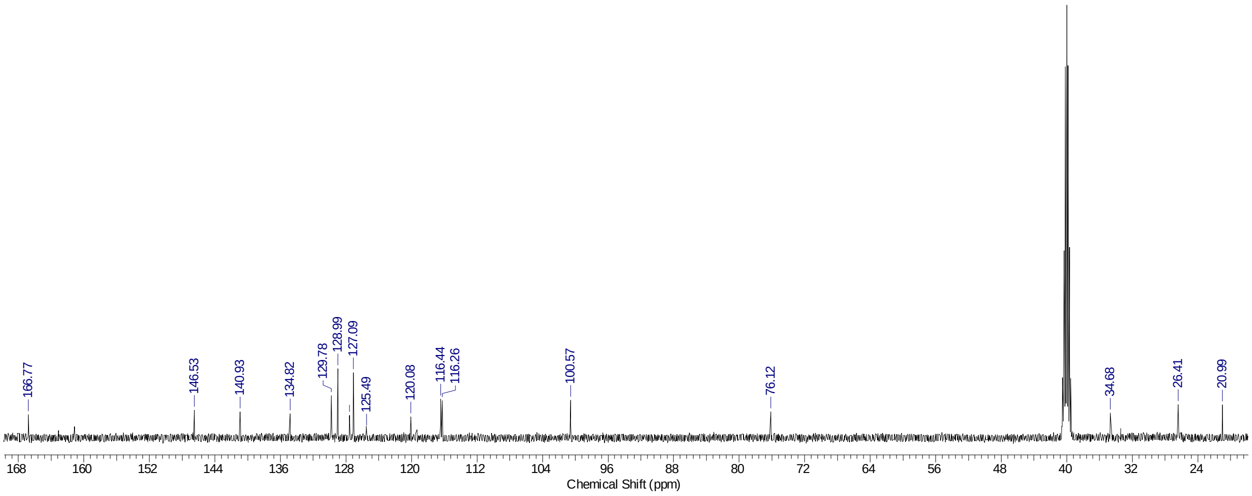

5-((2-Amino-4-(p-tolyl)-1H-imidazol-5-yl)(3-hydroxy-4-methoxyphenyl)methyl)-6-hydroxy-2,2-dimethyl-4H-1,3-dioxin-4-one (4h)

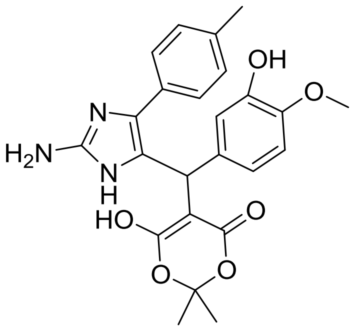

Pale yellow solid, 70%, mp 250-252 C; ^{1}H NMR (200 MHz, DMSO-d_6) δ=12.09 (br s, 2H, NH, OH), 8.68 (br s, 1H, OH_{phenol}), 7.45 (d, 2H, *J* 7.9 Hz, 2,6-H_{arom}), 7.20 (d, 2H, *J* 7.9 Hz, 3,5-H_{arom}), 7.12 (br s, 2H, NH_2), 6.69 (s, 1H, 2-H_{arom}), 6.57 (d, 1H, *J* 7.9 Hz, 5-H_{arom}), 6.36 (d, 1H, *J* 8.2 Hz, 6-H_{arom}), 5.37 (s, 1H, CH), 3.61 (s, 3H, OCH_3), 2.29 (s, 3H, CH_{3arom}) 1.52 (s, 6H, CH_3); ^{13}C NMR (125 MHz, DMSO-d_6) δ 166.8 (C=O), 147.6 (C-OH), 146.6, 144.8, 138.2, 137.2, 135.4, 129.8, 129.1, 127.3, 122.1, 119.6, 115.3, 111.8, 100.4, 76.41 (C=COH), 55.9 (OCH_3), 34. (CH), 26.4 (CH_3), 26.0 (CH_3), 21.3 (CH_3); LC-MS: 450 (M-H), 451 (M), 452 (M+H). Anal. calcd. for $C_{24}H_{25}N_3O_6$ (451.48) C, 63.85; H, 5.58; N, 9.31;; Found,%: C, 63.65; H, 6.42; N, 9.53.

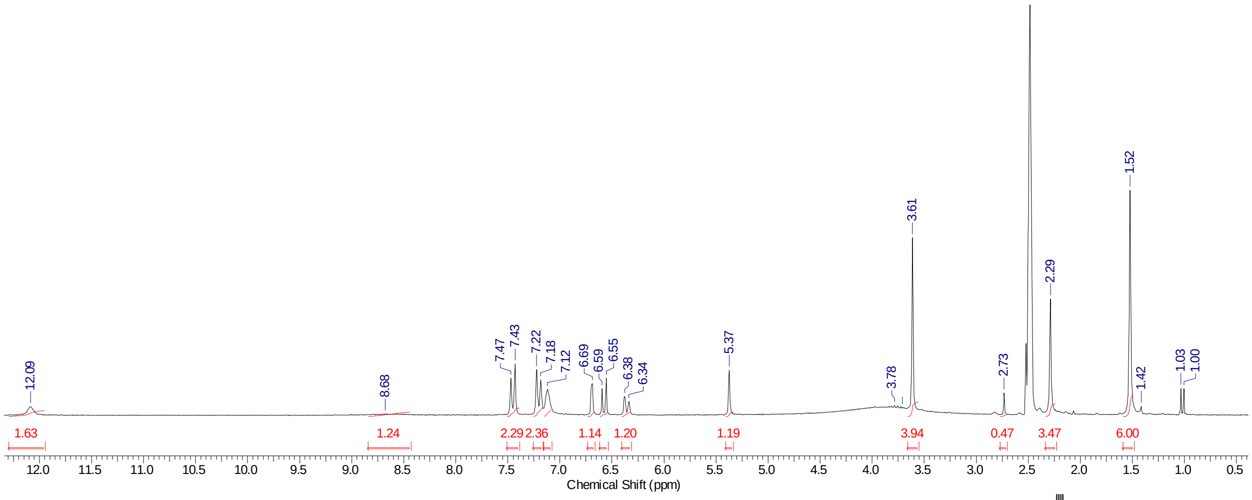

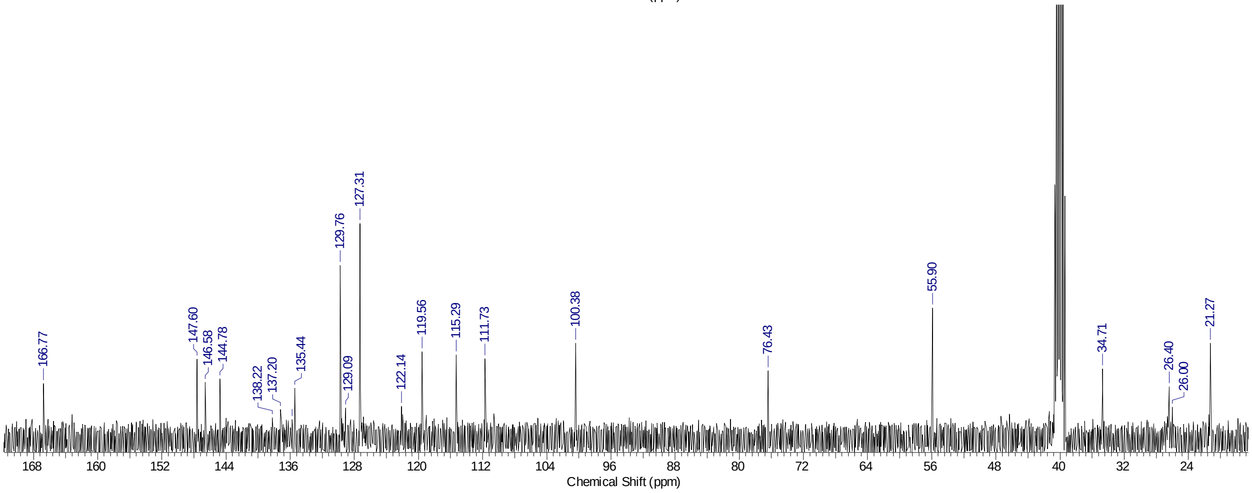

Synthesis of 5-oxo-1,7-diaryl-6,7-dihydro-5*H*-pyrrolo[1,2-*c*]imidazol-3-aminium 2,2,2-trifluoroacetates 9b,c,g

General procedure. A mixture of the corresponding adduct **4** (0.1 mmol) and 0.08 mL (0.11 mmol) TFA was refluxed in 1 mL of toluene for 3 min. After cooling, 3 mL of iPrOH was added to the reaction mixture and the solid product was filtered off, washed with iPrOH and dried on air.

5-Oxo-1-phenyl-7-(p-tolyl)-6,7-dihydro-5H-pyrrolo[1,2-c]imidazol-3-aminium 2,2,2-trifluoroacetate (9b)

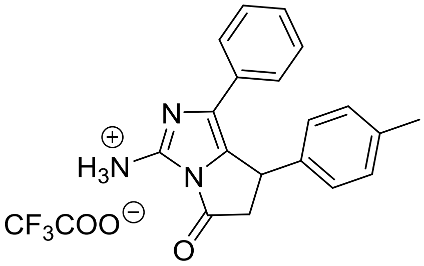

Colorless solid (0.30 g, 52%) of, mp 222-224 C; IR (KBr, cm^{-1}) ν 3432-3160 (NH_3^+, COO^-), 1782 (C=O);^{1}H NMR (400 MHz, DMSO-d_6) δ=8.54 (br s, 2H, NH_3^+), 7.29-7.16 (m, 6H, H_{arom}),

7.15-7.03 (m, 3H, H_{arom}), 4.81 (d, 1H, J 3.6 Hz, CH_X), 3.81 (dd, 1H, J_{BX} 9.3 Hz, J_{AB} 18.6 Hz, CH_B), 2.91 (d, 1H, J_{AX} 3.8 Hz, J_{AB} 18.7 Hz, CH_A), 2.20 (s, 3H, CH_3); ^{13}C NMR (100 MHz, DMSO-d_6): δ=170.2 (C=O), 143.4, 137.7, 137.1, 129.9, 129.4, 129.2, 128.7, 128.4, 127.9, 125.8, 125.5, 46.2, 36.9, 21.1 (CH_3); LC-MS: 304 (M-CF_3COO^-), 305 (M-CF_3COO^-+H).

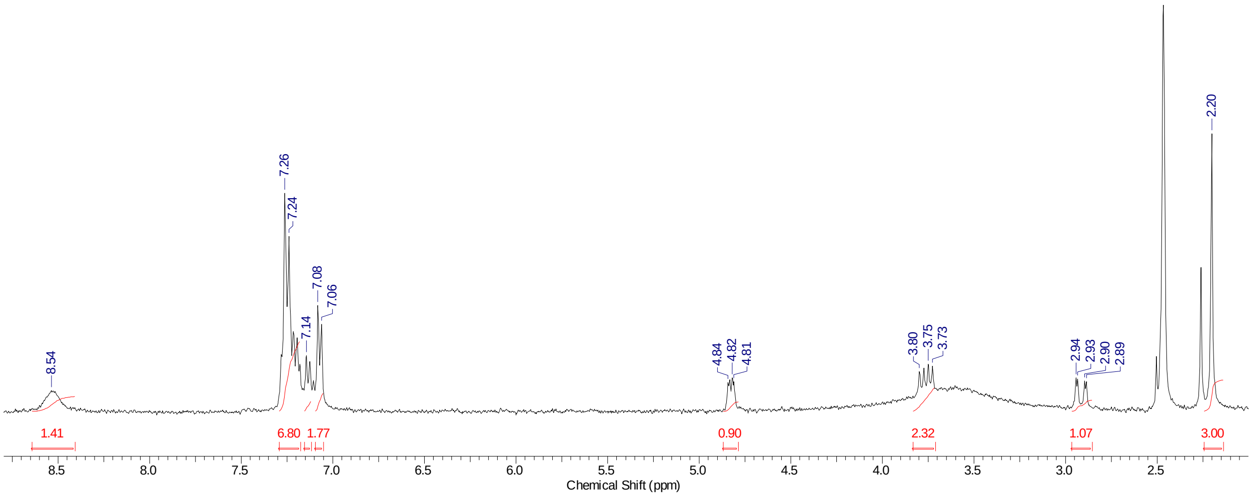

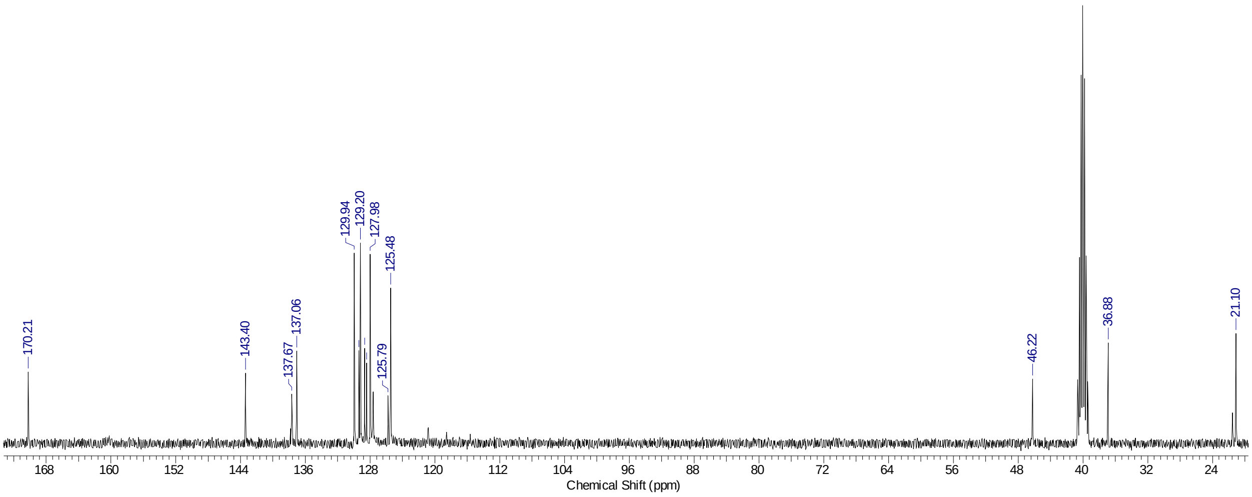

5-Oxo-7-phenyl-1-(p-tolyl)-6,7-dihydro-5H-pyrrolo[1,2-c]imidazol-3-aminium 2,2,2-trifluoroacetate (9c)

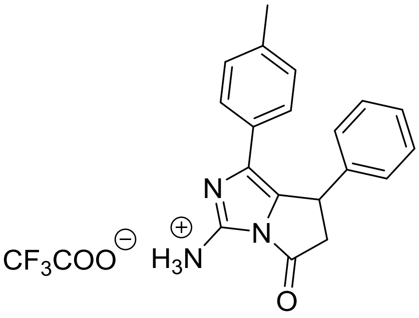

Colorless solid, 60%, mp 202-204 C; ^{1}H NMR (400 MHz, DMSO-d_6) δ=8.59 (br s, 2H, NH_3^+), 7.41-7.34 (m, 2H, H_{arom}), 7.30-7.18 (m, 3H, H_{arom}), 7.14 (d, 2H, J 7.9 Hz, 3,5-H_{arom}), 7.03 (d, 2H, J 7.9 Hz, 2,6-H_{arom}), 4.81 (d, 1H, J 3.4 Hz, CH_X), 3.77 (dd, 1H, J_{BX} 9.7 Hz, J_{AB} 18.9 Hz, CH_B), 2.96 (d, 1H, J_{AX} 3.9 Hz, J_{AB} 18.6 Hz, CH_A), 2.18 (s, 3H, CH_3); ^{13}C NMR (100 MHz, DMSO-d_6): δ=170.1 (C=O), 143.2, 140.7, 138.1, 129.7, 129.4, 128.1, 127.9, 126.7, 125.5, 124.9, 121.1, 46.1, 37.2, 21.1 (CH_3); LC-MS: 304 (M-CF_3COO^-), 305 (M-CF_3COO^-+H).

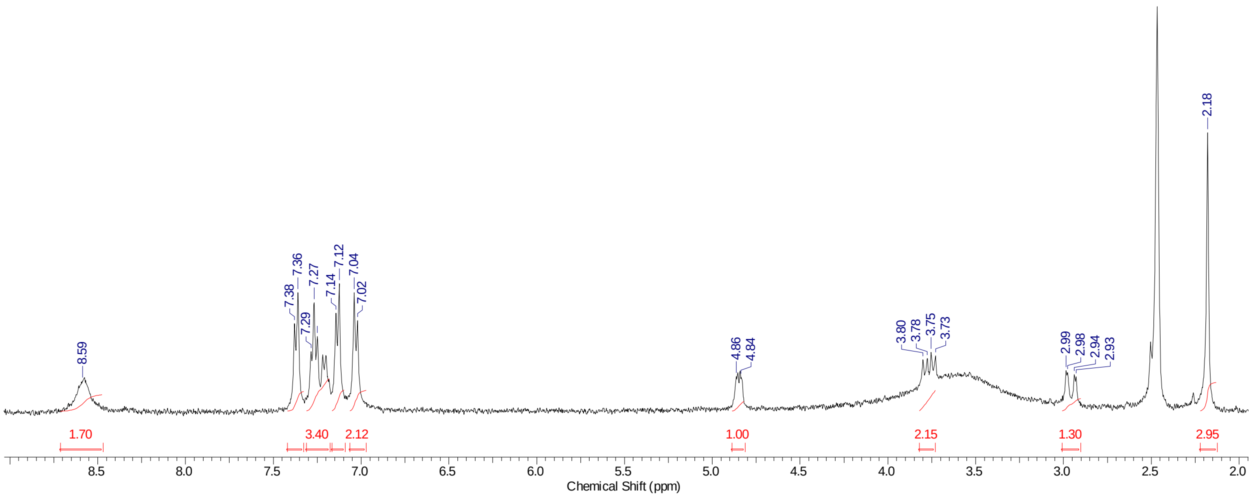

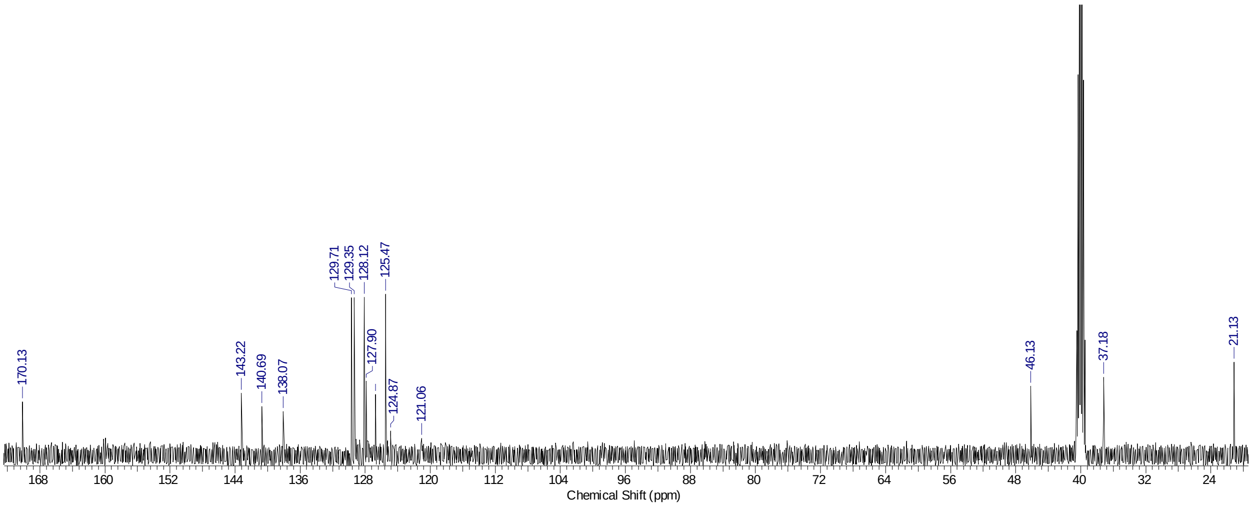

1-([1,1'-Biphenyl]-4-yl)-5-oxo-7-phenyl-6,7-dihydro-5H-pyrrolo[1,2-c]imidazol-3-aminium 2,2,2-trifluoroacetate (9i)

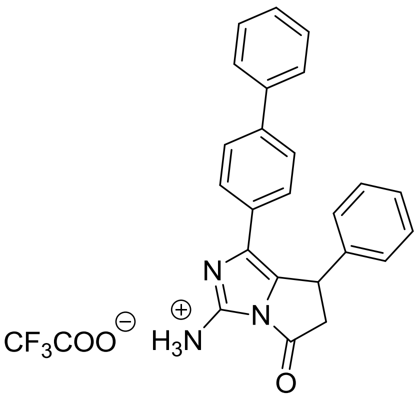

White solid, 71%, mp 228-230 C; IR (KBr, cm^{-1}) ν 3444-3088 (NH_3^+, COO^-), 1780 (C=O); ^{1}H NMR (400 MHz, DMSO-*d*$_6$) δ 8.24 (br s, 2H, NH_3^+), 7.66-7.51 (m, 4H, H_{arom}), 7.48-7.18 (m, 10H, H_{arom}), 4.91 (d, 1H, *J* 3.7 Hz, CH_X), 3.82 (dd, 1H, J_{BX} 9.5 Hz, J_{AB} 18.9 Hz, CH_B), 2.99 (d, 1H, J_{AX} 3.9 Hz, J_{AB} 18.6 Hz, CH_A); ^{13}C NMR (100 MHz, DMSO-*d*$_6$): δ=170.1 (C=O), 153.9, 149.8, 146.0, 143.4, 140.8, 139.7, 139.4, 129.5, 128.2, 128.1, 127.9, 127.7, 127.2, 126.9, 126.1, 46.6, 37.3; *m/z* (EI, 70 eV): 365 (100) [$M^{+\bullet}$], 336 (15), 322 (10), 260 (12), 219 (10), 181 (11), 117 (16), 77 (12).

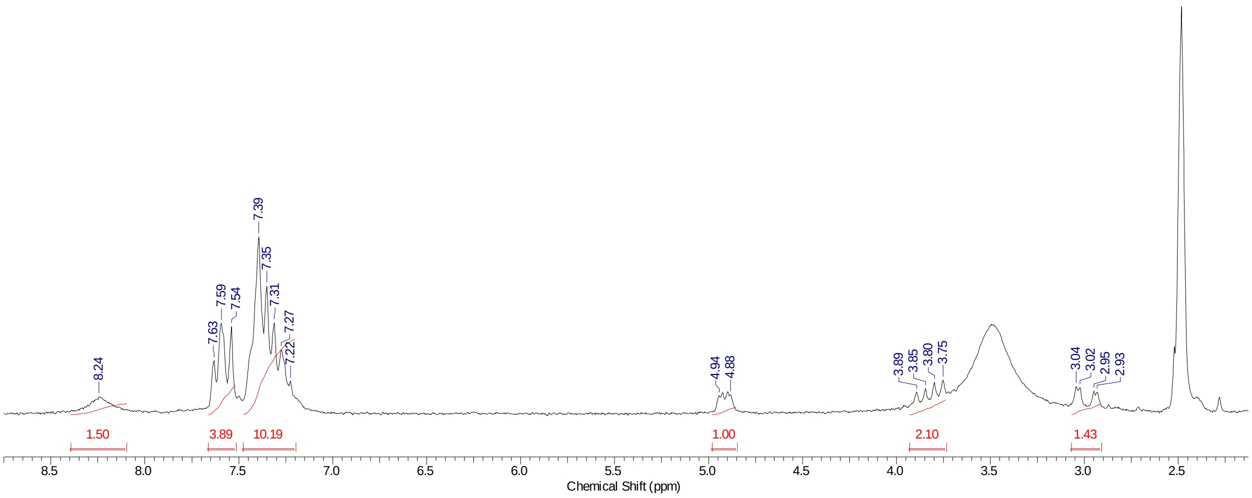

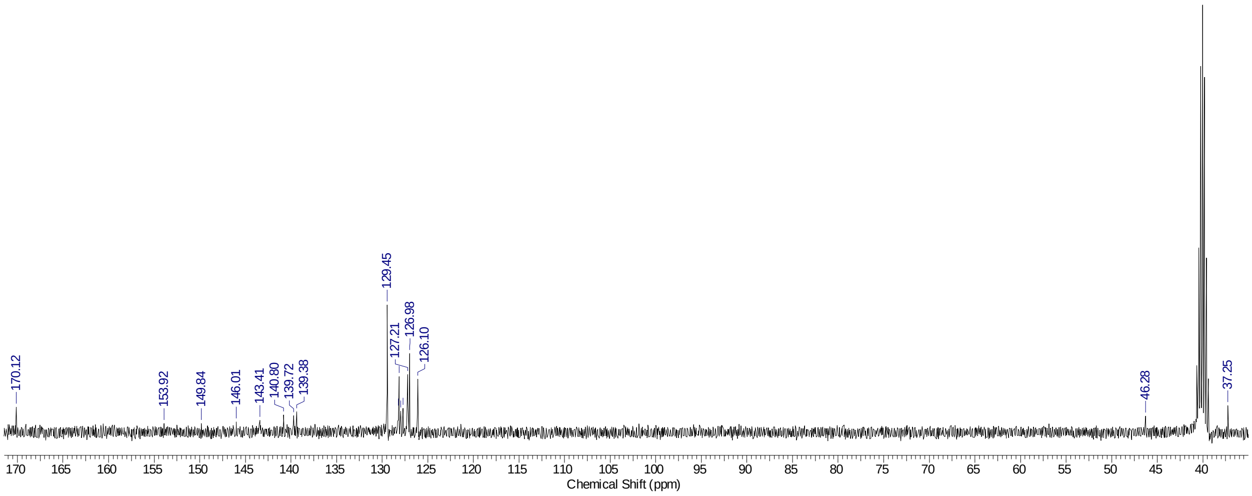

Synthesis of 3-amino-1,7-diphenyl-6,7-dihydro-5*H*-pyrrolo[1,2-*c*]imidazol-5-one (10a)

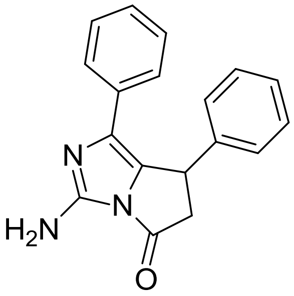

A mixture of the adduct **4a** (0.1 mmol) and 0.08 mL (0.11 mmol) of TFA was stirred in 2 mL of acetonitrile for 6 h, then conc. aqueous solution of NH_3 was added to pH ≈ 8 and the solid product was filtered off, dried on air and crystallized from iPrOH.

Colorless solid, 68%, mp 198-200 C; IR (KBr, cm^{-1}),: 3486-3100 (NH_2 H-связ.), 1784 (C=O); ^{1}H NMR (200 MHz, DMSO-d_6) δ 7.43-6.99 (m, 10H, H_{arom}), 6.36 (br s, 2H, NH_2), 4.75 (d, 1H, J 2.9 Hz, CH_X), 3.83, (dd, 1H, J_{BX} 9.2 Hz, J_{AB} 17.9 Hz, CH_B), 2.87 (d, 1H, J_{AX} 3.3 Hz, J_{AB} 18.6 Hz, CH_A); ^{13}C NMR (125 MHz, DMSO-d_6) δ=): δ=169.6 (C=O), 143.9, 142.5, 133.8, 129.7, 129.4, 128.5, 127.8, 127.6, 127.5, 126.5, 125.4, 47.6, 36.9; *m/z* (EI, 70 eV) 289 (76) [$M^{+\bullet}$], 247 (53), 159 (38), 104 (29), 77 (100), 44 (41), 43 (19). Anal. calcd. for $C_{18}H_{15}N_3O$ (289.12) C, 74.74; H, 5.19; N, 14.53%. Found: C, 72.28; H, 6.79; N, 13.11.

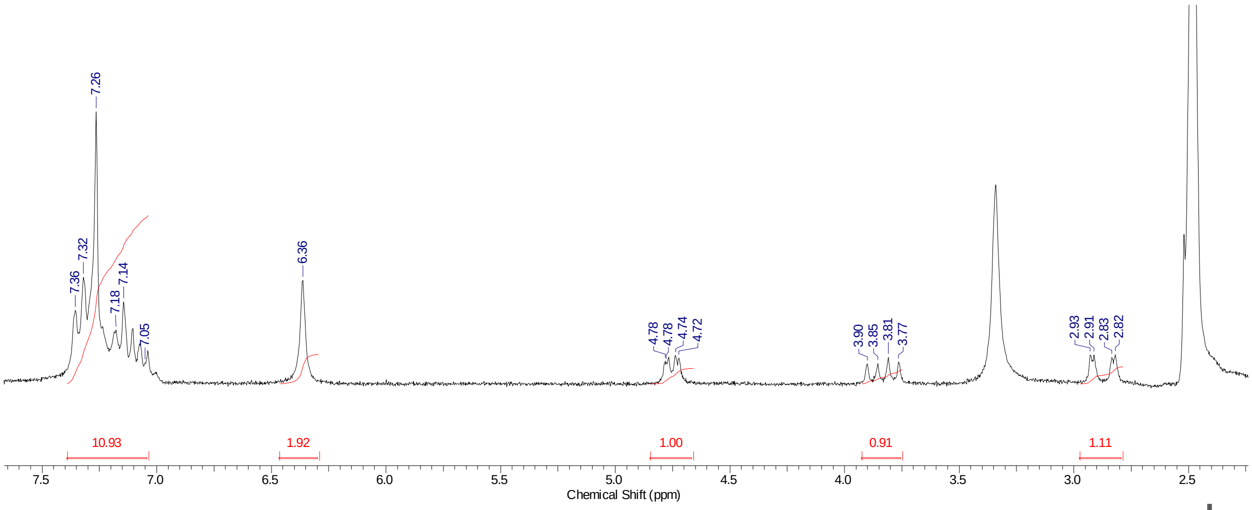

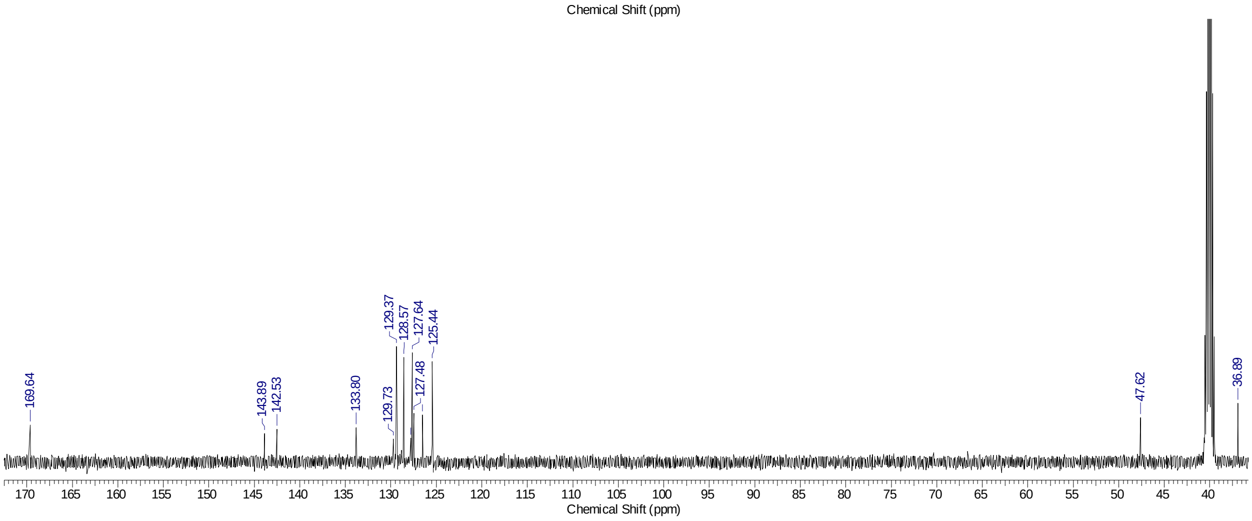

Synthesis of 3-(2-amino-4-aryl-1*H*-imidazol-5-yl)-3-arylpropanoic acids 11b–f,h

General procedure. A mixture of the corresponding adduct **4** (0.1 mmol) and 0.08 mL (0.11 mmol) of TFA was stirred in 2 mL of aqueous acetonitrile for 10–12 h. After cooling, the solid product was filtered off, washed with iPrOH and dried on air.

3-(2-Amino-4-phenyl-1H-imidazol-5-yl)-3-(p-tolyl)propanoic acid (11b)

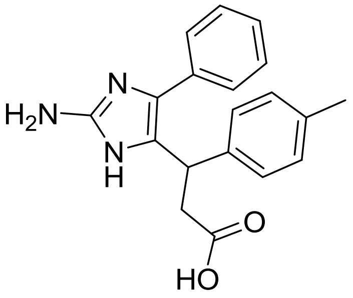

Pale yellow solid solid, 55%, mp 282-285 C; ^{1}H NMR (200 MHz, DMSO-d_6) δ=7.38-7.23 (m, 4H, H_{arom}), 7.22-6.98 (m, 5H, H_{arom}), 5.72 (br s, 2H, NH_2), 4.50-4.37 (m, 1H, CH_X), 3.04-2.71 (m, 2H, H_AH_B), 2.21 (s, 3H, CH_3); ^{13}C NMR (125 MHz, DMSO-d_6): δ=172.8 (COOH), 147.9, 136.6, 135.5, 134.5, 129.8, 129.4, 128.8, 128.1, 127.9, 127.8, 127.4, 36.7, 33.4, 21.0 (CH_3); *m/z*

(EI, 70 eV): 321 (25) [$M^{+\bullet}$], 303 (25), 262 (100), 247 (15), 204 (10), 172 (11), 142 (20), 115 (22), 84 (22). Anal. calcd. for $C_{19}H_{19}N_3O_2$ (321.15) C, 71.01; H, 5.96; N, 13.08%. Found: C, 70.98; H, 6.06; N, 13.85.

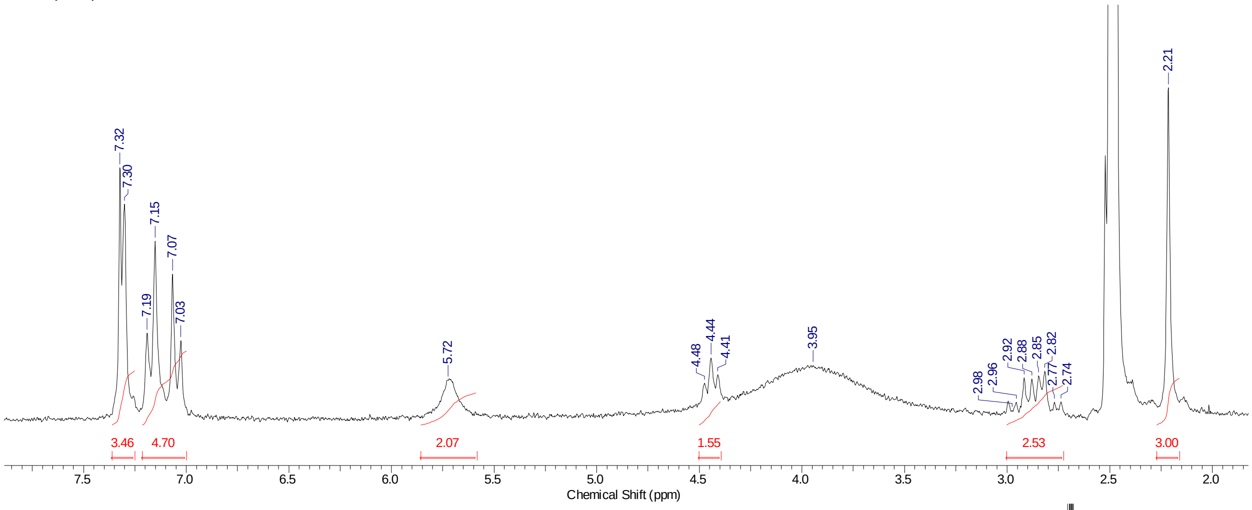

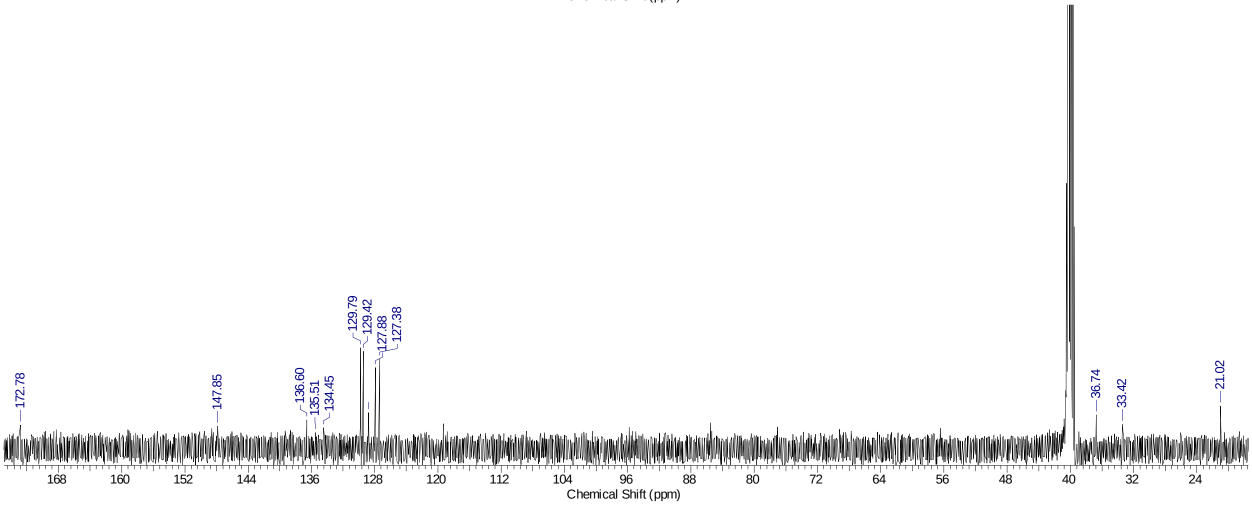

3-(2-Amino-4-(p-tolyl)-1H-imidazol-5-yl)-3-phenylpropanoic acid (11c)

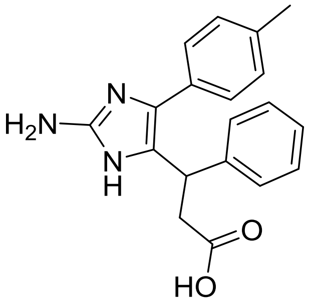

Colorless solid, 76%, mp 265-268 C; ^{1}H NMR (400 MHz, DMSO-d_6) δ=7.31-7.06 (m, 9H, H_{arom}), 5.55 (br s, 2H, NH_2), 4.51-4.37 (m, 1H, CH_X), 3.01-2.76 (m, 2H, H_AH_B), 2.24 (s, 3H, CH_3); ^{13}C NMR (125 MHz, TFA): 160.9 (COOH of 11c and TFA), 145.2, 140.0, 136.6, 128.7, 128.1, 127.0, 126.4, 125.4, 123.5, 121.9, 121.7, 36.6, 35.5, 18.3 (CH_3); LC-MS: 320 (M-H), 321 (M), 322 (M+H). Anal. calcd. for $C_{19}H_{19}N_3O_2$ (321.15) C, 71.01; H, 5.96; N, 13.08%. Found: C, 69.93; H, 5.97; N, 13.63.

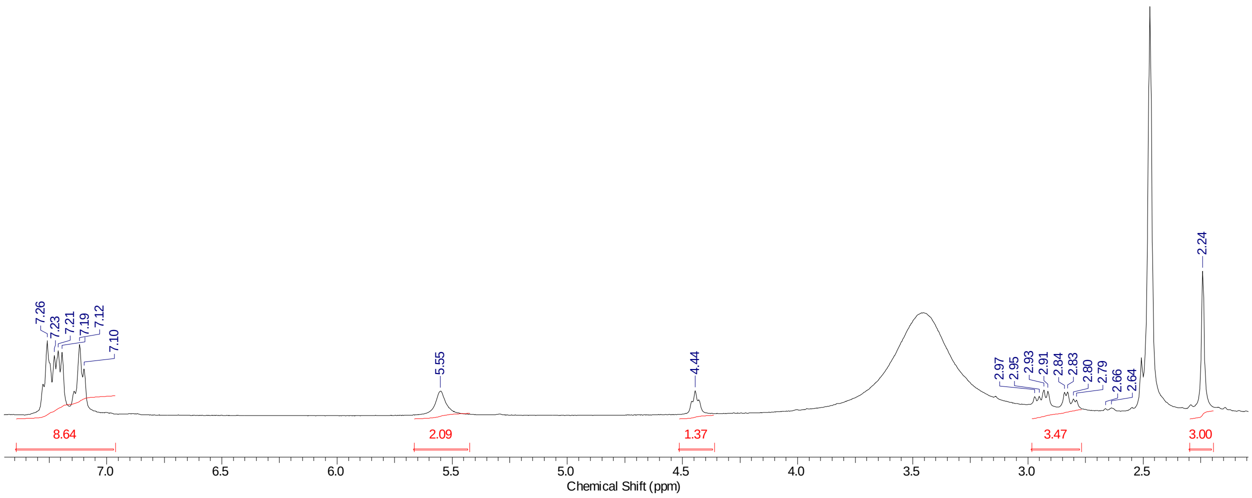

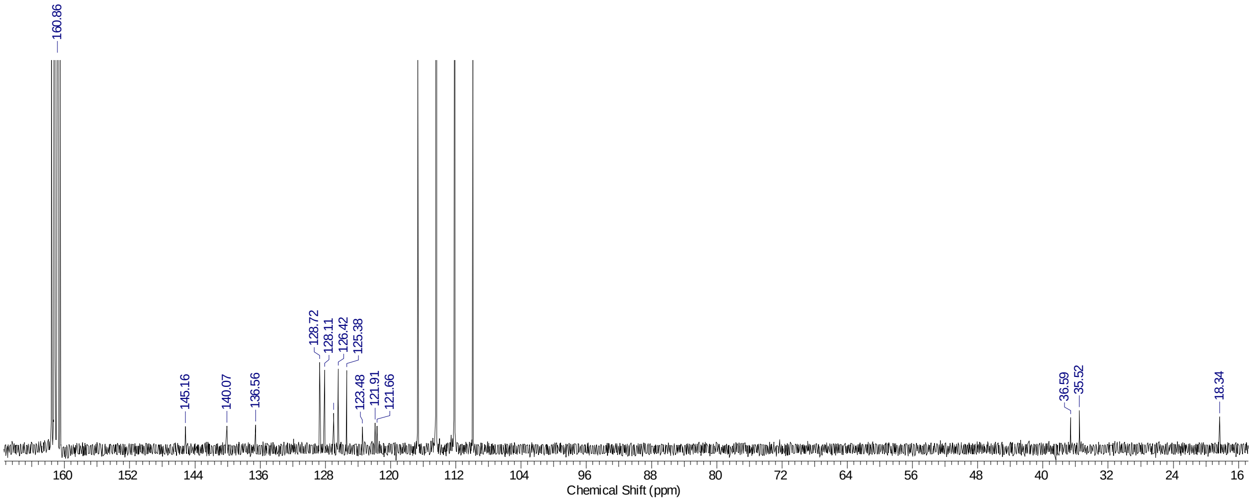

3-(2-Amino-4-(p-tolyl)-1H-imidazol-5-yl)-3-(p-tolyl)propanoic acid (11d)

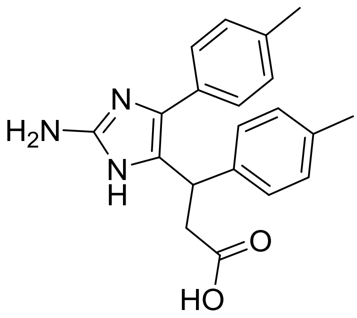

Pale yellow solid, 65%, mp 263-265 C; ^{1}H NMR (400 MHz, DMSO-d_6) δ 7.27-6.96 (m, 8H, H_{arom}), 5.65 (br s, 2H, NH_2) 4.43-4.34 (m, 1H, H_X), 2.98-2.77 (m, 2H, H_AH_B), 2.23 (s, 3H, CH_3), 2.20 (s, 3H, CH_3); ^{13}C NMR (125 MHz, TFA): 177.8 (COOH), 145.1, 140.1, 137.8, 133.3, 128.7, 126.4, 125.3, 123.3, 121.9, 36.6, 35.2, 18.3 (CH_3), 17.9 (CH_3); LC-MS: 334 (M-H), 335 (M), 336 (M+H). Anal. calcd. for $C_{20}H_{21}N_3O_2$ (335.41) C, 71.62; H, 6.31; N, 12.53. Found: C, 72.28; H, 6.79; N, 13.11.

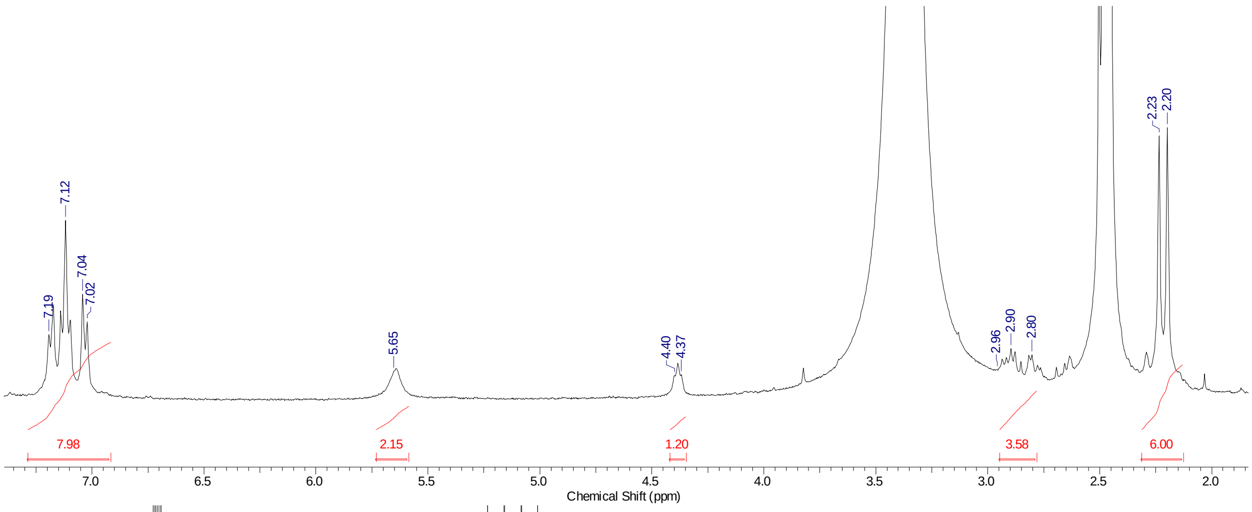

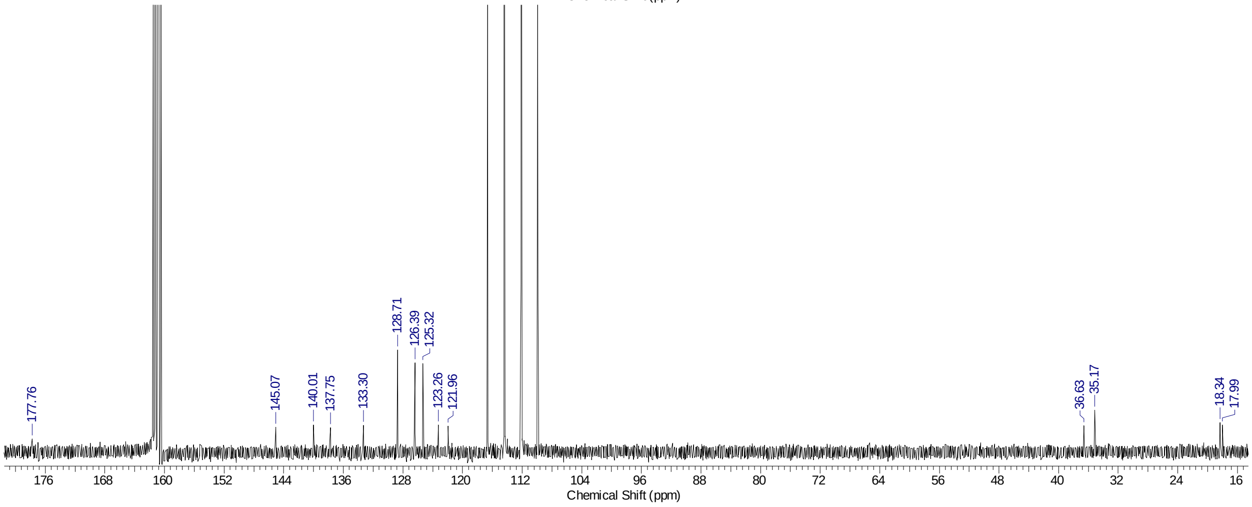

3-(2-Amino-4-(p-tolyl)-1H-imidazol-5-yl)-3-(4-nitrophenyl)propanoic acid (11e).

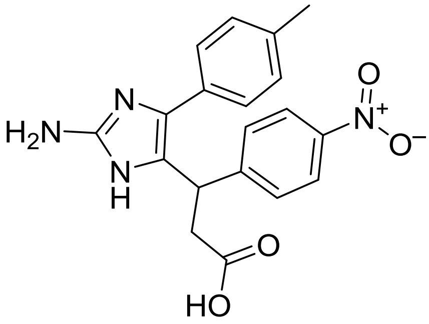

Pale yellow solid, 60%, mp 260-263 C; ^{1}H NMR (400 MHz, DMSO-d_6) δ=8.11 (d, 2H, *J* 8.2 Hz, 2,6-H_{arom} ($C_6H_4NO_2$), 7.53 (d, 2H, *J* 8.6 Hz, 2,6-H_{arom} ($C_6H_4NO_2$), (dd, 4H, *J* = 8 Hz, H_{arom}), 7.22-7.07 (m, 4H, H_{arom}), 5.56 (br s, 2H, NH_2), 4.62-4.57 (m, 1H, H_X), 3.05-2.83 (m, 2H, H_AH_B), 2.24 (s, 3H, CH_3); ^{13}C NMR (125 MHz, TFA): 176.6 (COOH), 145.9, 145.6, 140.5, 128.8, 126.9, 126.5, 123.3, 121.5, 119.9, 36.2, 35.4, 18.3 (CH_3); LC-MS: 365 (M-H), 366 (M), 367 (M+H). Anal. calcd. for $C_{19}H_{18}N_4O_4$ (366.38) C, 62.29; H, 4.95; N, 15.29 Found: C, 62.07; H, 5.66; N, 15.95.

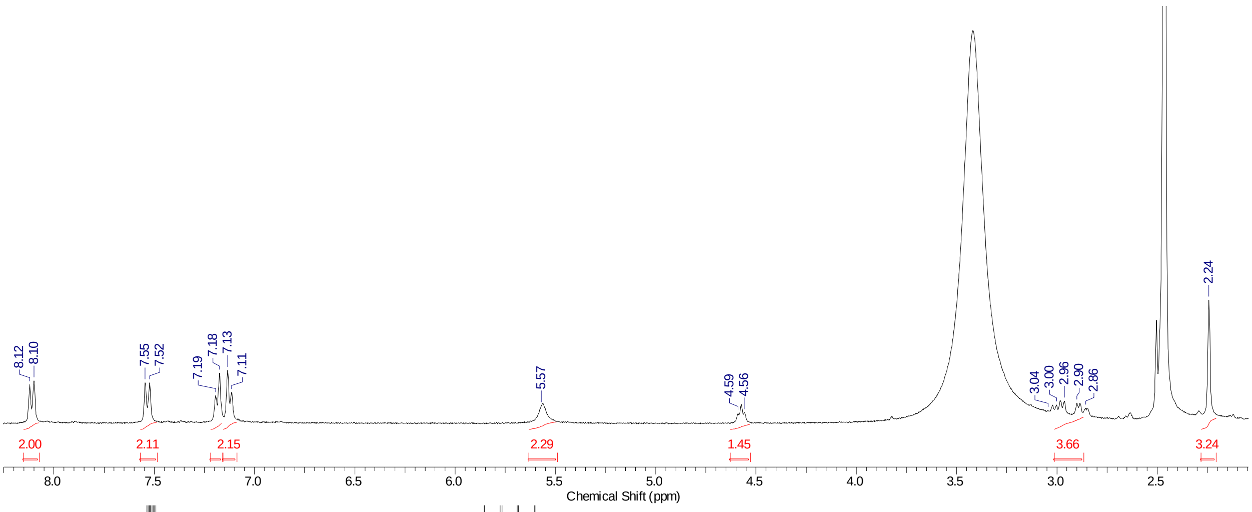

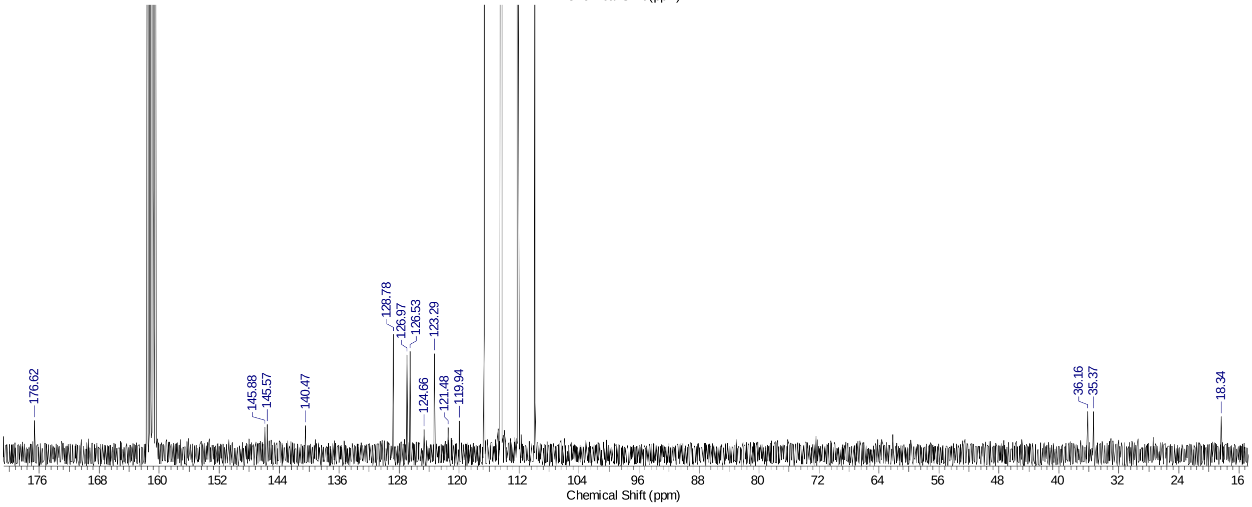

3-(2-Amino-4-(4-fluorophenyl)-1H-imidazol-5-yl)-3-phenylpropanoic acid (11f)

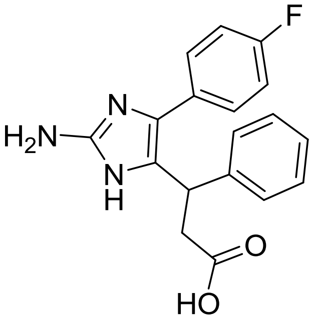

Pale yellow solid, 67%, mp 272-275 C; ^{1}H NMR (400 MHz, DMSO-d_6) δ=7.45-7.08 (m, 9H, H_{arom}), 6.00 (br s, H, NH_2), 4.52-4.41 (m, 1H, H_X), 3.03-2.78 (m, 2H, H_AH_B); ^{13}C NMR (125 MHz, TFA): 178.4 (COOH), 146.2, 137.4, 129.7, 129.0, 127.9, 126.2, 123.3, 122.0, 116.2, 115.9, 37.5, 36.4; *m/z* (EI, 70 eV) 325 (25) [$M^{+\bullet}$], 307 (10), 280 (25), 266 (48), 222 (10), 122 (22), 62 (100), 43 (26).

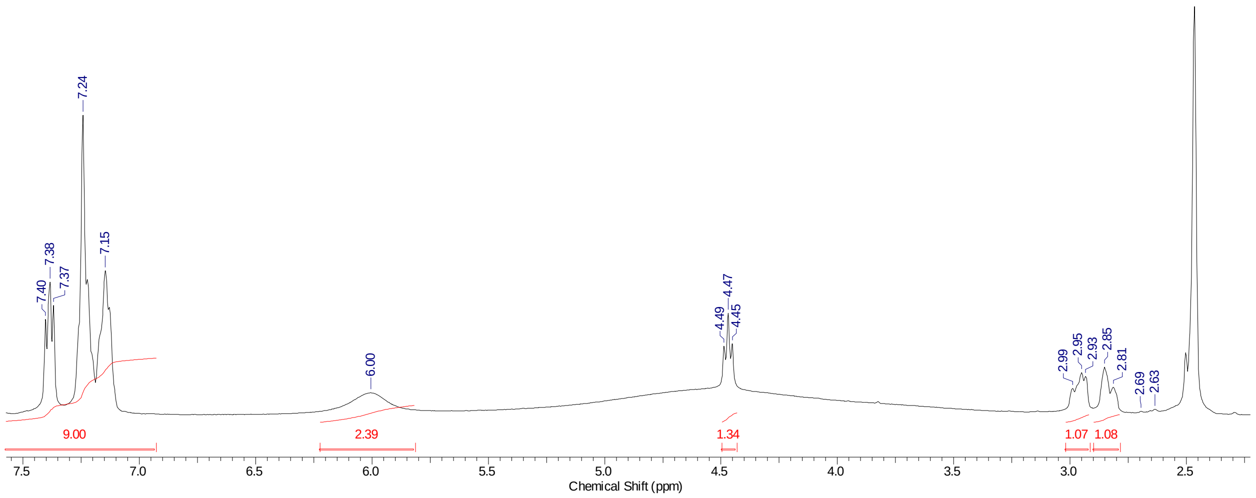

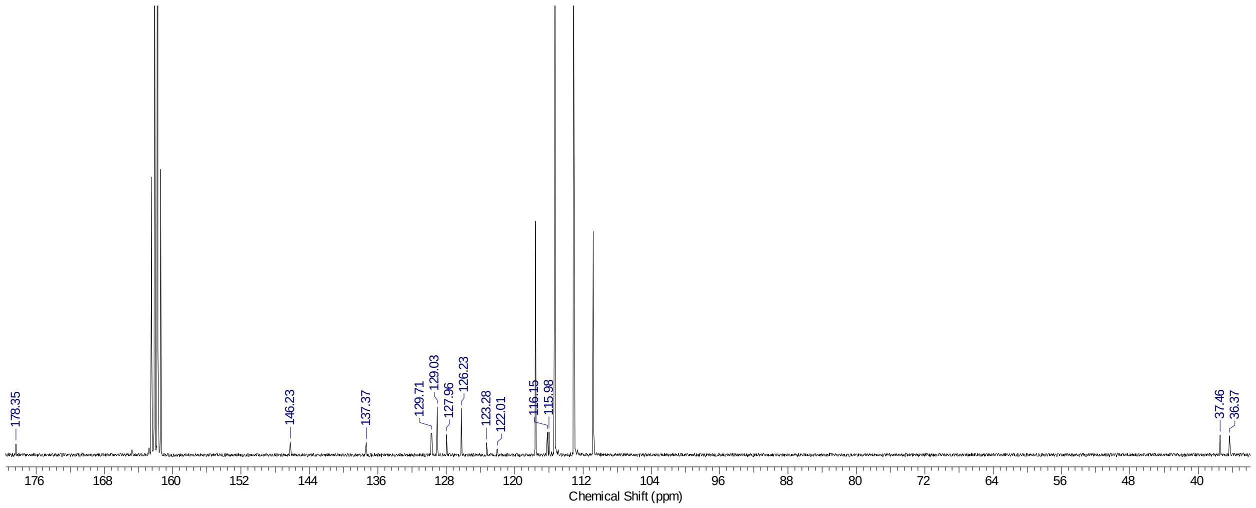

3-(2-Amino-4-(4-fluorophenyl)-1H-imidazol-5-yl)-3-(p-tolyl)propanoic acid (11g)

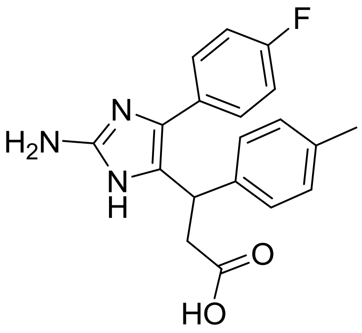

Pale yellow solid, 85%, mp 260-263 C; ^{1}H NMR (400 MHz, DMSO-d_6) δ=7.39-7.22 (m, 2H, H_{arom}), 7.18-6.97 (m, 6H, H_{arom}), 5.46 (br s, H, NH_2), 4.45-4.30 (m, 1H, H_X), 3.01-2.74 (m, 2H, H_AH_B), 2.20 (s, 3H, CH_3); ^{13}C NMR (125 MHz, TFA): 177.5 (COOH), 145.2, 137.8, 133.2, 129.7, 128.8, 125.2, 122.5, 122.2, 121.2, 115.2, 115.0, 36.6, 35.1, 17.9 (CH_3); *m/z* (EI, 70 eV) 339 (30) [$M^{+\bullet}$], 321 (25), 294 (46), 264 (50), 202 (15), 122 (28), 62 (100), 49 (26).

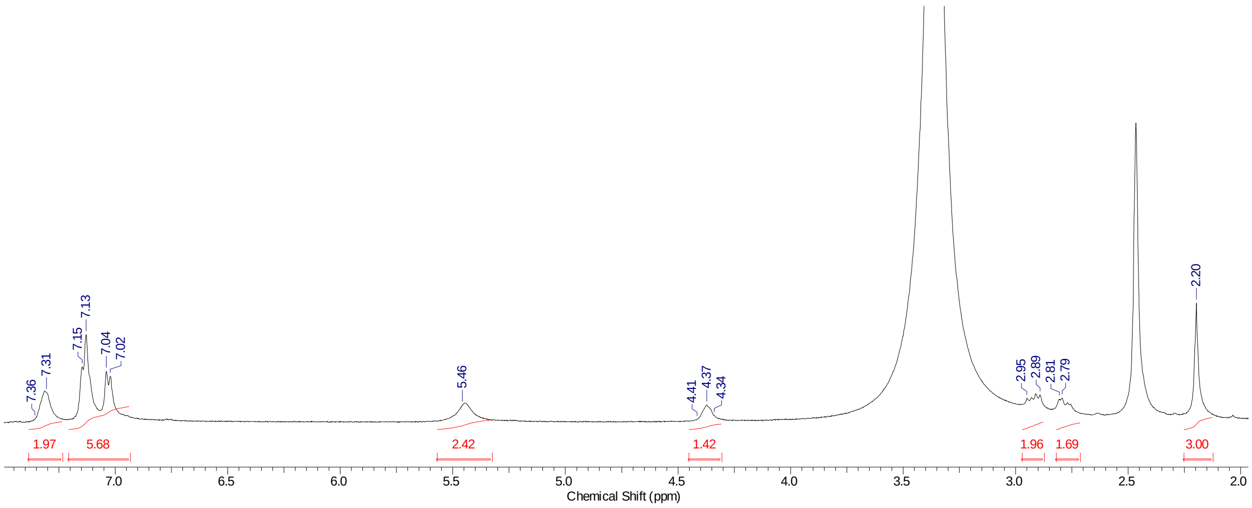

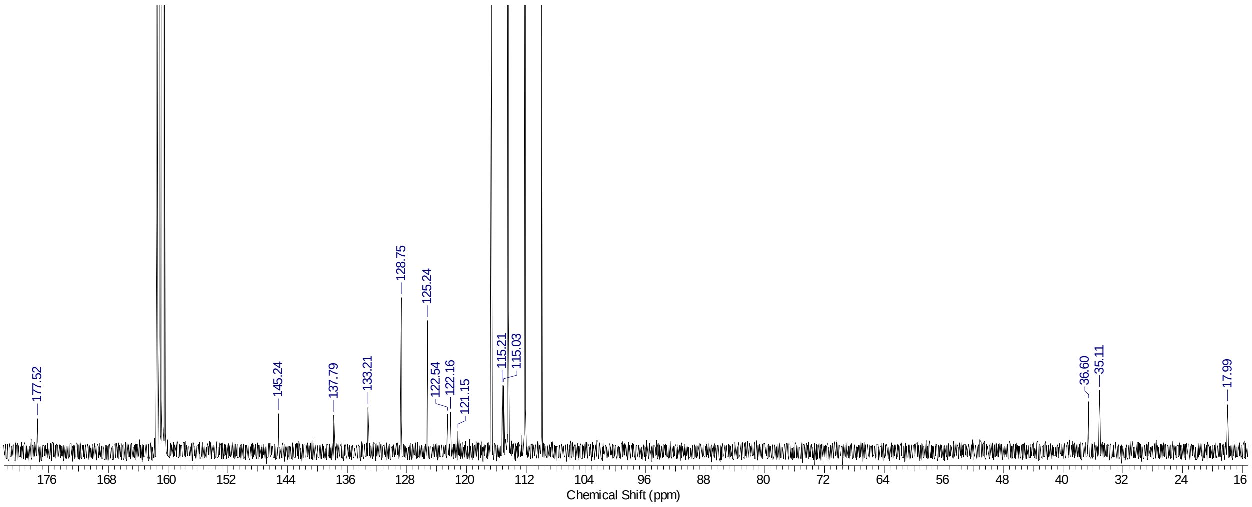

Synthesis of 5-amino-3-(arylideneamino)-1,7-diaryl-7H-pyrrolo[1,2-c]imidazole-6-carbonitriles 14a–f

General procedure. A mixture of the corresponding 2-amino-4-arylimidazole **1** (1.0 mmol), aromatic aldehyde **2** (2.0 mmol) and malononitrile **12** (1.0 mmol) in 2 mL of 2-propanol was refluxed during 20–30 min. After cooling, the yellow solid products **14a–f** were filtered off and crystallized from iPrOH.

5-Amino-3-(4-methylbenzylideneamino)-1-phenyl-7-p-tolyl-7H-pyrrolo[1,2-c]imidazole-6-carbonitrile (14a)

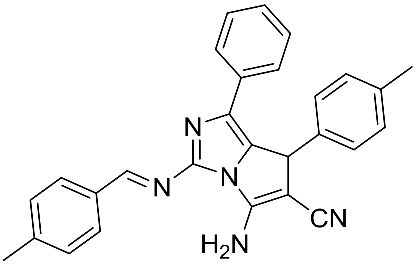

Yellow powder, 65%, mp 221-222 °C; ^{1}H NMR (200 MHz, DMSO-d_6) δ: 9.34 (s, 1H, $CH_{azomethine}$), 8.18 (d, 2H, Ar, J=7.3), 7.68-7.48 (m, 7H, Ar, C^5NH_2), 7.31-7.10 (m, 8H, Ar), 5.34 (s, 1H, C^7H); ^{13}C NMR (125 MHz, DMSO-d_6) δ: 162.4 (C^3), 149.5 ($C_{azomethine}$), 143.8 (C^5), 138.3, 135.3, 133.3, 133.0, 132.5, 132.1, 130.4, 129.5, 129.4, 128.8, 128.2, 128.1, 127.4, 125.8, 117.9 (CN), 71.7 (C^6), 45.01 (C^7); MS (m/z) (%): 429 ([$M^{+\bullet}$],25), 285 (100), 194 (19), 104 (26),

77 (19), 43 (25). Anal. calcd. for $C_{28}H_{23}N_5$ (429.53) C 78.30; H 5.40; N 16.31; Found: C 80.25; H 5.70; N 13.41.

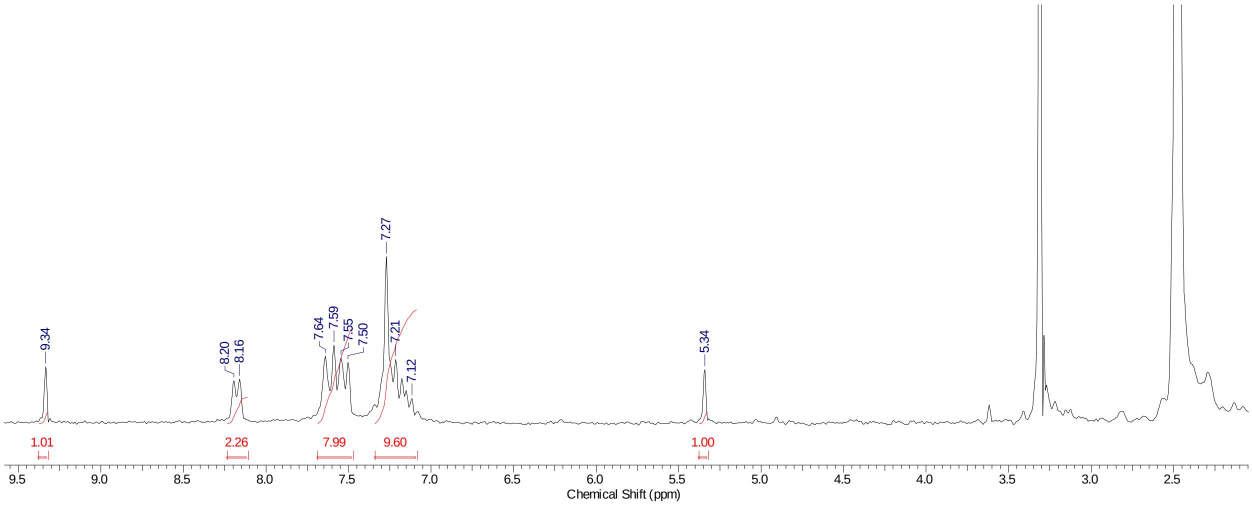

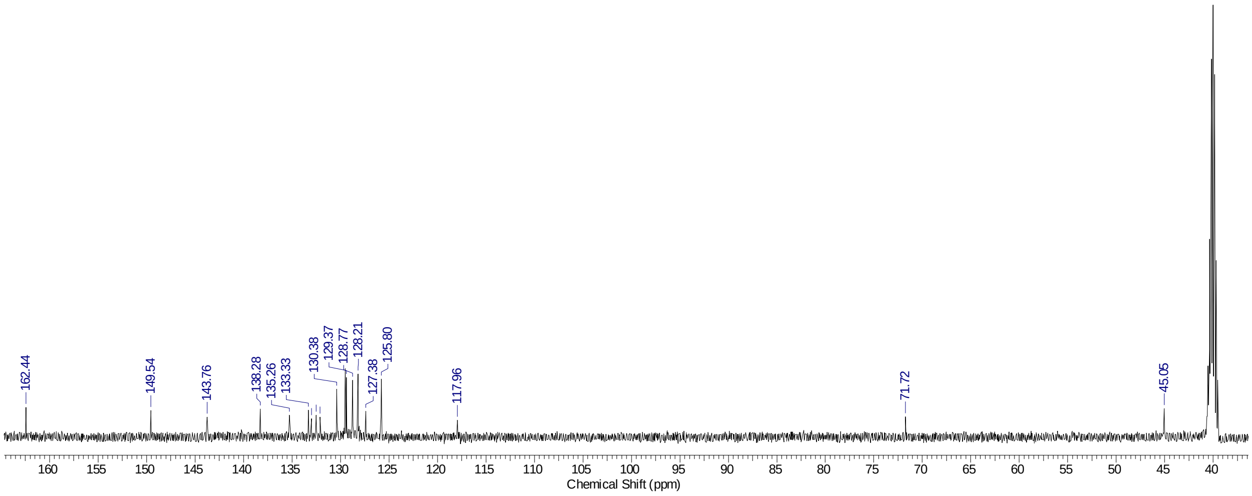

5-Amino-3-(benzylideneamino)-1-(4-chlorophenyl)-7-phenyl-7H-pyrrolo[1,2-c]imidazole-6-carbonitrile (14b)

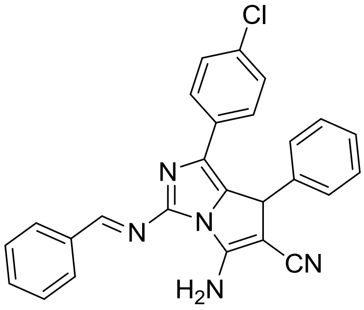

Yellow powder, 83 %, m.p. 244-246 °C. ^{1}H NMR (200 MHz, DMSO-d_6) δ: 9.33 (s, 1H, $CH_{azomethine}$), 8.17 (d, 2H, Ar, J=7.2), 7.68-7.52 (m, 7H, Ar, C^5-NH_2), 7.26-7.37 (m, 7H, Ar), 5.35 (s, 1H, C^7H); ^{13}C NMR (125 MHz, DMSO-d_6) δ: 162.8 (C^3), 149.6 ($C_{azomethine}$), 142.9 (C^5), 137.9, 136.6, 135.2, 135.1, 133.4, 133.0, 131.8, 130.9, 130.5, 129.5, 129.4, 128.8, 128.2, 127.4, 117.9 (CN), 71.7 (C^6), 44.9 (C^7). MS (m/z) (%): *m/z* ($I_{відн}$, %): 435([$M^{+\bullet}$], 100), 306 (51), 270 (55), 228 (10), 155 (16), 105 (33), 91 (13). Anal. calcd. for $C_{26}H_{18}N_5Cl$ (435.91) C 71.64; H 4.16; N 16.07; Found, %: C 73.24; H 4.38; N 15.71.

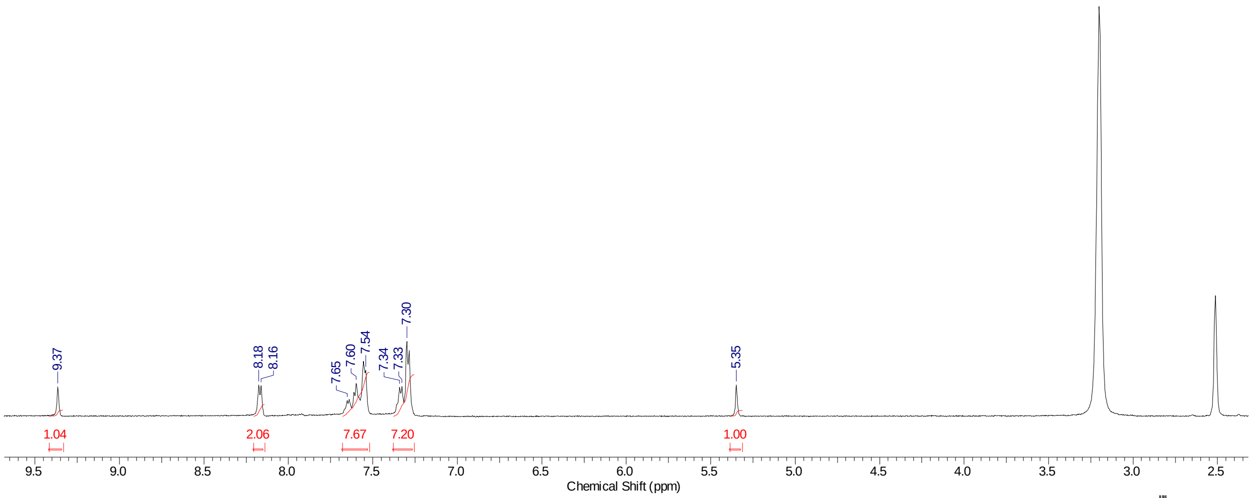

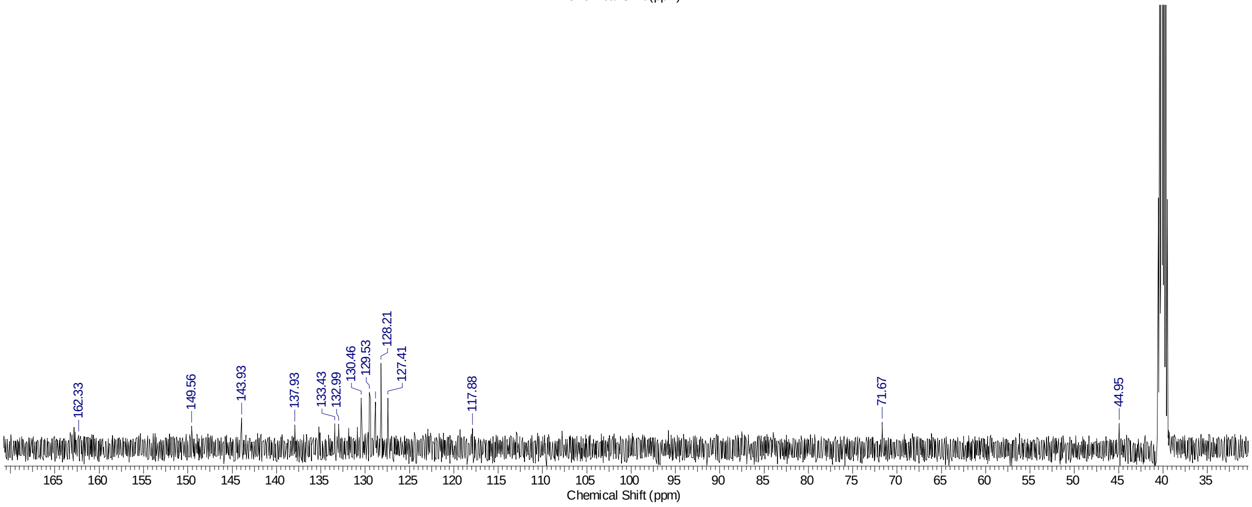

5-Amino-3-(benzylideneamino)-1-(4-methoxyphenyl)-7-phenyl-7H-pyrrolo[1,2-c]imidazole-6-carbonitrile (14c)

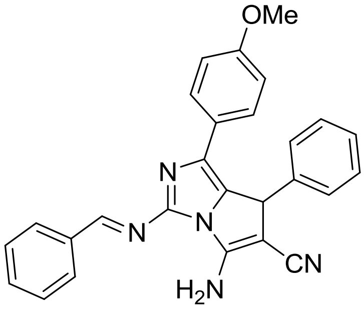

Yellow powder, 45%, m.p. 215-217 °C. ^{1}H NMR (200 MHz, DMSO-d_6) δ: 9.31 (s, 1H, CH$_{azomethine}$), 8.16 (d., 2H, Ar, *J*=7.0), 7.67-7.49 (m, 5H, Ar, C^{5}NH$_2$), 7.43 (d. 2H, Ar, *J*=8.6), 7.34-7.16 (m, 5H, Ar), 6.77 (d. 2H, *J*=8.6, Ar), 5.29 (s, 1H, C^{7}H), 3.67 (s, 3H, Ar OCH$_3$); ^{13}C NMR (125 MHz, DMSO-d_6) δ: 162.0 (C^{3}), 158.8, 149.6 (C$_{azomethine}$), 143.5 (C^{5}), 138.5, 135.3, 133.2, 132.2, 131.0, 130.3, 129.5, 129.4, 128.2, 127.2, 126.5, 125.7, 118.0 (CN), 114.2, 71.7 (C^{6}), 55.6 (OCH$_3$), 44.9 (C^{7}). MS (m/z) (%): 431 ([M$^{+\bullet}$], 69), 301 (100), 270 (54), 178 (40), 116 (22), 91 (24), 83 (56), 57 (63). Anal. calcd. for $C_{27}H_{21}N_5O$ (431.49) C 75.16; H 4.91; N 16.23; Found, %: C 73.05; H 4.12; N 15.73.

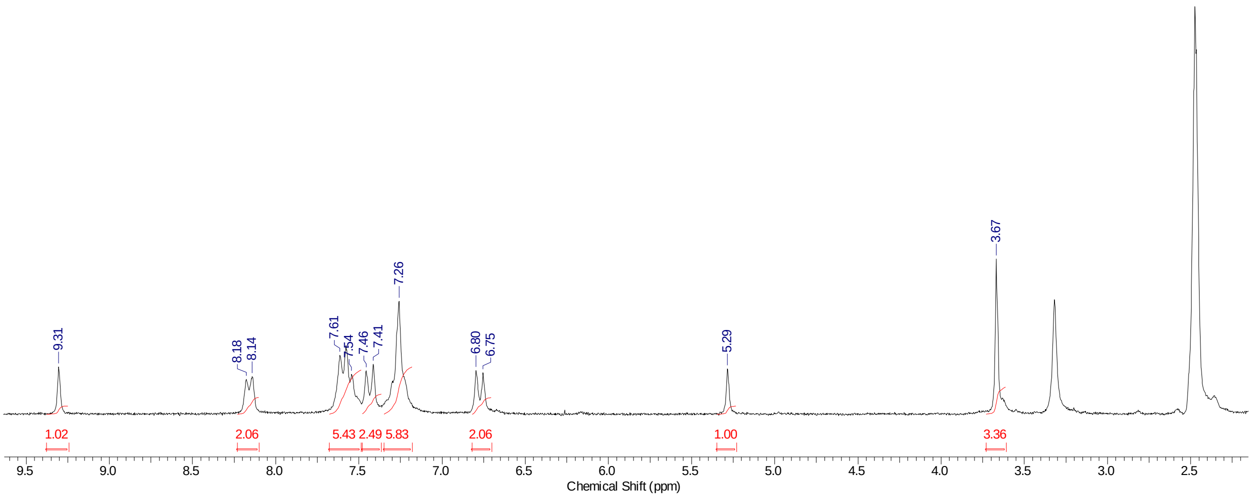

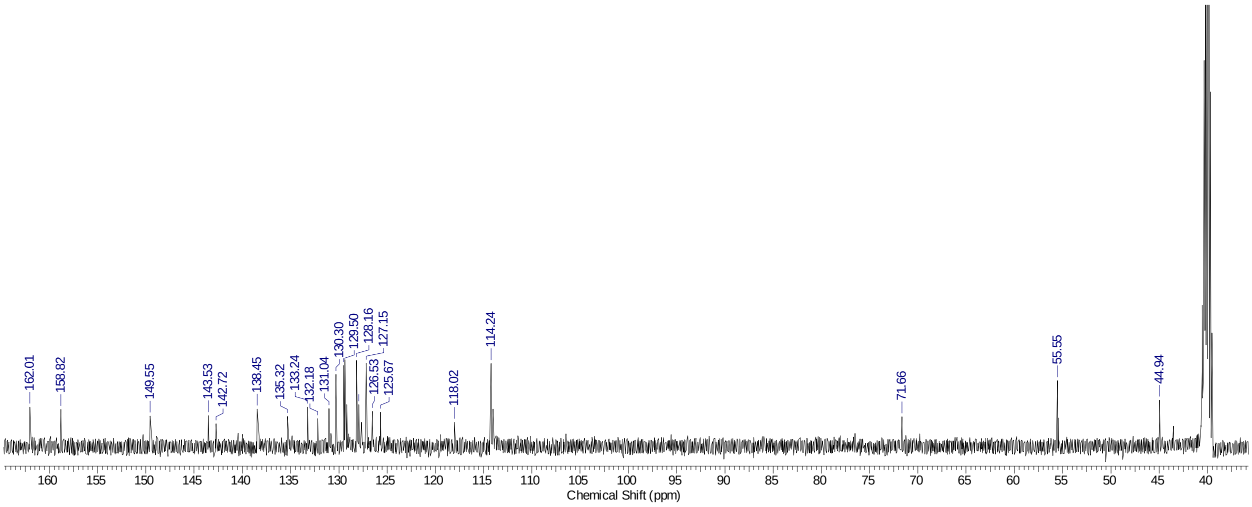

5-Amino-3-((4-bromobenzylidene)amino)-7-(4-bromophenyl)-1-phenyl-7H-pyrrolo[1,2-c]imidazole-6-carbonitrile (14d)

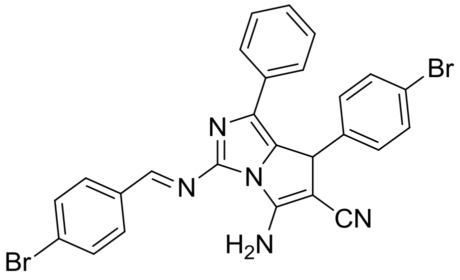

Yellow powder, 40%, m.p. 259-260 °C. ^{1}H NMR (200 MHz, DMSO-d_6) δ: 9.30 (s, 1H, $CH_{azomethine}$), 8.12 (d, 2H, Ar, J=8.2), 7.74 (d, 2H, Ar, J=8.2), 7.64 (br.s, 2H, C^5-NH_2), 7.55-7.44 (m, 5H, Ar), 7.28-7.19 (m, 4H, Ar), 5.37 (s, 1H, C^7H); ^{13}C NMR (125 MHz, DMSO-d_6) δ: 161.5 (C^3), 149.6 ($C_{azomethine}$), 143.7 (C^5), 137.8, 134.5, 133.2, 132.6, 132.3, 130.5, 128.9, 127.6, 127.1, 125.8, 121.2, 117.9 (CN), 71.2 (C^6), 44.2 (C^7); MS (m/z) (%): 559 [M(^{81}Br)+H]$^+$ (5), 560 [M(^{79}Br)+H]$^+$ (10), 137 (100). Anal. calcd. for $C_{26}H_{17}Br_2N_5$ (559.25) C 55.84; H 3.06; N 12.52; Found, %: C 56.58; H 3.61; N 12.95.

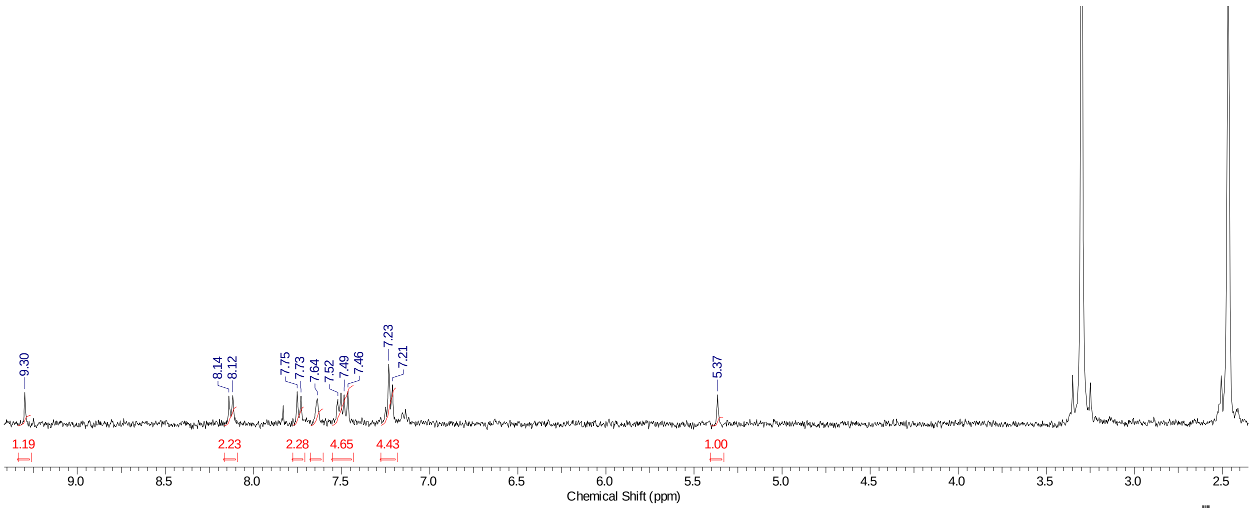

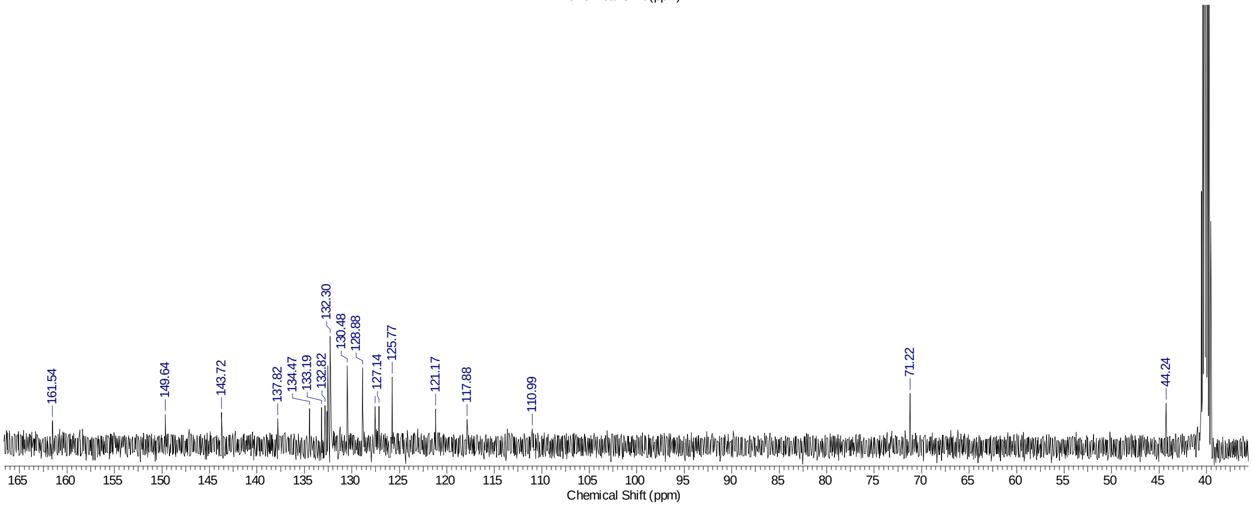

5-Amino-3-((4-fluorobenzylidene)amino)-7-(4-fluorophenyl)-1-phenyl-7H-pyrrolo[1,2-c]imidazole-6-carbonitrile (14e)

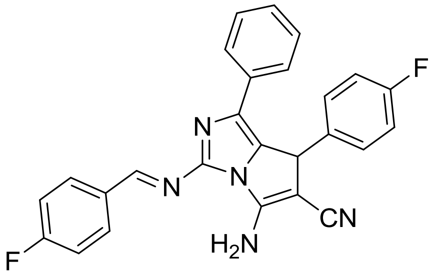

Yellow powder, 58%, m.p 240-242 °C. ^{1}H NMR (200 MHz, DMSO-d_6) δ: 9.31 (s, 1H, $CH_{azomethine}$), 8.32-8.23 (m, 2H, Ar), 7.63 (br.s, 2H, C^5NH_2), 7.50 (d, 2H, Ar, *J*=7.3), 7.41-7.34 (m, 2H, Ar), 7.33-7.27 (m, 2H, Ar), 7.25-7.19 (m, 2H, Ar), 7.17-7.07 (m, 3H, Ar), 5.36 (s, 1H, C^7H); ^{13}C NMR (125 MHz, DMSO-d_6) δ: 161.4 (C^3), 149.6 ($C_{azomethine}$), 149.6 (C^5), 143.8, 133.2, 133.1, 132.9, 132.4, 132.0, 130.3, 130.2, 128.8, 127.4, 125.8, 117.9 (CN), 116.7 (d, J_{CF}=22.4), 116.2 (d, J_{CF}=21.4), 71.4 (C^6), 44.1 (C^7); MS (m/z) (%): 438 $[M+H]^+$ (35), 91 (100).

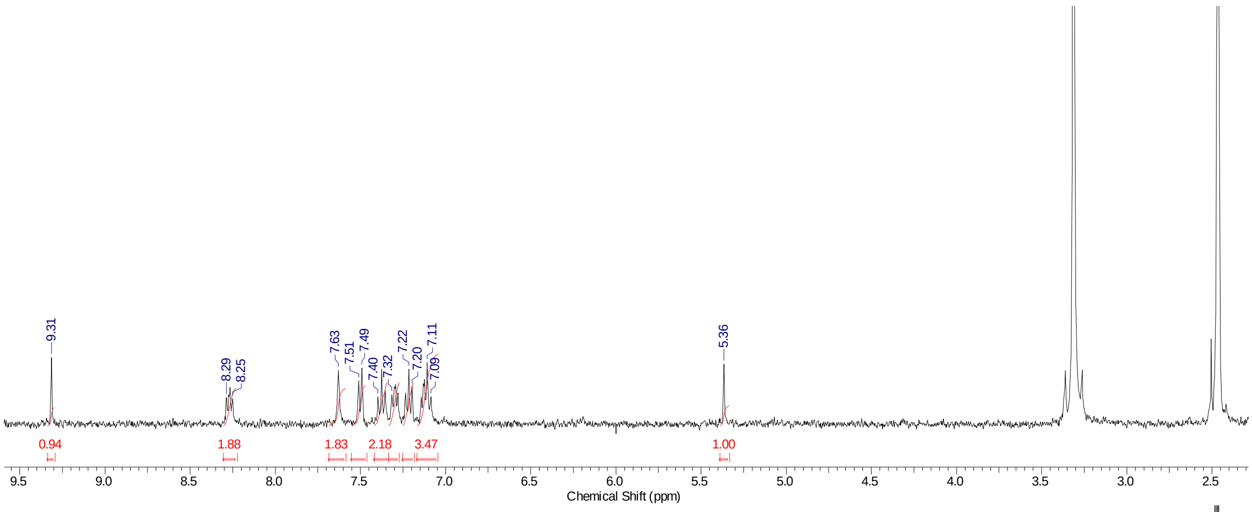

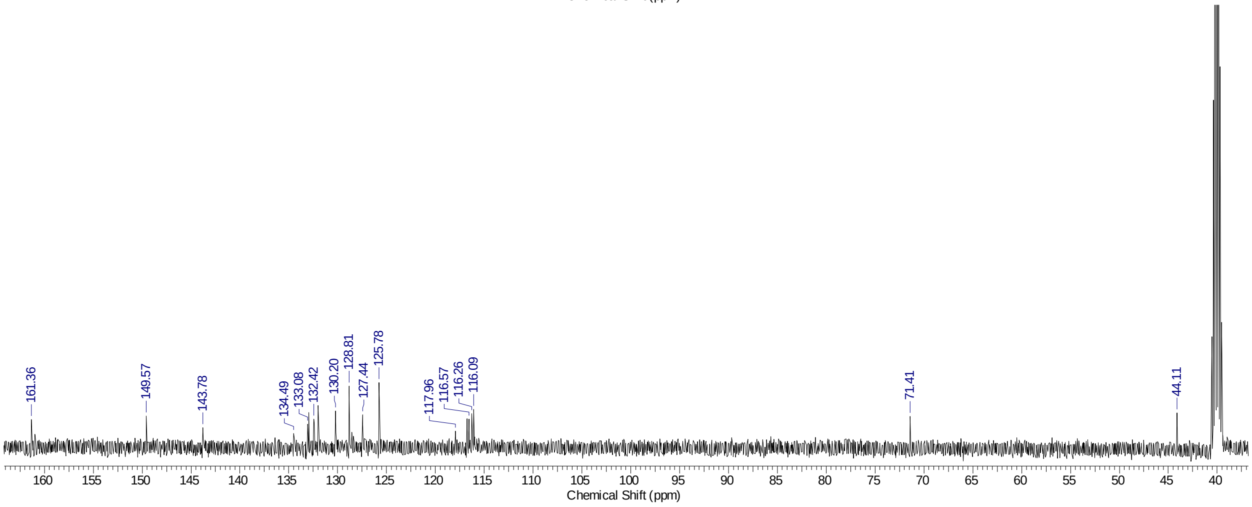

5-Amino-3-((4-bromobenzylidene)amino)-7-(4-bromophenyl)-1-(p-tolyl)-7H-pyrrolo[1,2-c]imidazole-6-carbonitrile (14f)

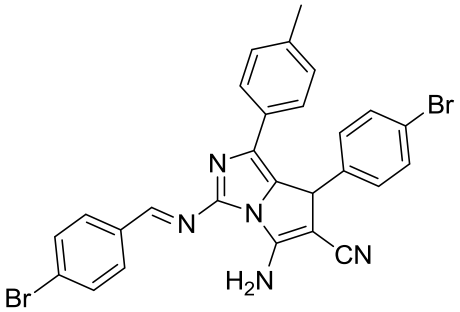

Yellow powder, 59%, m.p. 279-280 °C. ^{1}H NMR (200 MHz, DMSO-d_6) δ: 9.27 (s, 1H, $CH_{azomethine}$), 8.12 (d, 2H, Ar, *J*=8.2), 7.73 (d, 2H, Ar, *J*=7.8), 7.62 (br.s, 2H, $C^{5}NH_2$), 7.47 (d, 2H, Ar, *J*=8.2), 7.40 (d, 2H, Ar, *J*=7.8), 7.20 (d, 2H, Ar, *J*=7.8), 7.03 (d, 2H, Ar, *J*=7.4), 5.33 (s, 1H, $C^{7}H$), 2.20 (s, 3H, Ar CH_3); ^{13}C NMR (125 MHz, DMSO-d_6) δ: 161.3 (C^{3}), 149.6 ($C_{azomethine}$), 143.6 (C^{5}), 137.9, 136.9, 134.5, 132.5, 132.4, 132.3, 132.2, 131.7, 130.5, 129.4, 127.1, 125.7, 121.1, 117.9 (CN), 71.2 (C^{6}), 44.2 (C^{7}), 21.2. MS (m/z) (%): 574 $[M(^{81}Br)+H]^+$ (5), 572 $[M(^{79}Br)+H]^+$ (4), 137 (100). Anal. calcd. for $C_{27}H_{19}Br_2N_5$ (573.28) C 56.57; H 3.34; N 12.22; Found, %: C 57.31; H 4.76; N 13.27.

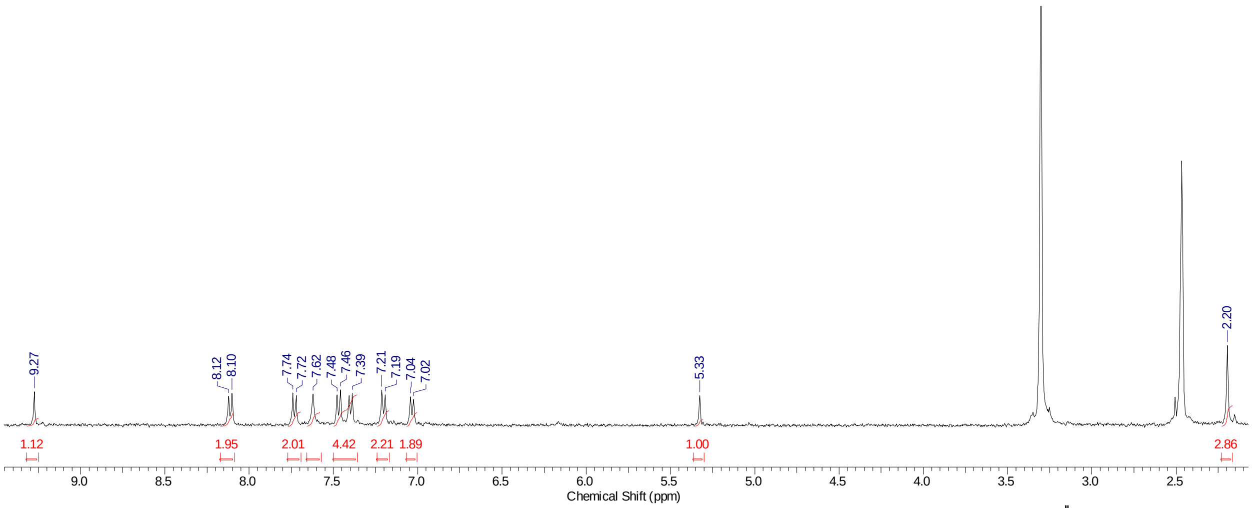

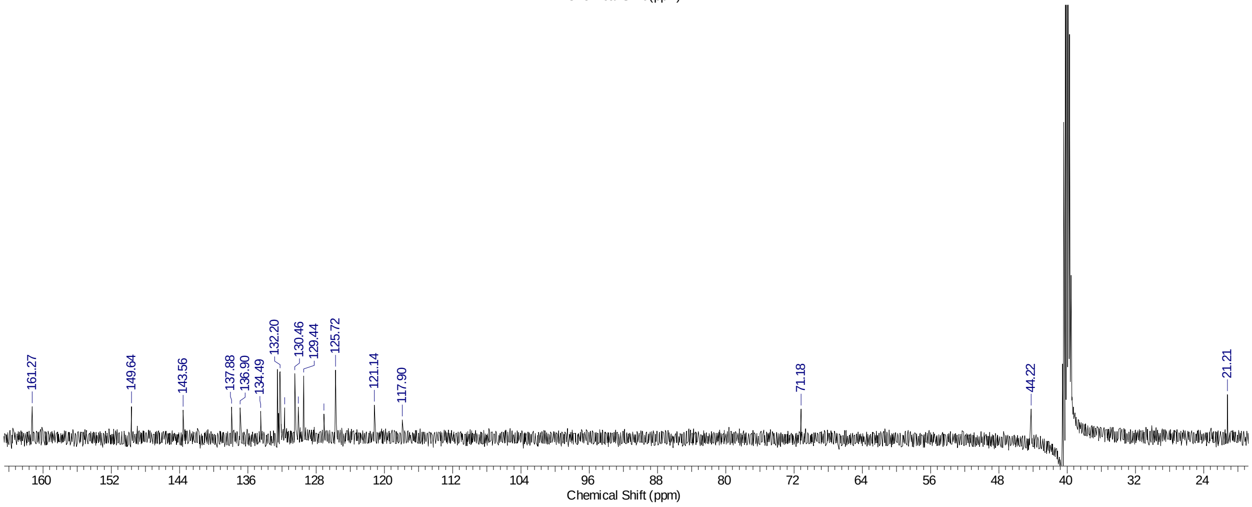

Synthesis of 5-amino-1,7-diaryl-3-(arylideneamino)-7*H*-pyrrolo[1,2-*c*]imidazole-6-carboxylates 16a,b

General procedure. A mixture of the corresponding 2-amino-4-arylimidazole **1** (1.0 mmol), aromatic aldehyde **2** (2.0 mmol) and ethyl 2-cyanoacetate **15** (1.0 mmol) in 2 mL of 2-propanol was refluxed during 20–30 min. After cooling, the yellow solid products **16** were filtered off and crystallized from iPrOH.

Ethyl 5-amino-3-(benzylideneamino)-1,7-diphenyl-7H-pyrrolo[1,2-c]imidazole-6-carboxylate (16a)

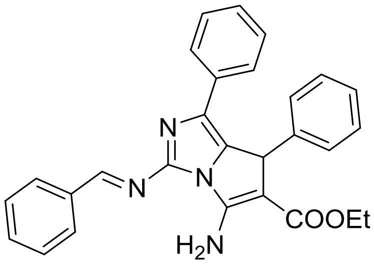

Yellow solid, 30%, mp 239-240 °C, ^{1}H NMR (200 MHz, DMSO-d_6) δ: 9.32 (s, 1H, $CH_{azomethine}$), 8.12 (d, 2H, Ar, *J*=6.7), 7.67-7.45 (m, 5H, Ar), 7.27-7.04 (m, 10H, C^5NH_2, Ar), 5.15 (s, 1H, C^7H, *J*=6.7), 4.05-3.84 (m, 2H, O$\underline{CH_2}$$CH_3$), 1.01 (s, 3H, *J* 7.0, $OCH_2$$\underline{CH_3}$); ^{13}C NMR (125 MHz, DMSO-d_6) δ: 178.7 (CO), 134.5, 133.3, 130.2, 129.6, 128.7, 128.6, 128.3, 127.8, 127.2, 127.0, 126.4, 125.8, 125.5, 116.7, 93.4, 58.9, 43.4, 14.7; MS (m/z) (%): 448([$M^{+\bullet}$], 100. Anal. calcd. for $C_{28}H_{24}N_4O_2$ (448.19) C 74.98; H 5.39; N 12.49; Found: C 75.12; H 4.89; N 11.37.

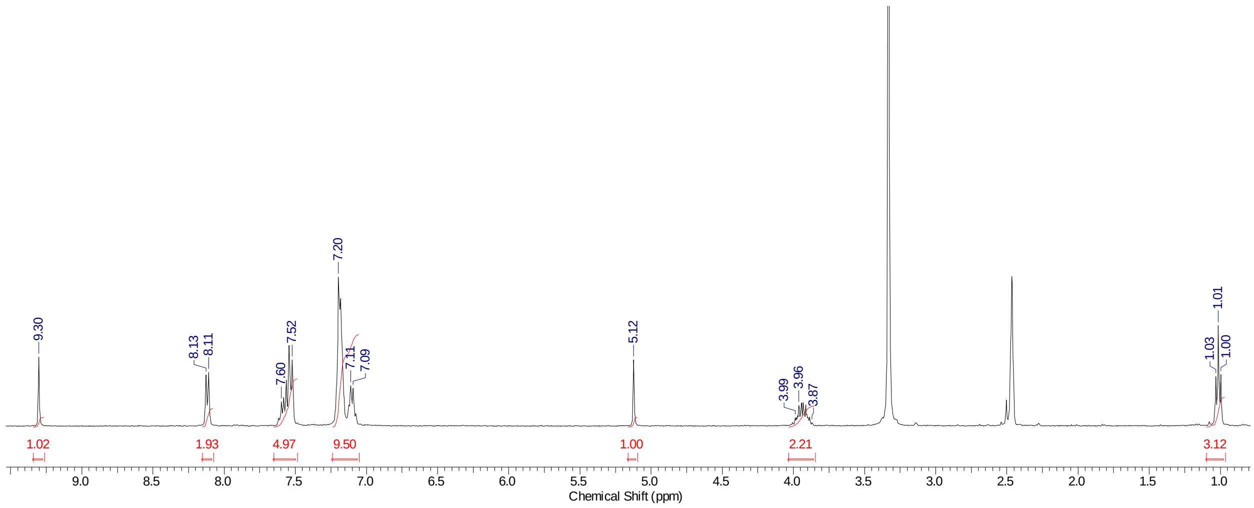

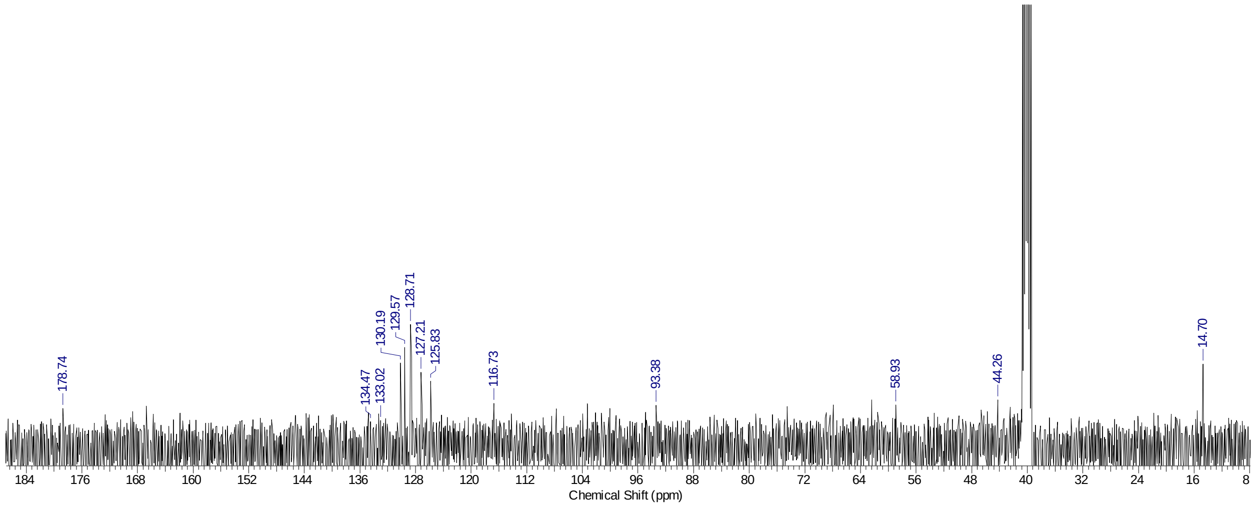

Ethyl 5-amino-3-((4-bromobenzylidene)amino)-7-(4-bromophenyl)-1-phenyl-7H-pyrrolo[1,2-c]imidazole-6-carboxylate (16b)

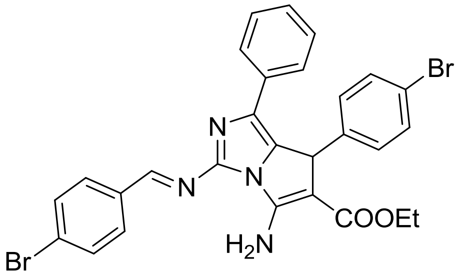

Yellow powder, 40%, m.p. 249-250 °C. ^{1}H NMR (200 MHz, DMSO-d_6) δ: 9.29 (s, 1H, $CH_{azomethine}$), 8.08 (d, 2H, Ar, J=7.6), 7.75 (d, 2H, Ar, J=7.6), 7.54 (d, 2H, Ar, J=7.3), 7.37 (d, 2H, Ar, J=7.6), 7.25-7.09 (m, 5H, C^5NH_2, Ar), 5.19 (s, 1H, C^7H), 4.05-3.86 (m, 2H, $O\underline{CH_2}CH_3$), 1.04 (s, 3H, J 6.4, $OCH_2\underline{CH_3}$); ^{13}C NMR (125 MHz, DMSO-d_6) δ: 165.7 (CO), 160.9, 154.9, 148.8, 143.7, 139.8, 134.6, 133.8, 132.6, 131.9, 131.5, 131.4, 128.8, 127.8, 127.1, 125.8, 120.1, , 93.0, 59.0, 43.5, 14.7; MS (m/z) (%): 605 ($[M^{+\bullet}]$, 50), 489, 473, 347.4, 153. Anal. calcd. for $C_{28}H_{22}Br_2N_4O_2$ (606.31) C 55.47; H 3.66; N 9.24; Found, %: C 55.67; H 5.19; N 8.65.

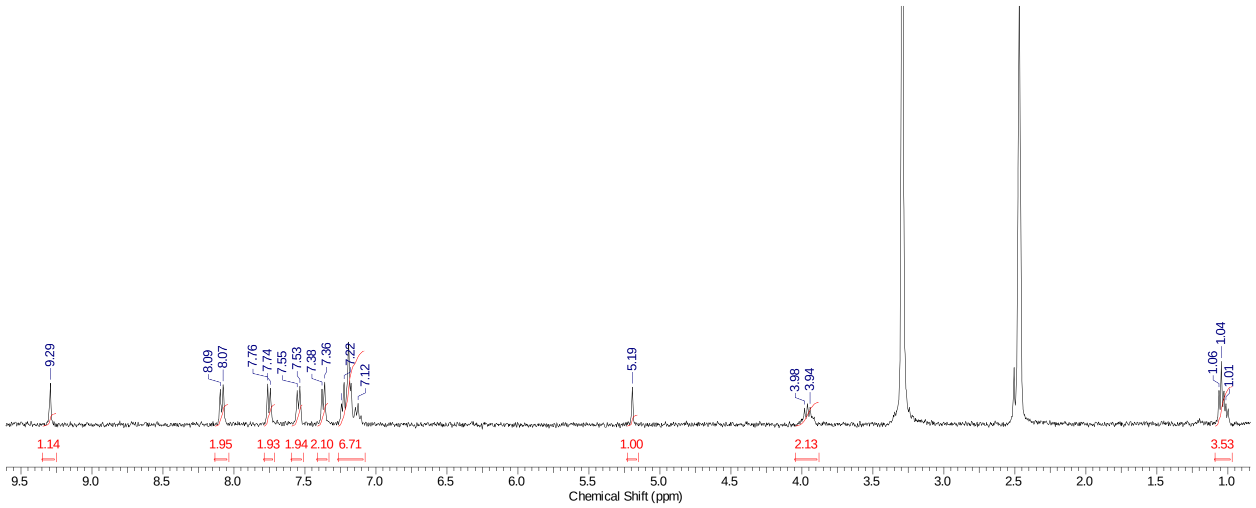

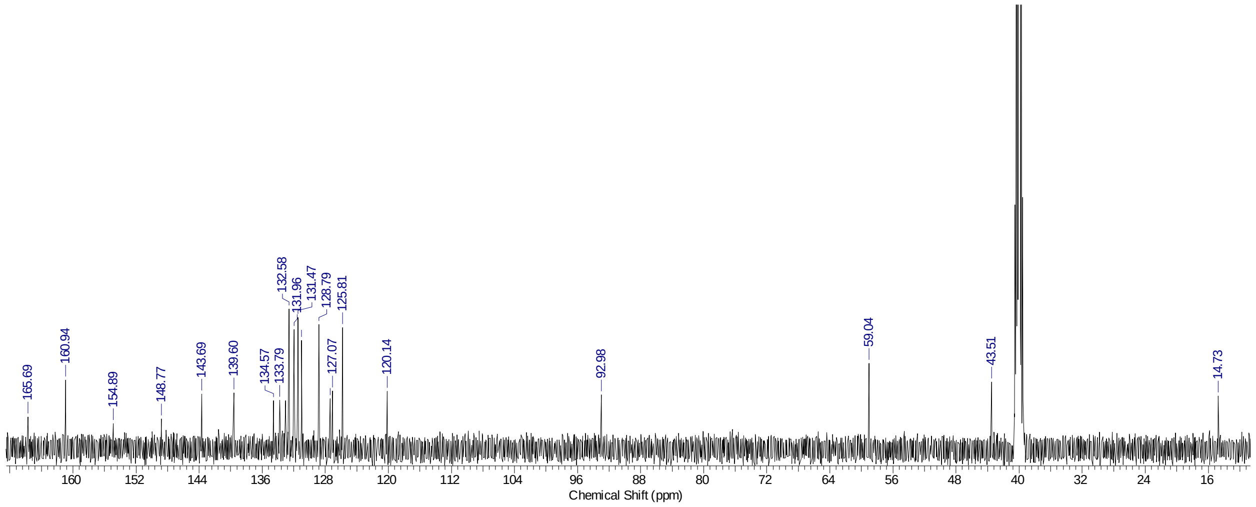

Synthesis of 3',5'-diamino-1-alkyl-2-oxo-1'-arylspiro[indolin-3,7'-pyrrolo[1,2-c]imidazole]-6'-carbonitriles 19a-h

General procedure. The mixture of corresponding 2-amino-4-arylimidazoles **1** (1.0 mmol), isatin **18** (1.0 mmol) and malononitrile **12** (1.0 mmol) in 2 ml of 2-propanol was refluxed during 50-60 min. After cooling, the solid products **19** were filtered off and crystallized from iPrOH.

3',5'-Diamino-1-methyl-2-oxo-1'-phenylspiro[indoline-3,7'-pyrrolo[1,2-c]imidazole]-6'-carbonitrile (19a)

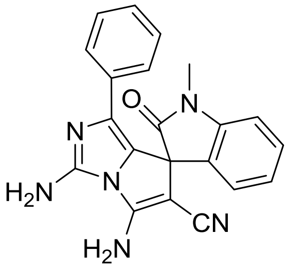

Colorless solid, 60%, mp 250-252 °C, ^{1}H NMR (200 MHz, DMSO-d_6) δ: 7.77 (br.s, 2H, $C^{5'}NH_2$), 7.37 (t, 1H, Ar_{isatin}, J=7.9), 7.24-7.10 (m, 2H, Ar), 7.10-6.95 (m, 4H, Ar), 6.94-6.82 (m, 2H, Ar), 6.47 (br.s, 2H, $C^{3'}NH_{2imidazole}$), 3.21 (s, 3H, $N^{1}CH_3$); ^{13}C NMR (125 MHz, DMSO-d_6) δ: 176.3 (C^2), 154.2 ($C^{5'}$), 146.4 ($C^{3'}$), 145.7, 135.4, 133.0, 132.2, 130.7, 130.27, 129.0, 127.2, 126.6, 126.1, 126.0, 111.7, 69.8 ($C^{6'}$), 55.8 (C_{spiro}), 29.2 ($N^{1}CH_3$); MS (m/z) (%):369 $[M+H]^+$ (100); Anal. calcd. for $C_{21}H_{16}N_6O$ (368.14) C 68.47; H 4.38; N 22.81. Found: C 69.43; H 5.07; N 22.64.

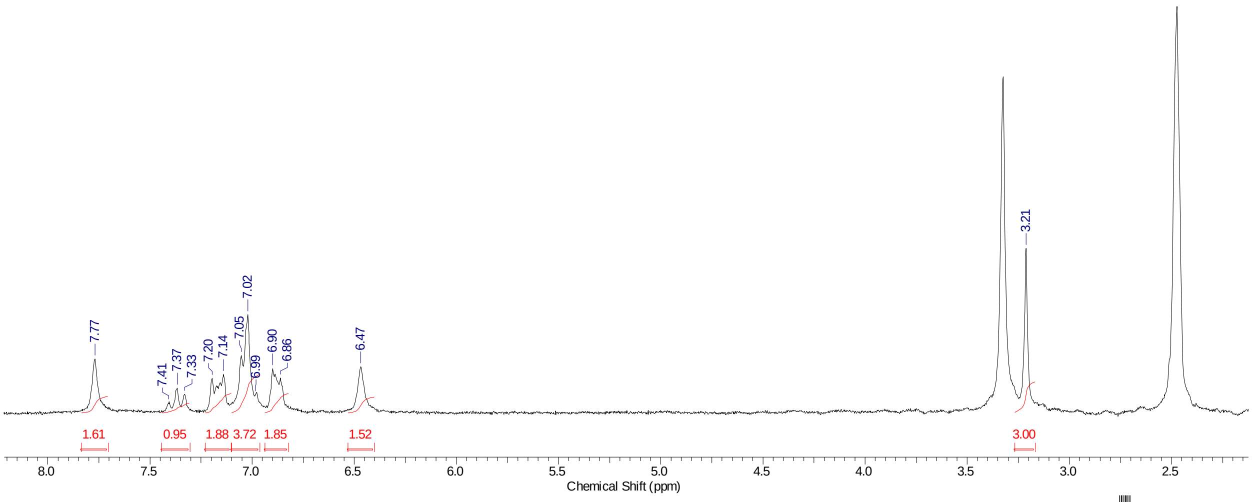

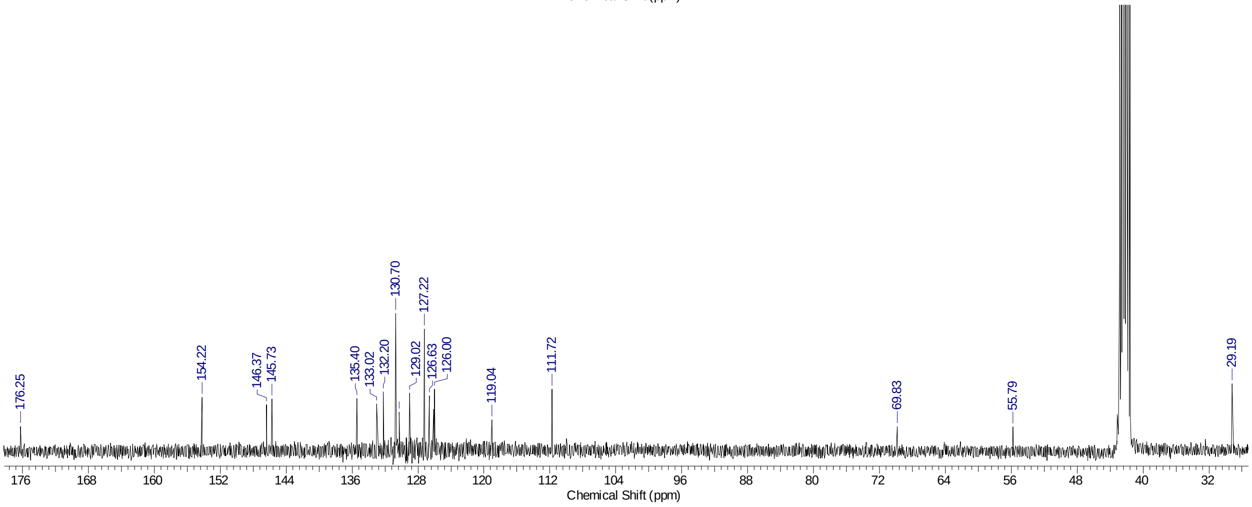

3',5'-Diamino-5-bromo-1-methyl-2-oxo-1'-phenylspiro[indoline-3,7'-pyrrolo[1,2-c]imidazole]-6'-carbonitrile (19b)

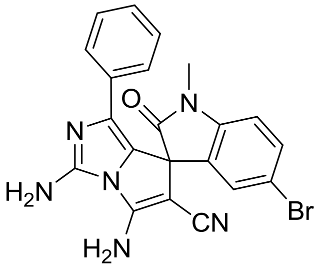

Colorless solid, 68%, m.p. 256-258 °C. ^1H NMR (200 MHz, DMSO-d_6) δ: 7.81 (br.s, 2H, $C^{5'}$NH2), 7.57 (dd, 1H, H^6_{isatin}, *J*=1.8, *J*=8.1), 7.44 (d, 1H, H^4_{isatin}, *J*=1.8), 7.17 (d, 1H, H^7_{isatin}, *J*=8.4), 7.12-7.01 (m, 3H, $Ar_{imidazole}$); 6.94-6.85 (m, 2H, $Ar_{imidazole}$), 6.46 (br.s, 2H, $C^{3'}$-NH_2), 3.22 (s, 3H, N^1CH_3); ^{13}C NMR (125 MHz, DMSO-d_6) δ: 173.8 (C^2), 152.3 ($C^{5'}$), 143.9 ($C^{3'}$), 143.5, 133.2, 132.9, 131.0, 130.7, 128.8, 127.5, 127.1, 125.0, 123.1, 117.1, 115.7, 111.7, 66.9 ($C^{6'}$), 53.5 (C_{spiro}), 27.3 (N^1CH_3). MS (m/z) (%): 449 $[M(^{81}Br)+H]^+$ (80), 447 $[M(^{79}Br)+H]^+$ (72), 72 (100). Anal. calcd. for $C_{21}H_{15}BrN_6O$ (447.29) C 56.39; H 3.38; N 18.79. Found, %: C 63.19; H 3.46; N 19.23.

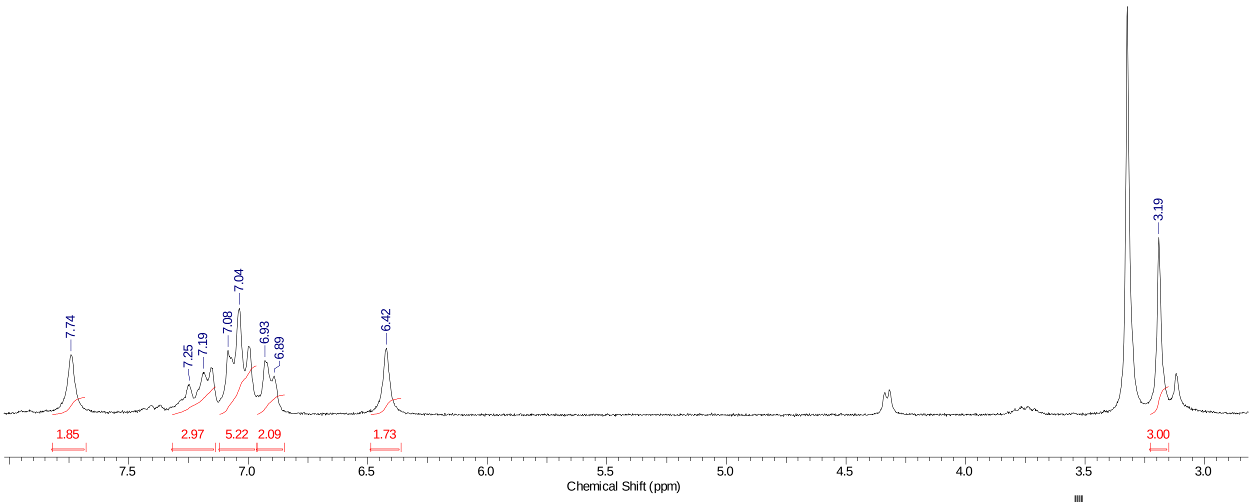

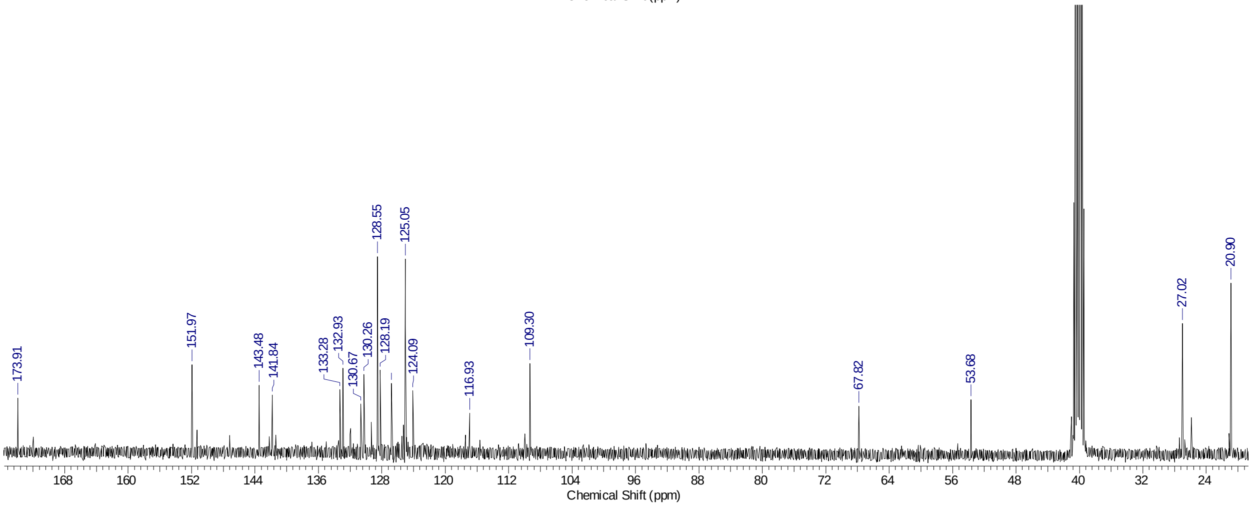

3',5'-Diamino-1,5-dimethyl-2-oxo-1'-phenylspiro[indoline-3,7'-pyrrolo[1,2-c]imidazole]-6'-carbonitrile (19c)

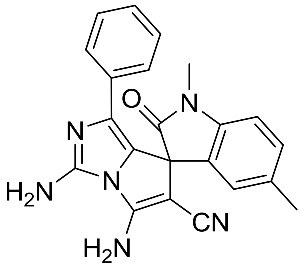

Colorless solid, 60%, m.p. 276-278 °C. ^{1}H NMR (200 MHz, DMSO-d_6) δ: 7.74 (br.s 2H, $C^{5'}NH_2$), 7.28-7.13 (m, 2H, Ar), 7.12-6.98 (m, 4H, Ar), 6.96-6.85 (m, 2H, Ar), 6.42 (br.s., 2H, $C^{3'}NH_2$), 3.19 (s, 3H, N^1CH_3), 2.19 (s, 3H, C^5CH_3). ^{13}C NMR (100 MHz, DMSO-d_6) δ: 173.9 (C^2), 152.0 ($C^{5'}$), 143.5 ($C^{3'}$), 141.8, 133.3, 132.9, 130.7, 130.3, 128.2, 126.8, 125.1, 125.0, 124.1, 116.9, 109.3, 67.8 ($C^{6'}$), 53.7 (C_{spiro}), 27.0 (N^1CH_3), 20.9 (C^5CH_3). MS (m/z) (%): 383 $[M+H]^+$ (100). Anal. calcd. for $C_{22}H_{18}N_6O$ (382.42) C 69.10; H 4.74; N 21.98; Found, %: C 69.79; H 5.07; N 22.66.

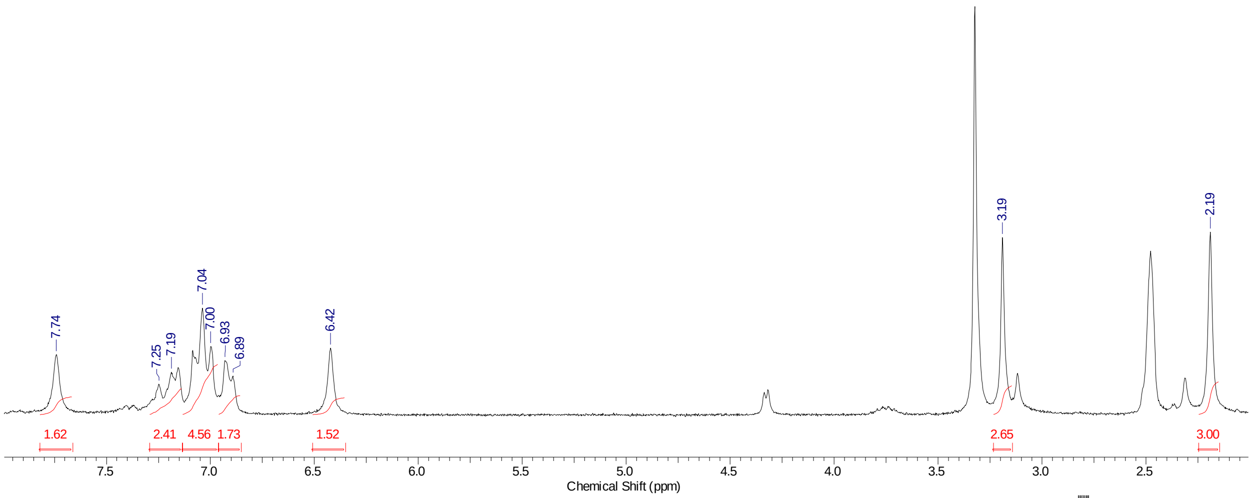

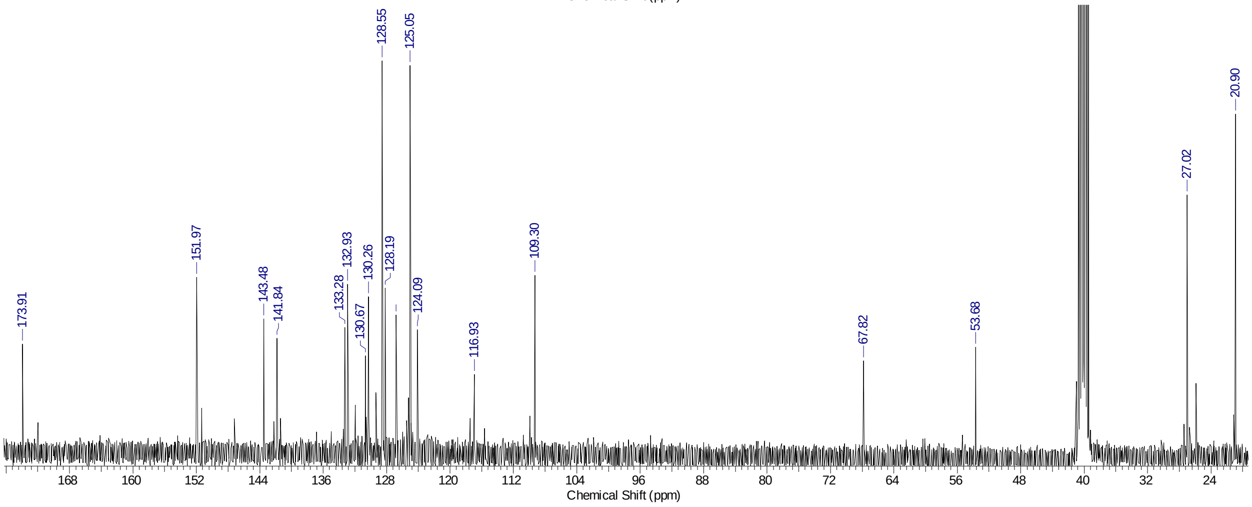

3',5'-Diamino-1-(4-chlorobenzyl)-5-fluoro-2-oxo-1'-phenylspiro[indoline-3,7'-pyrrolo[1,2-c]imidazole]-6'-carbonitrile (19d)

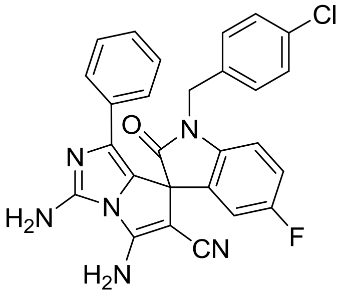

Colorless solid, 40 %, m.p.. 248-250 °C. ^{1}H NMR (200 MHz, DMSO-d_6) δ: 7.84 (br.s, 2H, $C^{5'}NH_2$), 7.43-7.28 (m, 4H, $CH_2\underline{C_6H_4}Cl$), 7.23-7.11 (m, 3H, Ar), 7.03-6.93 (m, 1H, Ar), 6.93-6.81 (m, 4H, Ar), 6.49 (br.s, 2H, $C^{3'}NH_2$), 5.08-4.88 (m, 2H, $\underline{CH_2}C_6H_4Cl$); ^{13}C NMR (125 MHz, DMSO-d_6) δ: 176.6 (C^2), 162.9 (C^4F), 160.5 (C^4F), 154.3 ($C^{5'}$), 146.0 ($C^{3'}$), 141.5, 137.4, 135.1, 134.9, 133.4, 132.1, 131.2, 130.7, 129.1, 127.3, 125.0, 119.0, 118.4, 114.8, 113.3, 69.8 ($C^{6'}$), 56.0 ($\underline{CH_2}C_6H_4Cl$), 45.6 (C_{cnipo}). MS (m/z) (%): 497 $[M+H]^+$ (88), 125 (100).

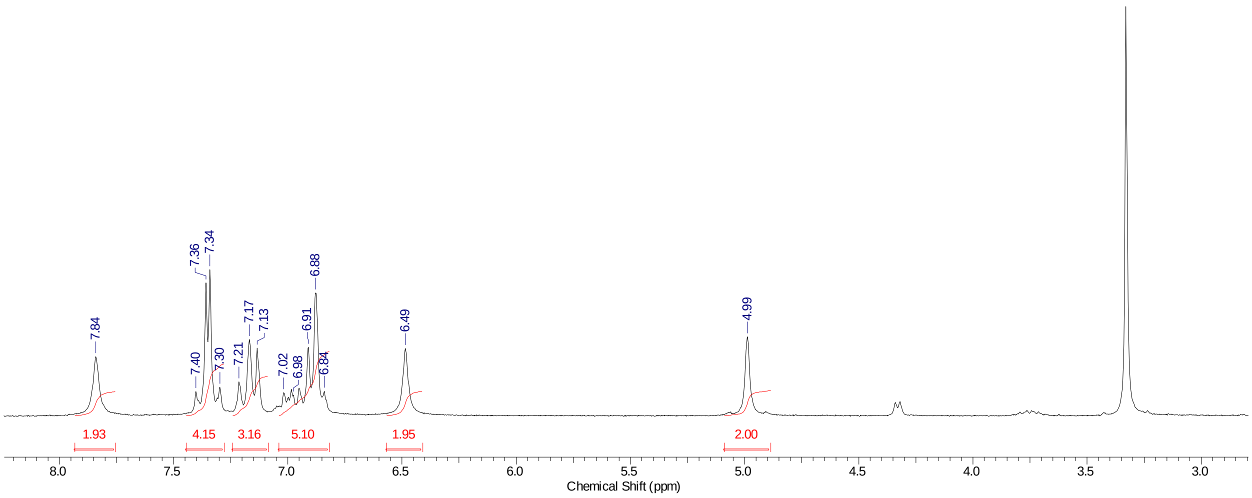

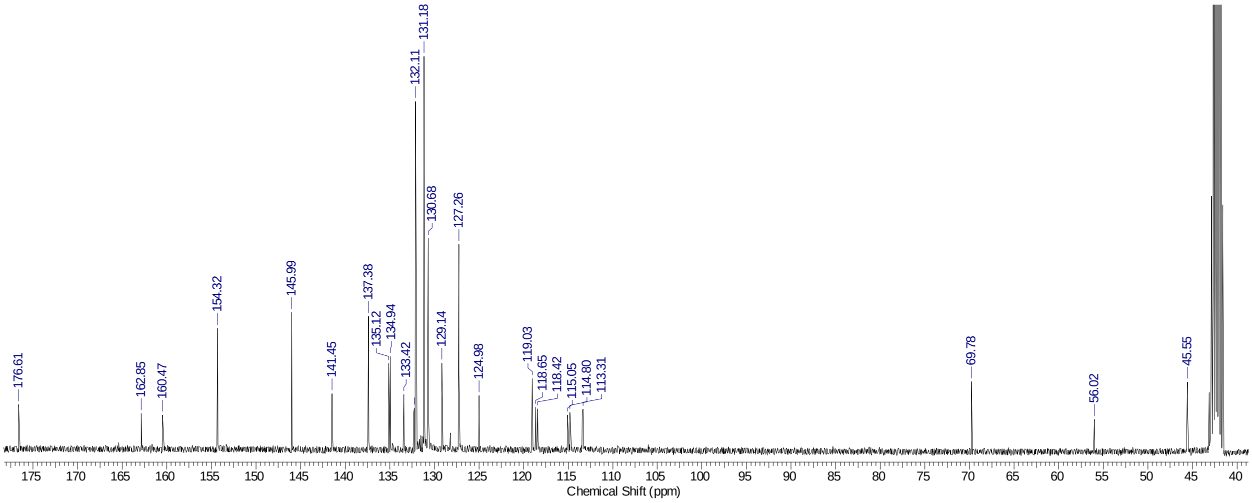

3',5'-Diamino-1-methyl-2-oxo-1'-(p-tolyl)spiro[indoline-3,7'-pyrrolo[1,2-c]imidazole]-6'-carbonitrile (19e)

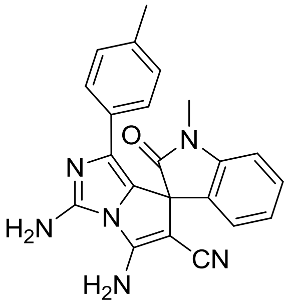

Colorless solid, 68%, m.p. 270-272 °C. ^{1}H NMR (200 MHz, DMSO-d_6) δ: 7.65 (br.s, 2H, $C^{5'}NH_2$), 7.35 (t, 1H, Ar_{isatin}, J=7.6), 7.17-7.09 (m, 2H, Ar_{isatin}), 7.01 (t, 1H, Ar_{isatin}, J=7.3), 6.85 (d, 2H, 2,6$Ar_{imidazole}$, J=8.1), 6.78 (d, 2H, $Ar^{3,5}{}_{imidazole}$, J=8.3), 6.31 (br.s, 2H, $C^{3'}$-NH_2), 3.20 (s, 3H, N^1CH_3), 2.12 (s, 3H, $C^{1'}H_{3imidazole}$); ^{13}C NMR (125 MHz, DMSO-d_6) δ: 176.33 (C^2), 154.21 ($C^{5'}$), 146.37, 145.61($C^{3'}$), 138.29, 133.04, 132.66, 132.15, 131.29, 130.38, 127.19, 126.63, 125.96, 125.45, 119.10, 111.69, 69.82 ($C^{6'}$), 55.77 (C_{spiro}), 29.19 (N^1CH_3), 23.14 (CH_3 $Ar_{imdazole}$). MS (m/z) (%): 383 $[M+H]^+$ (100). Anal. calcd. for $C_{22}H_{18}N_6O$ (382.42) C 69.10; H 4.74; N 21.98; Found, %: C 70.15; H 6.89; N 23.28.

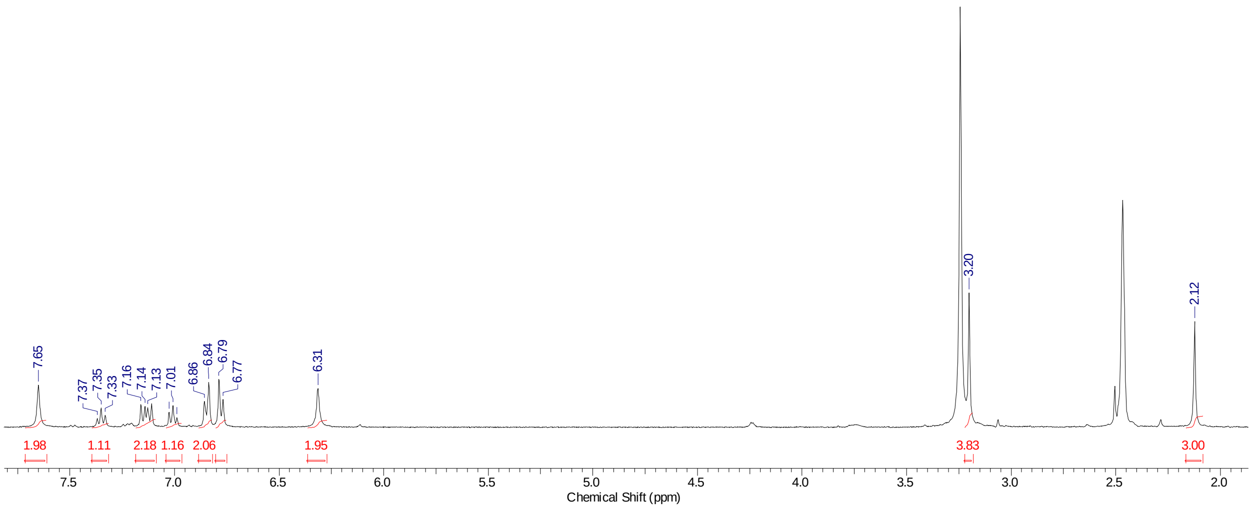

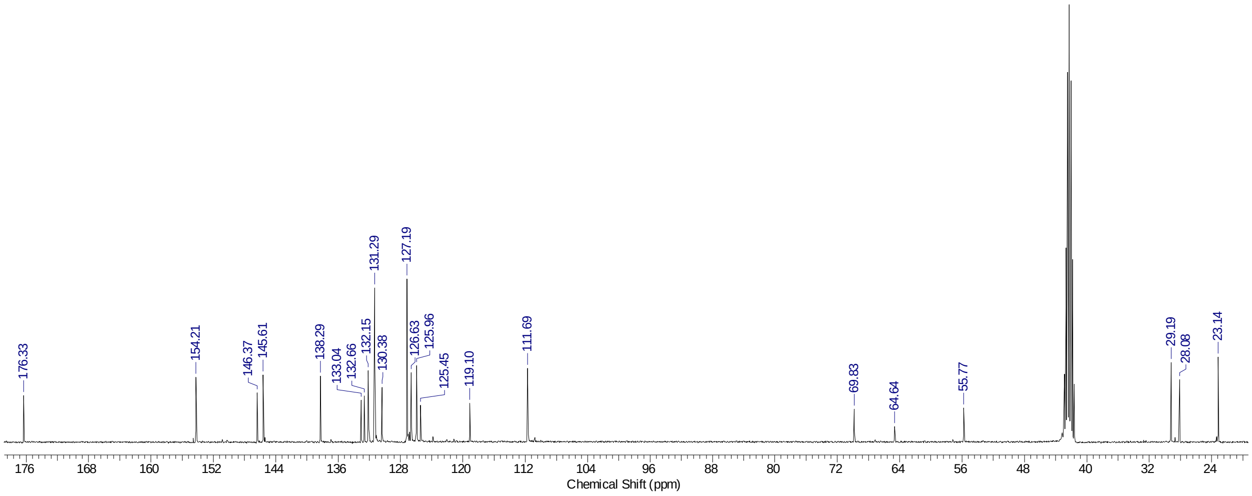

3',5'-Diamino-5-bromo-1-methyl-2-oxo-1'-(p-tolyl)spiro[indoline-3,7'-pyrrolo[1,2-c]imidazole]-6'-carbonitrile (19f)

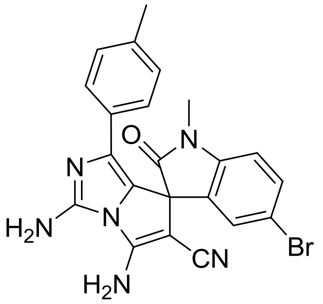

Colorless solid, 65%, m.p. 278-280 °C. ^{1}H NMR (200 MHz, DMSO-d_6) δ: 7.72 (br.s, 2H, $C^{5'}NH_2$), 7.54 (d, 1H, Ar^{6}_{isatin}, *J*=8.3), 7.38 (s, 1H, Ar^{4}_{isatin}), 7.14 (d, 1H, $Ar7_{isatin}$, *J*=8.3), 6.89 (d, 2H, $Ar^{2,6}_{imidazole}$, *J*=8.0), 6.79 (d, 2H, $Ar^{3,5}_{imidazole}$, *J*=8.0), 6.37 (br.s, 2H, $C^{3'}NH_2$), 3.21 (s, 3H, $N^{1}CH_3$); 2.14 (s, 3H, $C^{1'}H_{3imidazole}$). ^{13}C NMR (125 MHz, DMSO-d_6) δ: 173.8 (C^{2}), 152.2 ($C^{5'}$), 143.6 ($C^{3'}$), 136.4, 133.6, 131.9, 131.0, 130.7, 130.4, 130.1, 128.5, 128.1, 125.7, 124.1, 122.5, 119.1, 116.9, 115.5, 112.4, 110.8, 66.9 ($C^{6'}$), 53.5 (C_{spiro}), 27.9 ($N^{1}CH_3$), 26.5 (CH_3 $Ar_{imidazole}$). MS (m/z) (%): 463 $[M(^{81}Br)+H]^+$ (48), 461 $[M(^{79}Br)+H]^+$ (35), 72 (100). Anal. calcd. for $C_{22}H_{17}BrN_6O$ (461.31) C 57.28; H 3.71; N 18.22; Found, %: C 57.98; H 6.09; N 15.21.

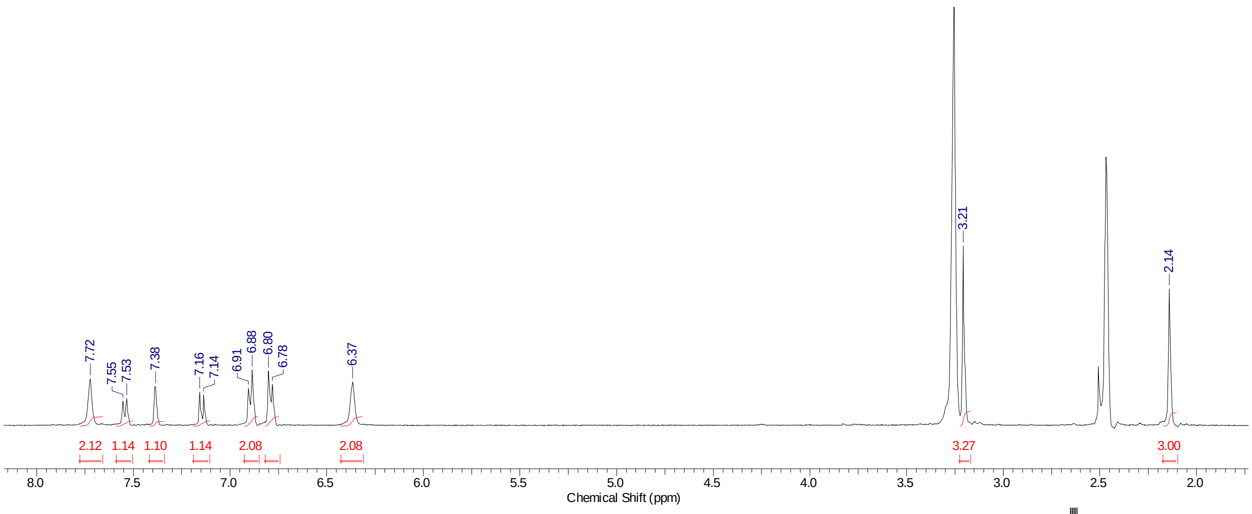

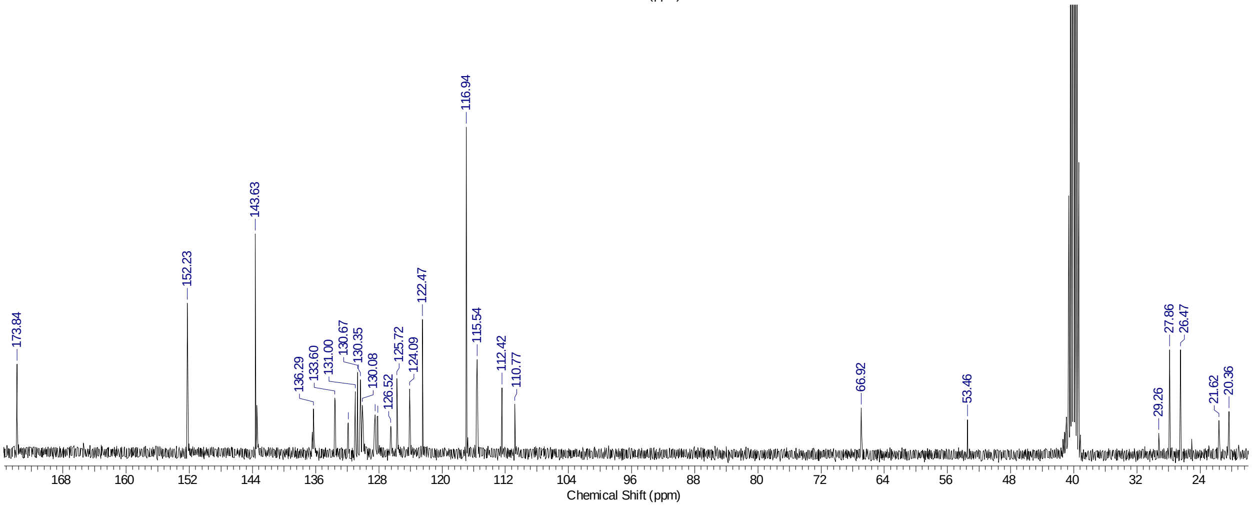

3',5'-Diamino-5-bromo-1'-(4-fluorophenyl)-1-methyl-2-oxospiro[indoline-3,7'-pyrrolo[1,2-c]imidazole]-6'-carbonitrile (19g)

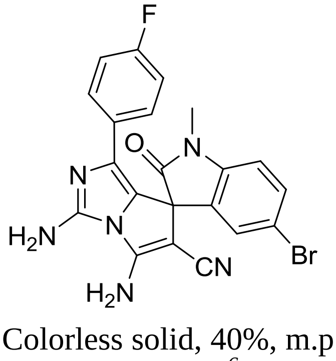

Colorless solid, 40%, m.p. 278-280 °C. ^{1}H NMR (200 MHz, DMSO-d_6) δ: 7.74 (br.s, 2H, $C^{5'}NH_2$), 7.55 (d, 1H, Ar^6_{isatin}, *J*=8.3), 7.40 (s 1H, Ar^4_{isatin}), 7.14 (d 1H, Ar^7_{isatin}, *J*=8.3), 6.99-6.86 (m, 4H, Ar); 6.41 (br.s, 2H, $C^{3'}NH_2$), 3.22 (s, 3H, N^1CH_3); ^{13}C NMR (125 MHz, DMSO-d_6) δ: 173.8 (C^2), 162.5 (C^4F), 160.1 (C^4F), 152.4 ($C^{5'}$), 143.9 ($C^{3'}$), 143.4, 132.9, 130.5, 130.1, 129.8, 127.5, 126.8, 122.9, 117.1, 115.9, 115.7, 111.8, 66.8 ($C^{6'}$), 53.4 (C_{spiro}), 27.3 (N^1CH_3). MS (m/z) (%): 467 $[M(^{81}Br)+H]^+$ (8), 465 $[M(^{79}Br)+H]^+$ (5), 456 (100).

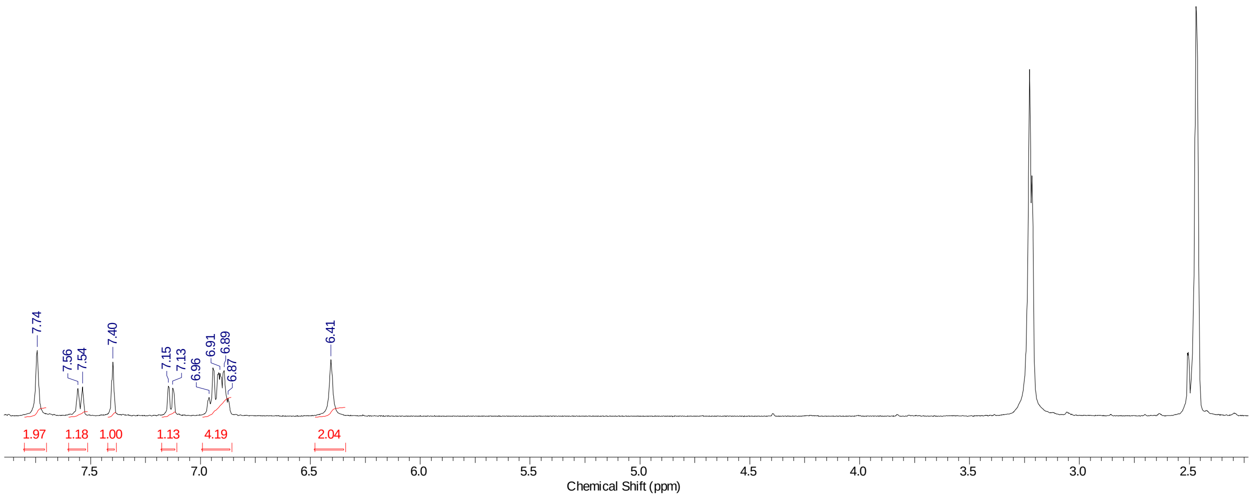

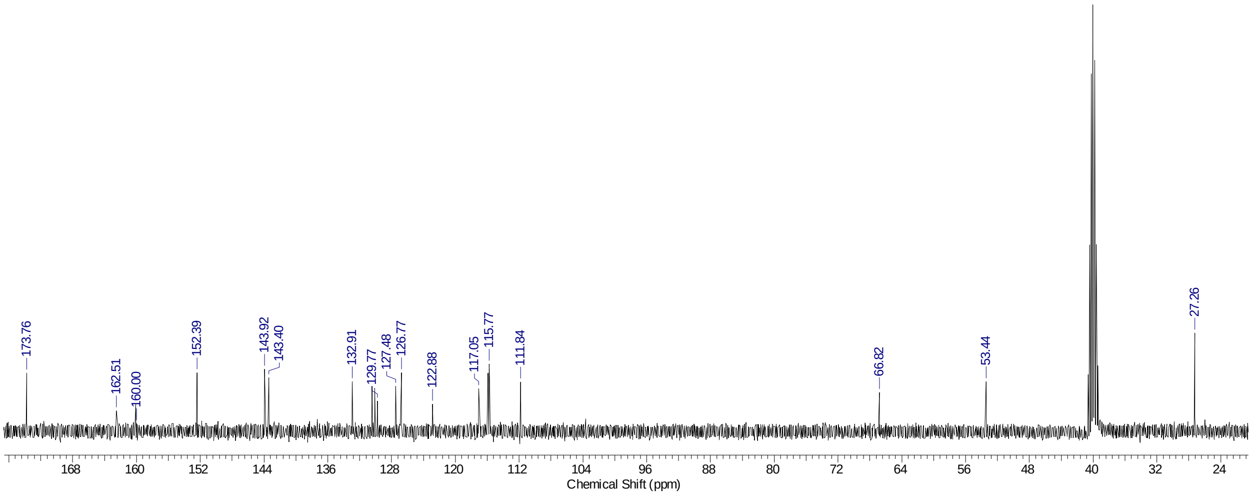

3',5'-Diamino-5-chloro-1-methyl-2-oxo-1'-(p-tolyl)spiro[indoline-3,7'-pyrrolo[1,2-c]imidazole]-6'-carbonitrile (19h)

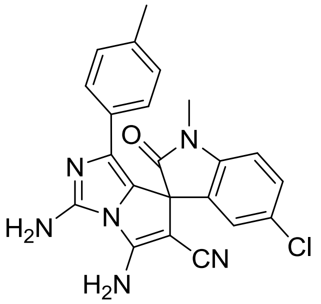

Colorless solid, 48%, m.p. 310-312 °C. ^{1}H NMR (200 MHz, DMSO-d_6) δ: 7.71 (br.s, 2H, $C^{5'}NH_2$), 7.41 (d, 1H, Ar^{6}_{isatin} J=8.2), 7.27 (s, 1H, Ar^{4}_{isatin}), 7.18 (d, 1H, Ar^{7}_{isatin}, J=8.6), 6.89 (d, 2H, $Ar^{2,6}_{imidazole}$, J=7.9), 6.79 (d, 2H, $Ar^{3,5}_{imidazole}$, J=7.9), 6.35 (br.s, 2H, $C^{3'}NH_2$), 3.21 (s, 3H, $N^{1}CH_3$), 2.14 (s, 3H, $Ar_{imidazile}$ CH_3). ^{13}C NMR (125 MHz, DMSO-d_6) δ: 174.0 (C^2), 152.3 ($C^{5'}$), 143.7 ($C^{3'}$), 143.1, 136.4, 131.0, 130.4, 130.0, 129.4, 128.0, 125.0, 124.8, 122.5, 117.1, 111.2, 66.9 ($C^{6'}$), 53.5 (C_{spiro}), 27.3 (N^1-CH_3), 21.1 ($Ar_{imidazole}CH_3$). MS (m/z) (%): 417 $[M+H]^+$ (100). Anal. calcd. for $C_{22}H_{17}ClN_6O$. (416.86) C 63.39; H 4.11; N 20.16; Found, %: C 65.89; H 3.26; N 20.28.

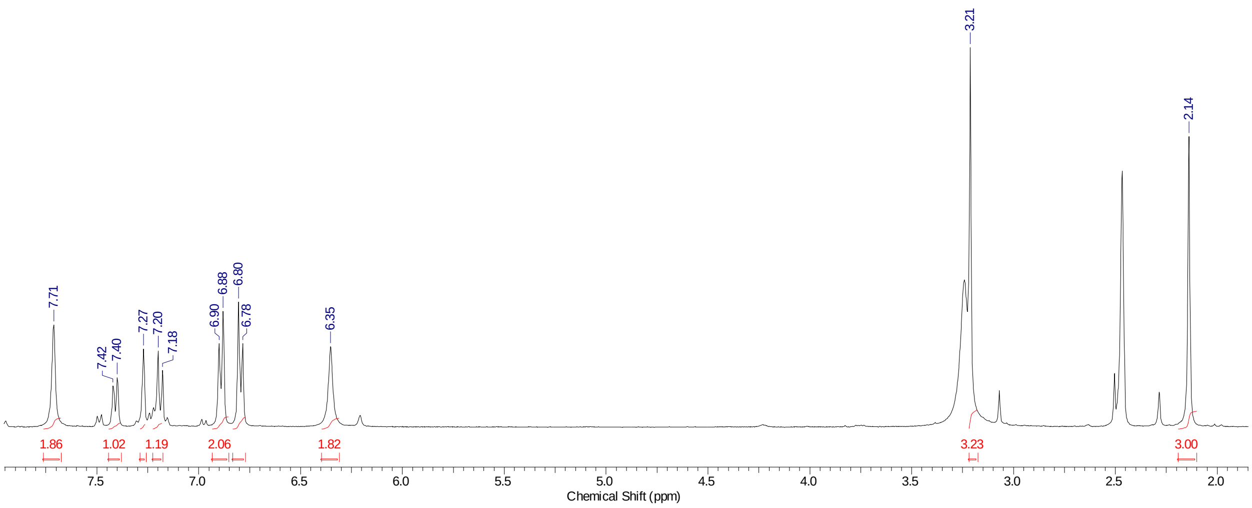

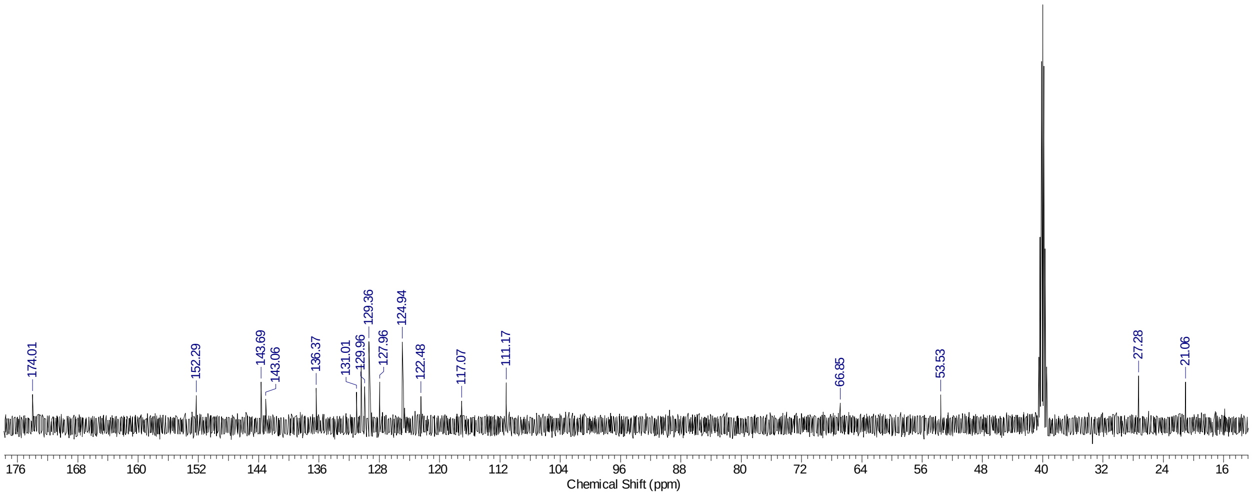

Synthesis of 3',5'-diamino-1-alkyl-2-oxo-1'-arylspiro[indoline-3,7'-pyrrolo[1,2-*c*]imidazole]-6'-carboxylates 20a-c

General procedure. The mixture of corresponding 2-amino-4-arylimidazoles **1** (1.0 mmol), isatin **18** (1.0 mmol) and ethyl 2-cyanoacetate **15** (1.0 mmol) in 2 ml of 2-propanol was refluxed during 50–60 min. After cooling, the solid products **20** were filtered off and crystallized from iPrOH.

Ethyl 3',5'-diamino-1-methyl-2-oxo-1'-phenylspiro[indoline-3,7'-pyrrolo[1,2-c]imidazole]-6'-carboxylate (20a)

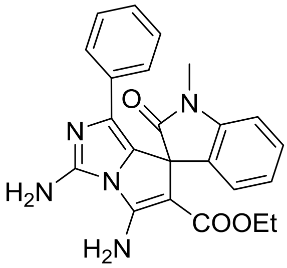

Colorless solid, 72%, mp 280-282 °C; ^{1}H NMR (200 MHz, DMSO-d_6) δ: 7.63 (br.s, 2H, $C^{5'}NH_2$), 7.29 (t, 1H, Ar, *J*=7.5), 7.11-6.88 (m, 8H, Ar), 6.46 (br.s, 2H, $C^{3'}NH_2$), 3.83-3.63 (m, 2H, CO$\underline{CH_2}$CH$_3$), 3.21 (s, 3H, N^1CH3), 0.88-0.69 (m, 3H, COCH$_2\underline{CH_3}$); ^{13}C NMR (125 MHz, DMSO-d_6) δ: 175.02 (C^2), 145.00 ($C^{5'}$), 143.72 ($C^{3'}$), 133.56, 130.38, 130.16, 128.98, 128.50, 126.69, 125.28, 124.96, 123.45, 123.11, 108.63, 58.50 ($C^{6'}$), 52.71 (C_{spiro}), 33.42 (CO$\underline{CH_2}$CH$_3$), 26.87 (N^1CH_3), 14.32 (COCH$_2\underline{CH_3}$); MS (m/z) (%): 416 $[M+H]^+$ (100). Anal. calcd. for $C_{23}H_{21}N_5O_3$ (415.16) C 66.49; H 5.09; N 16.86; Found, %: C 67.89; H 5.64; N 11.70.

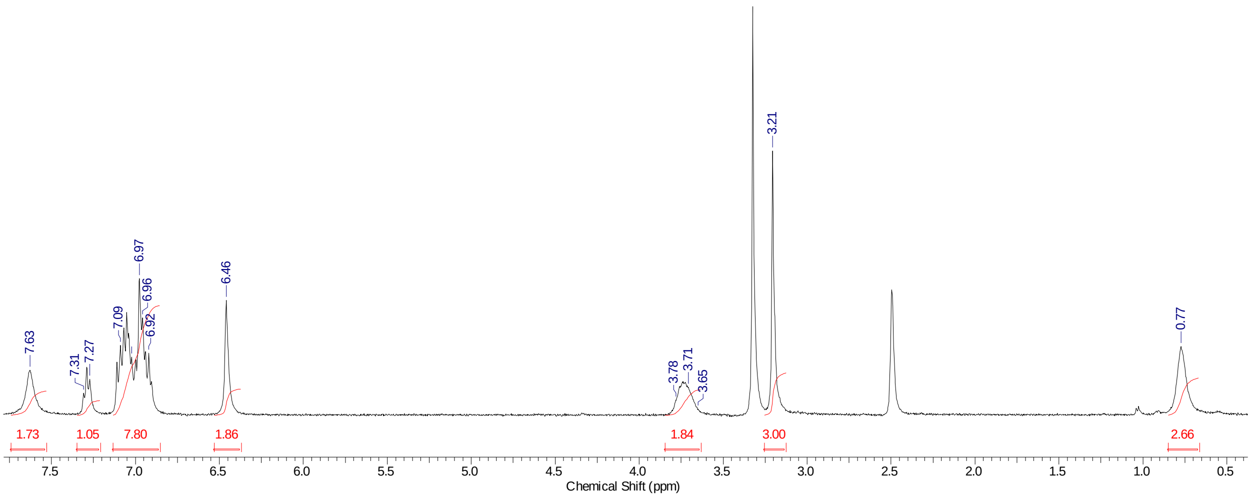

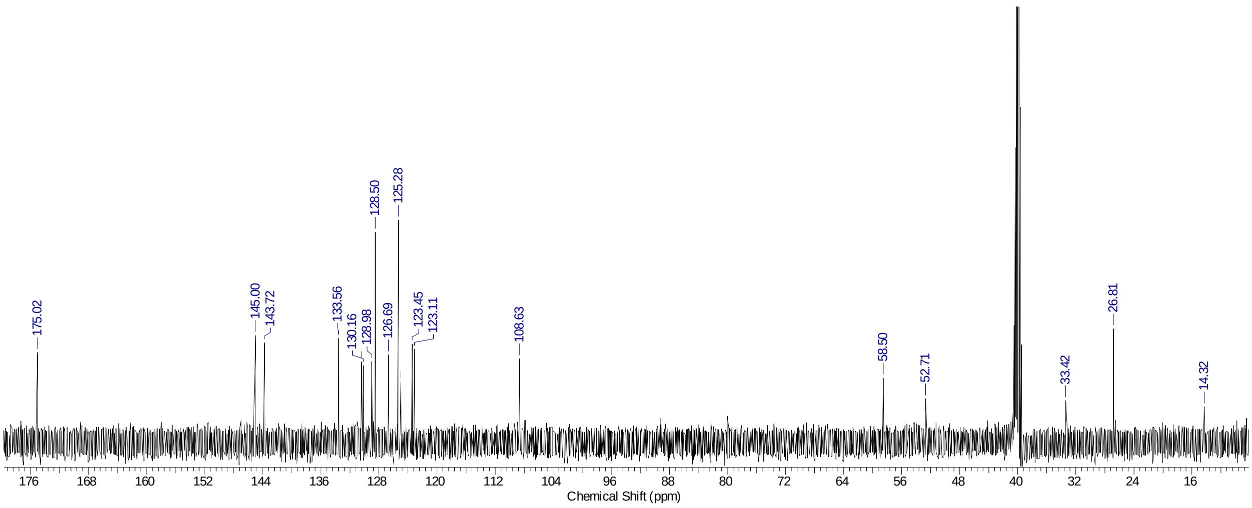

Ethyl 3',5'-diamino-5-bromo-1'-(4-methoxyphenyl)-1-methyl-2-oxospiro[indoline-3,7'-pyrrolo[1,2-c]imidazole]-6'-carboxylate (20b)

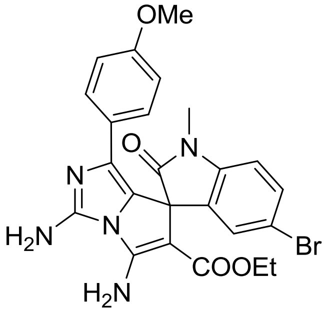

Brown solid, 43%, m.p. 262-264 °C. ^{1}H NMR (200 MHz, DMSO-d_6) δ: 7.67 (br.s, 2H, $C^{5'}NH_2$), 7.48 d, 1H, Ar^{6}_{isatin}, J=8.4), 7.20 (s, 1H, Ar^{4}_{isatin}), 7.08 (d, 1H, Ar^{7}_{isatin}, J=8.5), 6.91 (d, 2H, $Ar^{2,6}_{imidazole}$, J=8.0), 6.69 (d, 2H, $Ar^{3,5}_{imidazole}$, J=8.0), 6.46 (br.s, 2H, $C^{3'}NH_2$), 3.87-3.67 (m, 2H, CO$\underline{CH_2}$$CH_3$), 3.66 (s, 3H, $Ar_{imidazole}$ OCH_3), 3.22 (s, 3H, N^{1}CH3), 0.90-0.70 (m, 3H, $COCH_2\underline{CH_3}$). ^{13}C NMR (125 MHz, DMSO-d_6) δ: 174.9 (C^{2}), 158.4 ($C^{5'}$), 144.2, 143.8 ($C^{3'}$), 133.2, 131.6, 130.3, 126.5, 126.3, 126.1, 122.6, 114.8, 114.1, 110.7, 58.6 ($C^{6'}$), 55.5 ($Ar_{imidazole}$ OCH_3), 52.5 (C_{spiro}), 47.1 (CO$\underline{CH_2}$$CH_3$), 27.0 ($N^{1}CH_3$), 14.4 ($COCH_2\underline{CH_3}$). MS (m/z) (%): 526 $[M(^{81}Br)+H]^+$ (64), 524 $[M(^{79}Br)+H]^+$ (56), 91 (100). Anal. calcd. for $C_{24}H_{22}BrN_5O_4$ (524.37) C 54.97; H 4.23; N 13.36; Found, %: C 55.92; H 5.86; N 13.85.

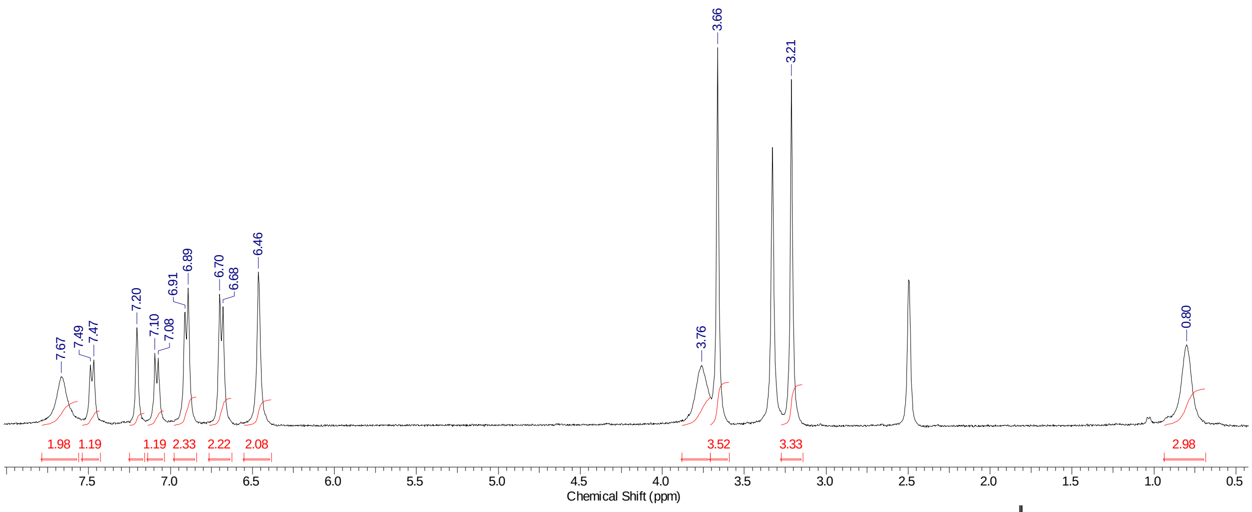

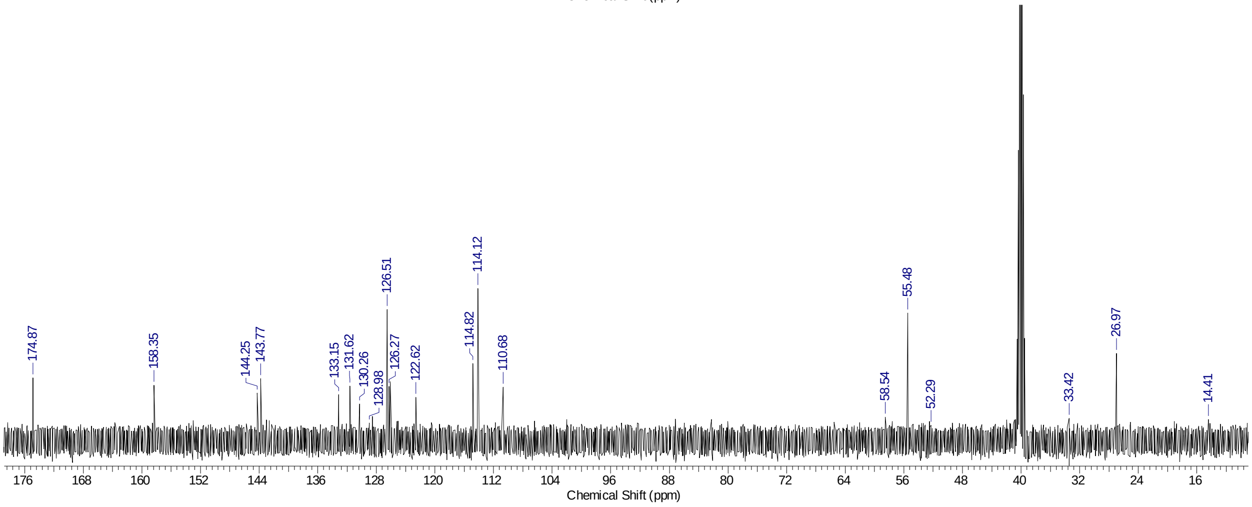

Ethyl 3',5'-diamino-5-bromo-1-methyl-2-oxo-1'-(p-tolyl)spiro[indoline-3,7'-pyrrolo[1,2-c]imidazole]-6'-carboxylate (20c).

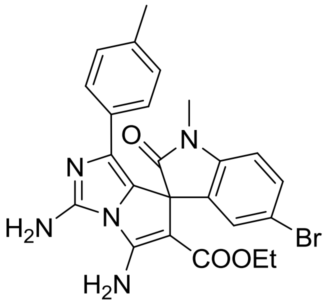

Colorless solid, 60%, m.p. 268-270 °C. ^{1}H NMR (200 MHz, DMSO-d_6) δ: 7.68 (br.s, 2H, $C^{5'}NH_2$), 7.48 (d, 1H, Ar^{6}_{isatin} J=8.4), 7.22 (s, 1H, Ar^{4}_{isatin}), 7.09 (d, 1H, Ar^{7}_{isatin}, J=8.4), 6.93 (d, 2H, $Ar^{2,6}_{imidazole}$, J=8.4), 6.87 (d, 2H, $Ar^{3,5}_{imidazole}$, J=8.4), 6.48 (br.s, 2H, $C^{3'}NH_2$), 3.85-3.68 (m, 2H, CO$\underline{CH_2}$$CH_3$), 3.22 (s, 3H, $N^{1}CH_3$), 2.18 (s, 3H, $Ar_{imidazole}$ CH_3), 0.91-0.70 (m, 3H, $COCH_2$$\underline{CH_3}$); ^{13}C NMR (125 MHz, DMSO-d_6) δ: 174.8 (C^{2}), 165.2 ($C^{5'}$), 144.3, 143.8 ($C^{3'}$), 136.1, 133.1, 131.6, 130.7, 130.4, 129.3, 128.4, 126.3, 125.2, 123.5, 114.8, 110.6, 58.6 ($C^{6'}$), 52.6 (C_{spiro}), 33.4 (CO$\underline{CH_2}$$CH_3$), 27.0 ($N^{1}CH_3$), 21.1 ($Ar_{imidazole}$ CH_3), 14.4 ($COCH_2$$\underline{CH_3}$),. MS (m/z) (%): 510 $[M(^{81}Br)+H]^+$ (8), 508 $[M(^{79}Br)+H]^+$ (7), 415 (100). Anal. calcd. for $C_{24}H_{22}BrN_5O_3$ (508.37) C 56.70; H 4.36; N 13.78; Found, %: C 58.11; H 5.51; N 15.08.

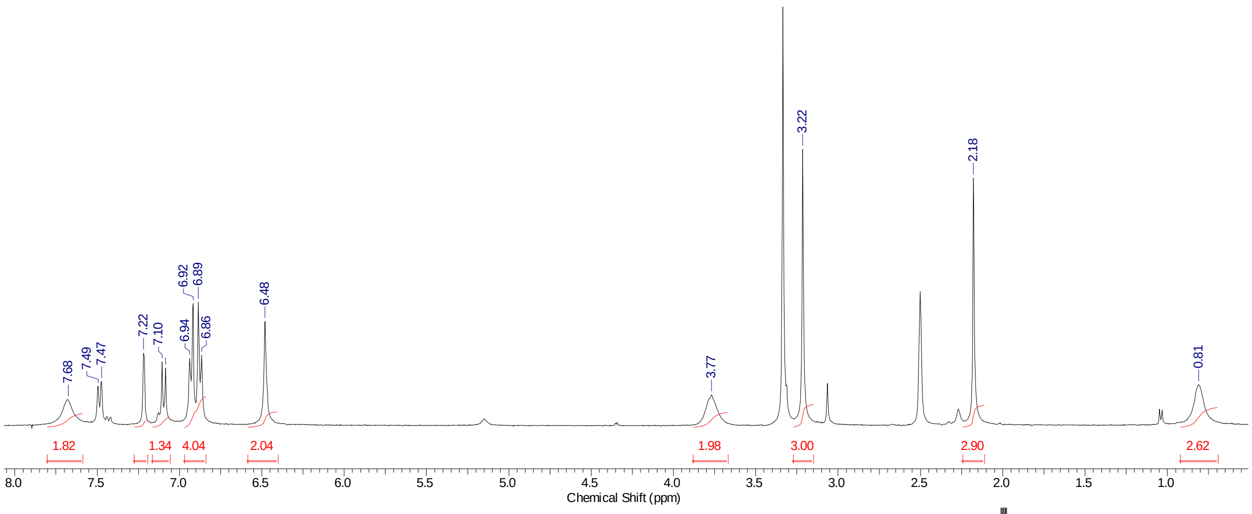

7.68
7.49
7.47
7.22
7.10
6.94
6.92
6.89
6.86
6.48
3.77
3.22
2.18
0.81
1.82
1.34
4.04
2.04
1.98
3.00
2.90
2.62
8.0
7.5
7.0
6.5
6.0
5.5
5.0
4.5
4.0
3.5
3.0
2.5
2.0
1.5
1.0
Chemical Shift (ppm)

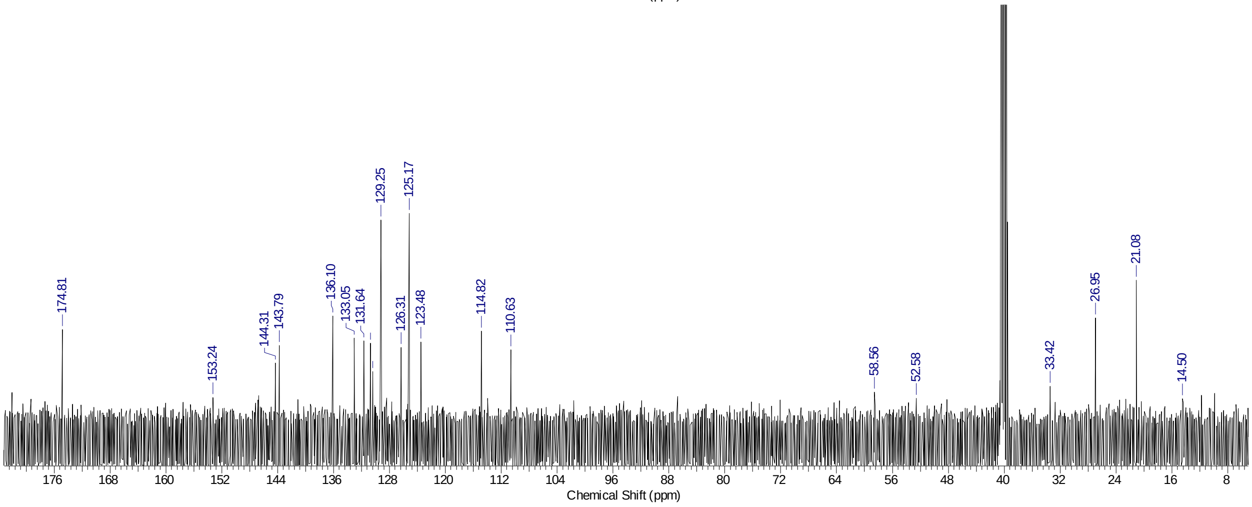

174.81
153.24
144.31
143.79
136.10
133.05
131.64
129.25
126.31
125.17
123.48
114.82
110.63
58.56
52.58
33.42
26.95
21.08
14.50
176
168
160
152
144
136
128
120
112
104
96
88
80
72
64
56
48
40
32
24
16
8
Chemical Shift (ppm)
